# Circadian VIPergic Neurons of the Suprachiasmatic Nuclei Sculpt the Sleep-Wake Cycle

**DOI:** 10.1016/j.neuron.2020.08.001

**Published:** 2020-11-11

**Authors:** Ben Collins, Sara Pierre-Ferrer, Christine Muheim, David Lukacsovich, Yuchen Cai, Andrea Spinnler, Carolina Gutierrez Herrera, Shao’Ang Wen, Jochen Winterer, Mino D.C. Belle, Hugh D. Piggins, Michael Hastings, Andrew Loudon, Jun Yan, Csaba Földy, Antoine Adamantidis, Steven A. Brown

**Affiliations:** 1 Chronobiology and Sleep Research Group, Institute of Pharmacology and Toxicology, University of Zürich, Winterthurerstrasse 190, 8057 Zürich, Switzerland; 2 Department of Biomedical Sciences, Washington State University, Spokane, WA 99202, USA; 3 Laboratory of Neural Connectivity, Brain Research Institute, University of Zürich, Winterthurerstrasse 190, 8057 Zürich, Switzerland; 4 Institute of Neuroscience, Chinese Academy of Sciences, 320 Yueyang Road, Shanghai 200031, P.R. China; 5 Department of Neurology, Inselspital University Hospital Bern, Freiburgstrasse 18, 3010 Bern, Switzerland; 6 Institute of Biomedical and Clinical Sciences, University of Exeter Medical School, University of Exeter, Exeter EX4 4PS, UK; 7 School of Physiology, Pharmacy, and Neuroscience, University of Bristol, Bristol BS8 1TH, UK; 8 Division of Neurobiology, MRC Laboratory of Molecular Biology, Cambridge CB2 0QH, UK; 9 Centre for Biological Timing, Faculty of Biology, Medicine and Health, School of Medical Sciences, University of Manchester, Manchester M13 9PT, UK; 10 Department of Biomedical Research, Inselspital University Hospital Bern, Freiburgstrasse 18, 3010 Bern, Switzerland

**Keywords:** circadian, sleep, siesta, optogenetics, vasoactive intestinal polypeptide, wake maintenance, alertness, napping

## Abstract

Although the mammalian rest-activity cycle is controlled by a “master clock” in the suprachiasmatic nucleus (SCN) of the hypothalamus, it is unclear how firing of individual SCN neurons gates individual features of daily activity. Here, we demonstrate that a specific transcriptomically identified population of mouse VIP+ SCN neurons is active at the “wrong” time of day—nighttime—when most SCN neurons are silent. Using chemogenetic and optogenetic strategies, we show that these neurons and their cellular clocks are necessary and sufficient to gate and time nighttime sleep but have no effect upon daytime sleep. We propose that mouse nighttime sleep, analogous to the human siesta, is a “hard-wired” property gated by specific neurons of the master clock to favor subsequent alertness prior to dawn (a circadian “wake maintenance zone”). Thus, the SCN is not simply a 24-h metronome: specific populations sculpt critical features of the sleep-wake cycle.

## Highlights

• Specific VIP+ neurons of the SCN fire at night when most others are silent
• Activating or silencing these VIP+ neurons controls nighttime but not daytime sleep
• Circadian clockwork within these specific neurons times the daily “siesta”
• The activity of these neurons, in turn, programs subsequent end-of-day alertness

## Teaser

The “master” circadian clock generally indicates day and night by tonic daytime firing. A specific population of neurons, active when the rest of the SCN is silent, directs nighttime “siesta” sleep but not daytime sleep, thereby timing end-of-day alertness.

## Introduction

Almost all organisms have internal circadian clocks that consolidate sleep to night in diurnal organisms or day in nocturnal ones. Sleep duration and intensity are additionally regulated by a homeostatic mechanism dependent upon prior waking experience (Borbély, 1982). Within this two-process model, it remains unclear to what extent the circadian clock acts as a “metronome”—indicating day and night—versus acting as an “orchestral conductor”—specifically shaping features of the sleep-wake cycle. Most animal species and all human cultures consolidate the majority of sleep to either day or night. Many also exhibit an additional period of sleep—a “siesta”—during the second half of the wake phase, followed by a period of increased alertness—the “wake maintenance zone” (WMZ)—thought to depend on sleep pressure (Ehlen et al., 2015; Owens et al., 2010; Reichert et al., 2014). How the mammalian circadian oscillator shapes this complex pattern is unknown.

The molecular basis of circadian behavior is based on transcription-translation feedback loops that oscillate every 24 h. Molecular rhythms in peripheral cells are entrained by a central clock located in the suprachiasmatic nucleus (SCN) 20,000 neurons at the base of the hypothalamus (Brown and Azzi, 2013). Overall, SCN neurons are most electrically active during the day; in mice, this correlates with periods of quiescence and sleep (Belle et al., 2009; Colwell, 2011; Lee et al., 2009), and blocking SCN neuronal firing during the day with tetrodotoxin (TTX) results in increased locomotor activity (Houben et al., 2014). Within the SCN circadian clock, genes and neuronal networks cooperate to generate robust rhythms and then translate these cellular oscillations into daily rhythms of behavior that persist under constant conditions (An et al., 2011; Aton et al., 2005, 2006; Brancaccio et al., 2013; Freeman et al., 2013; Harmar et al., 2002; Maywood et al., 2006; O’Neill et al., 2008). Although all SCN neurons contain the same molecular clock, anatomically distinct regions of the SCN—a ventral “core” expressing vasoactive intestinal polypeptide (VIP) and a dorsal “shell” expressing arginine vasopressin (AVP) (Varadarajan et al., 2018)—display circadian gene expression in slightly different phases (Aton and Herzog, 2005), a pattern thought to be important for enabling clock plasticity encoding seasonality and day length (Azzi et al., 2014, 2017; Evans et al., 2013). It has been suggested that different oscillator populations within the SCN track dawn and dusk and drive distinct peaks of behavioral activity: the morning (M) and evening (E) oscillator model (Pittendrigh and Daan, 1976). Supporting this idea, in *Drosophila*, distinct groups of clock neurons display phases of electrical activity that anticipate dawn or dusk and promote M or E locomotor activity, respectively (Grima et al., 2004; Liang et al., 2016; Stoleru et al., 2004), while other populations of clock neurons regulate sleep timing (Guo et al., 2016; Parisky et al., 2008). So far, little behavioral evidence exists to support this idea in mammals, though the overall “firing window” of SCN neurons expands or contracts with changing day length (VanderLeest et al., 2007).

Here, we demonstrate that a specific population of mouse VIP+SCN neurons is active at the “wrong” time of day—nighttime when most SCN neurons are silent—and is necessary and sufficient to gate nighttime sleep but has no effect on daytime sleep. In turn, the molecular clock within these neurons determines the specific timing of this nighttime sleep (a rodent “siesta”) and subsequent alertness prior to dawn (a circadian WMZ). Thus, the SCN is not just a metronome for 24 h rhythms but micromanages critical features of the sleep-wake cycle.

## Results

### Identification of Night-Active SCN Neurons

**Figure 1:**
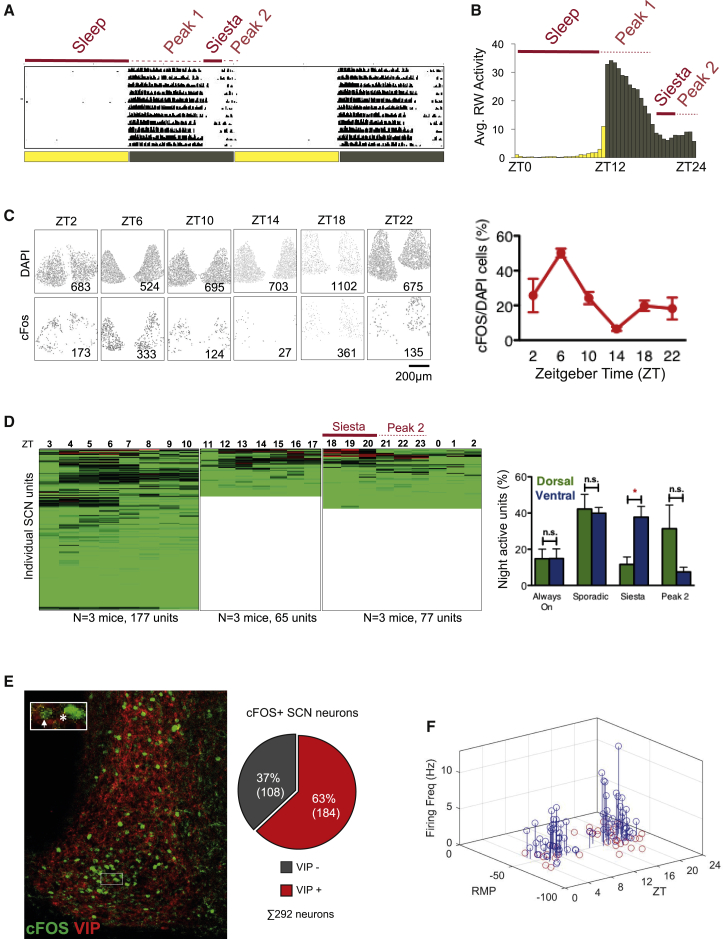
Identification of Night-Active SCN Neurons (A) Representative actogram of wild-type mouse RW activity in LD12:12. Note 2 peaks of RW activity separated by a period of quiescence and sleep, the siesta. (B) Average RW activity of 18 mice recorded for 7 days plotted in 30-min bins. Yellow bars indicate light; black bars indicate dark. (C) Left: representative immunostained SCN slices collected at indicated times (ZT, time relative to light onset). Top: DAPI (cell bodies). Bottom: c-FOS (marker of neuronal activity). Images were processed to remove background; only positive cells are shown. Inset: number of detected cells. Right: percentage of SCN DAPI+ cells co-expressing c-FOS (n = 3 animals per time point). (D) Left: unit activity detected across the day. Each row represents a unit active at any time point from one of 3 acute slices; totals are at bottom. Columns indicate time. Three trimmed, overlapping sets of measurements shown. Green, 0 Hz; Red, 3 Hz. Full data and quantification are in Figure S1. Right: Dorsal/ventral location and type of activity for each unit. Note that a higher proportion of ventral units show siesta-specific activity. (E) 40× maximum-projection images of SCN immunostained for c-FOS (green) and VIP (red) at ZT18. VIP+ axonal and dendritic staining is observed throughout the SCN, while VIP cell bodies are primarily ventral. Inset: arrow indicates neuron expressing VIP and c-FOS, Asterisk indicates neuron expressing only c-FOS. Pie chart: quantification of c-FOS and VIP cell body co-localization (red) versus c-FOS alone (gray); n = 6. (F) Patch clamp recordings of equal numbers of wild-type neurons recorded from nighttime and daytime slices. Blue, active neurons; red, inactive neurons. Axes indicate time, resting membrane potential, and firing rate. 1 h gap between daytime and nighttime units due to time of slice preparation. Further analysis is in Figure S2. Error bars represent SEM. ^∗^p < 0.05, 2-tailed Student’s t test. See also Figures S1 and S2.

Mouse running wheel (RW) behavior under 12:12 light:dark (LD) cycles includes 3 major time-of-day-dependent features: (1) a prolonged early-night activity peak starting just prior to dark onset at zeitgeber time (ZT)12, (2) a siesta (a period of quiescence and sleep) centered around ZT20, and (3) a second bout of activity anticipating lights on at ZT0 (Figures 1A and 1B). Although the SCN is most electrically active during the day (Colwell, 2011), for the SCN clock to control nighttime activity we hypothesized, some SCN neurons would be electrically active during one or more of these nighttime events. We therefore quantified SCN neuron electrical activity at six time points by immunostaining for the immediate-early protein c-FOS, a marker of neural activity (Schilling et al., 1991) (Figure 1C). Consistent with previous reports, significantly more neurons express c-FOS during the day than during the night (Colwell, 2011). However, populations of presumably active c-FOS-expressing neurons were identifiable at night in both dorsal and ventral regions of the SCN, albeit in lower numbers than during the day (Figure 1C). Interestingly, there appeared to be two peaks of firing, with the first (major) peak corresponding to the peak of daytime sleep and a second roughly corresponding to the peak of nighttime sleep (the siesta).

### Characterization of Night-Active SCN Neurons

To characterize night-active SCN neurons in more detail, we placed SCN slices on a multi-electrode array (MEA), allowing recording of unit activity over time (Figure S1A). We maintained individual SCN slices on the MEA for 9–10 h, recording for 10 min/h, and then identified and sorted spikes by waveform to identify active units—individual neurons or electrically similar groups of neurons—at each time point. Although only ∼16/59 electrodes contacted the SCN, 2/3 of all detected units came from within the SCN, with the main peak of SCN unit firing preceded firing from extra-SCN units by ∼2 h (Figure S1B). During the day (ZT0–12), we detected 177 units active at one or more time points (3 experiments), with the highest number of active units detected between ∼ZT5 and ZT8, during the daytime sleep period. At night (ZT16–24), we detected 77 active units across 3 experiments. Figure 1D shows these data as a proportionally compressed composite view, trimmed to eliminate overlapping recordings. (An expanded view of night-active neurons is shown in Figure S1C, representative recordings are in Figure S1D, and a composite sorting of all units is in Figure S1E.)

About 15% of night-active units (16/106; 4 experiments) were firing for the duration of the recording (“always on”), and ∼40% (40/106) were active for a brief period at one or more time points (“sporadic”). Both of these were equally distributed between the dorsal and ventral SCNs. Of the remainder, a further ∼20% (21/106) became active toward the end of the siesta and continued to fire to the end of the night (∼ZT21–24), coincident with the second RW activity peak; these “2nd peak” active units were predominantly dorsal SCN neurons, consistent with previous reports of AVP+SCN neuronal activity at this time (Gizowski et al., 2016). The remaining ∼25%, (25/106) were, by far, the most active, with a period of activity coincident with nighttime (siesta) sleep (∼ZT18–22): an expanded heatmap showing these units is in Figure S1C. These “siesta” active units were predominantly ventral (Figure 1D, right panel). As the ventral core region of the SCN is defined by VIP expression, we looked for co-expression of VIP and c-FOS at ZT18, around the beginning of the siesta (Figure 1E). Consistent with our previous results, roughly two thirds of c-FOS-positive neurons at ZT18 were VIPergic (Figure 1E). We next determined whether siesta-active neurons fired only during the siesta or were also active along with the majority of SCN neurons during the light period. Strikingly, most siesta-active neurons were siesta specific: of 27 siesta-active units, 21 were active only during the siesta (Figure S1F). Another 6 units showed an on-off-on pattern, beginning to fire again at the start of the next day, but even these fired more vigorously during the siesta (Figure S1G).

To further investigate night-active SCN neurons, we recorded electrical activity from randomly selected SCN neurons around the clock by patch clamp: Figure 1F shows all recorded neurons in a 3D graph, and Figure S2 shows individual neuron characteristics. As previously reported (Belle et al., 2009), we found SCN neurons that were firing (active cells) and two classes of inactive neurons: one hyperpolarized class with a resting membrane potential (RMP) ≤ −45 mV and a second depolarized population with a RMP ≥ −30 mV. (We injected current to repolarize each depolarized cell and verify that it could fire normal healthy action potentials.) Scoring for the time when neuronal activity was recorded, two peaks of firing were again observed: one during the day and one during the night, with equal RMP and firing frequency (Figures 1F and S2A–S2C). The majority of night-active neurons were visible during the siesta period (Figure S2D).

### Categorization of Night-Active SCN Neurons

**Figure 2:**
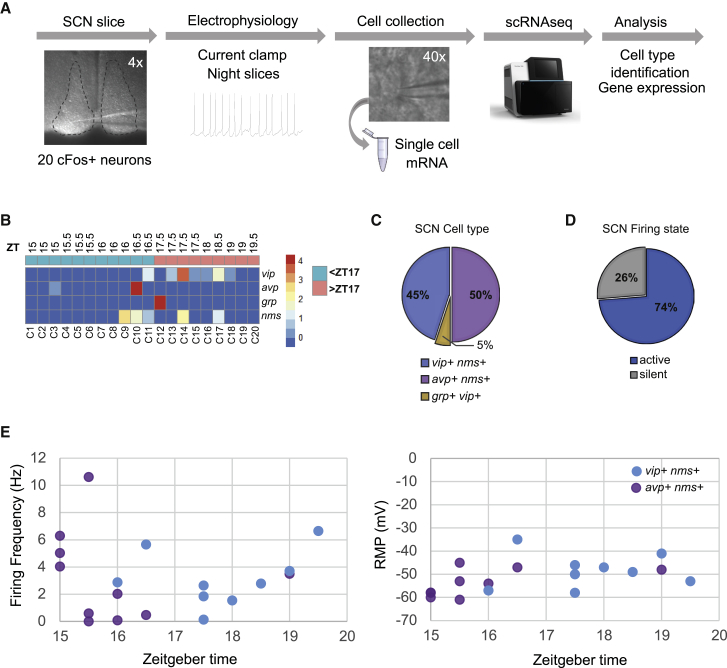
Characterization of Night-Active SCN Neurons by Patch-RNA-Seq (A) Ventral c-FOS::GFP+ neurons were recorded from ZT15–20 by patch clamp, then cytoplasm was collected for scRNA-seq (n = 20 neurons). (B) Heatmap showing expression of *vip*, *avp*, *grp*, and *nms* in neurons firing before and after ZT17. Color code shows *Z*-score normalized reads. Neurons firing after ZT17 express more *vip* compared to neurons firing before ZT17. (C) Pie chart of identified SCN neuronal classes, as defined by projecting each transcriptome to the SCN Drop-seq atlas (Wen et al., 2020). (D) Proportion of active (firing rate, >1 Hz) and silent neurons. (E) Electrophysiological properties of neurons from the two major classes: *avp*+ *nms*+ and *vip*+ *nms*+. Plots show firing frequency (in Hz; left) and resting membrane potential (RMP, in mV; right) over time. *avp*+ *nms*+ c-FOS::GFP+ neurons are mostly active in the early night (<ZT17) and *vip*+ *nms*+ c-FOS::GFP+ neurons from ~ZT17.

To better characterize night-active SCN neurons, we used c-FOS::GFP transgenic mice (Reijmers et al., 2007), where sustained neuronal activity induces GFP expression for a ∼3 h window. We took SCN slices from c-FOS::GFP mice to be ready to examine from ZT15 (∼2 h prior to siesta onset), identified GFP+ neurons throughout the siesta, recorded electrical activity in these different neurons by patch clamp, and then collected the cell contents of these neurons and characterized their transcriptomes by single-cell RNA sequencing (scRNA-seq; Figure 2A). First, we examined neuropeptide expression relative to time of collection of each active neuron. As predicted, VIP is predominantly expressed in siesta-active neurons compared to pre-siesta-active neurons (Figure 2B). Since one limitation of scRNA-seq is that all expressed genes are not detected in each library, we characterized each neuron by matching transcriptomes against the recently published scRNA-seq atlas of SCN neurons (Wen et al., 2020) to classify their type (prediction scores are shown in Table S1; see STAR Methods). From 20 ventral c-FOS::GFP+ SCN neurons, we identified two classes of night-active neurons within the SCN: a *vip*+ *nms*+ population (n = 9) and an *avp*+ *nms*+ population (n = 10), as well as a single *vip*+ *grp*+ neuron (Figure 2C). As expected, ∼75% of c-FOS::GFP+ neurons were electrically active at the time of collection (Figures 2D and 2E; c-FOS::GFP expression in non-active neurons likely represents recent prior electrical activity; see Table S2 for electrophysiological properties). Interestingly, *avp*+ *nms*+ SCN neurons were all collected in the early night (∼ZT15–16, during the first peak of RW activity), while *vip*+ *nms*+ neurons were collected toward the end of the night (∼ZT17–20, approximately the time of the siesta). These data suggest strongly that nighttime-active *vip*+ *nms*+ neurons are firing during the siesta, while *avp*+ *nms*+ nighttime-active neuronal activity precedes it.

### VIP+ SCN Neurons Are Necessary for Normal Nighttime Sleep Patterns

**Figure 3:**
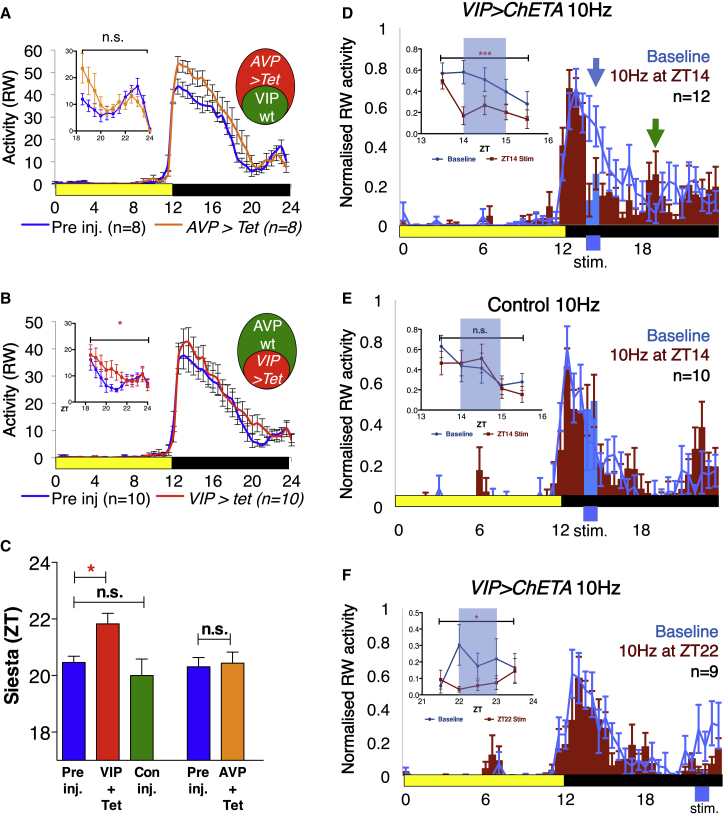
Neuronal Activity in VIP+SCN Neurons Regulates the Daily Siesta (A–C) RW activity of *Vip-CRE* and *Avp-CRE* mice injected at the SCN with AAV.Flex.TetLC. An average of 7 days of RW activity in 30 min bins is plotted for each genotype pre-injection (blue line), compared to 7 days of RW activity ≥2 weeks post-injection (green, orange, and red lines) when the virus should be fully expressed. Inset: ZT18.5–23.5 RW activity, 2-way ANOVA. (A) No effect of injection of TetLC virus into AVP-CRE mice on siesta activity, F(1, 168) = 2.776, n.s. (B) Injection of TetLC virus into VIP-CRE mice significantly increases RW activity during the siesta, F(1, 216) = 6.5, p < 0.05. (C) Quantification of the effect of VIP+SCN neurons upon siesta timing, defined as the point of lowest activity during the siesta. Comparisons by ANOVA with Tukey’s multiple comparison test for *VIP>Tet*: F(2, 35) = 5.892, p = 0.0062, otherwise Student’s t test. (D–F) RW activity of VIP-CRE mice injected at the SCN with the optogenetic probe AAV.ChETA. (D) RW activity of *VIP>ChETA* over 24 h before (baseline; blue line) or on day of stimulation (at 473 nm, which drives neurons to fire, via an optic fiber implanted between SCN lobes; red and blue bars). Stimulation for 1 h at 10 Hz at ZT14 (blue bars and blue arrow). Green arrow indicates new peak of RW activity that appears ~4 h post-stimulation (red bars, quantified in Figure S3). Inset: statistical summary before, during, and after stimulation; two-way ANOVA, F(1, 106) = 12.15, p < 0.005. (E) No effect of simulation for 1 h at 10 Hz at ZT14 on control-injected mice; F(1, 80) = 0.28, n.s. (F) Stimulation of VIP+SCN neurons for 1 h at 10Hz at ZT22 inhibits RW activity; F(1, 75) = 4.95, p < 0.05. In all panels, yellow: black bars represent 12-h:12-h LD cycles. Statistical comparisons are as specified. Error bars represent SEM. ^∗^p < 0.05; ^∗∗^p < 0.01; ^∗∗∗^p < 0.005. See also Figures S4 and S5.

Given that *avp*+ and *vip*+ SCN neurons are active at different times of night (Figure 2E), we hypothesized that each population could regulate different aspects of nighttime behavior. To test this, we measured the contribution of each subset of SCN neurons to RW activity using three strains of mice bred into a homogeneous C57bl6 background: *Vip-CRE* to target VIPergic neurons (Taniguchi et al., 2011), *Avp-CRE* to target AVPergic neurons (Harris et al., 2014), and *Syt10-CRE* to target all SCN neurons (Husse et al., 2011). Consistent with Franken et al. (2001), >90% of mice displayed a robust nighttime siesta in the presence of a RW; only mice displaying a daily siesta were used for subsequent experiments. Mice were injected at the SCN with either control or an *AAV.Flex.TetLC* virus (Adeno-associated virus) encoding tetanus toxin light chain (TetLC) in reverse orientation between *loxP* sites (Foster et al., 2015). Using this intersectional approach, in the presence of CRE, *TetLC* is flipped into the correct orientation and transcribed, blocking synaptic transmission (Figure S4A). We then compared RW activity in 12-h:12-h LD conditions for each mouse in the week prior to injection to RW activity in the week starting ∼14 days post-injection, allowing TetLC to be fully expressed in VIP+SCN neurons (hereinafter abbreviated *VIP>*), AVP+SCN neurons (*AVP>*) or the entire SCN (*Syt10>*). Injection of *AAV.Flex.TetLC* into the SCN after mice have reached adulthood eliminates the possibility of developmental defects caused by silencing VIP neurons, although we cannot rule out unknown compensatory mechanisms occurring after silencing. Control-injected mice showed no differences in distribution of activity pre- and post-injection (Figures 3C and S4C). *AVP>TetLC* mice showed a significant increase in RW activity from ZT12–18 post-injection, suggesting that AVP neurons may contribute to daily activity in the early night; however, siesta behavior was unaffected (Figures 3A and 3C), consistent with *avp+ nms+* SCN neurons being active in the early night only (Figure 2E). By contrast, *VIP>TetLC* disrupted the daily siesta, consistent with the late night activity of *vip*+ *nms*+ SCN neurons (Figure 2E): the only significant effect of blocking VIP+SCN signaling was to increase RW activity from ZT18.5–22 (Figure 3B). Furthermore, the timing of the siesta, as determined by the time of lowest RW activity in each mouse at ZT18–23 was delayed by ∼2 h in *VIP>TetLC* mice compared to that in controls or *AVP>TetLC* mice (Figure 3C). An example *VIP>TetLC* actogram is shown in Figure S4B; individual mice where VIP+SCN activity is silenced no longer show a defined period of quiescence and, instead, display random bouts of activity and inactivity across the latter half of the night. It should be noted that, although TetLC blocks synaptic transmission (*VIP>TetLC* caused a significant decrease in electrical activity within VIP+SCN neurons; Figure S4E), it does not affect the release of neuropeptides stored in dense core vesicles. Thus, the neuropeptide VIP itself may still be released, and we observe no changes in circadian period in *VIP>TetLC* mice (data not shown), unlike in *vip*^−/−^ mutants (Aton et al., 2005).

Since we also identified a single *vip*+ *grp*+ night-active neuron (Figure 2B), we performed *AAV.Flex.TetLC* injections on *Grp-CRE* mice (Sun et al., 2017): silencing of GRP+SCN neurons alone was insufficient to disrupt siesta behavior (Figure S4D). Interestingly, blocking synaptic transmission from the entire SCN (*Syt>TetLC*) results in ∼50% of mice becoming arrhythmic, even under LD cycles ∼10–15 days post-injection (representative mouse is shown in Figure S4F; average activity is shown in Figure S4G). This is in contrast to a previous report where ablation of the SCN did not prevent mice entraining to LD cycles (Schwartz and Zimmerman, 1991), but it is consistent with another report where SCN ablation blocked daily rhythms of sleep under LD cycles (Easton et al., 2004) as well as one in which TTX silencing of SCN neurons provoked acute activity (Houben et al., 2014).

### Activating VIP+SCN Neurons Inhibits Nighttime Activity

To confirm that VIP+SCN neurons regulate nighttime behavior, we next tested the effect of activating VIP neurons on RW activity. We injected the SCN of *Vip-CRE* mice with an AAV virus encoding a CRE-dependent version of the optogenetic tool ChETA (Gunaydin et al., 2010) (*VIP>ChETA*), which causes a neuron to fire upon stimulation with blue light (473 nm). We first asked what happened when we activated VIP neurons at ZT14, when most SCN neurons are normally inactive and mice display the first peak in RW activity. Stimulation of VIP+SCN neurons was achieved through illumination with 10 ms pulses of light at 473 nm at a frequency of 10 Hz for 1 h; optogenetic activation was verified by optogenetically stimulating SCN slices *in vitro* (Figure S5C). Similar stimulation conditions were previously reported to be sufficient to entrain the clock in constant darkness, using a slightly different version of the optogenetic channel rhodopsin (Jones et al., 2015). Optogenetic activation of VIP+SCN neurons for 1 h at 10 Hz at ZT14 caused a rapid and significant reduction in RW activity compared to baseline for the duration of stimulation (Figure 3D, blue arrow and bar), followed by a significant increase in RW activity ∼4 h post-stimulation compared to baseline (green arrow in Figure 3D: see also Figure 7). By contrast, stimulation at a lower frequency of 5 Hz resulted in a non-significant decrease in activity (Figure S5A): we suggest that activating VIP+SCN neurons at 10 Hz (but not 5 Hz) mimics the high-frequency VIP neuron activation that rapidly entrains circadian locomotor rhythms (Mazuski et al., 2018). No inhibition of activity was observed in control mice stimulated at 10 Hz (Figure 3E). An analogous, significant cessation of activity was observed for the duration of stimulation at ZT22, during the second peak of RW activity (Figure 3F). By contrast, no effects of stimulation were observed at ZT2, when the mouse is already quiescent (Figure S5B). We conclude that activation of VIP+SCN neurons is sufficient to induce siesta-like quiescence in active mice.

### Activation of VIP+SCN Neurons Increases Nighttime but Not Daytime Sleep

**Figure 4:**
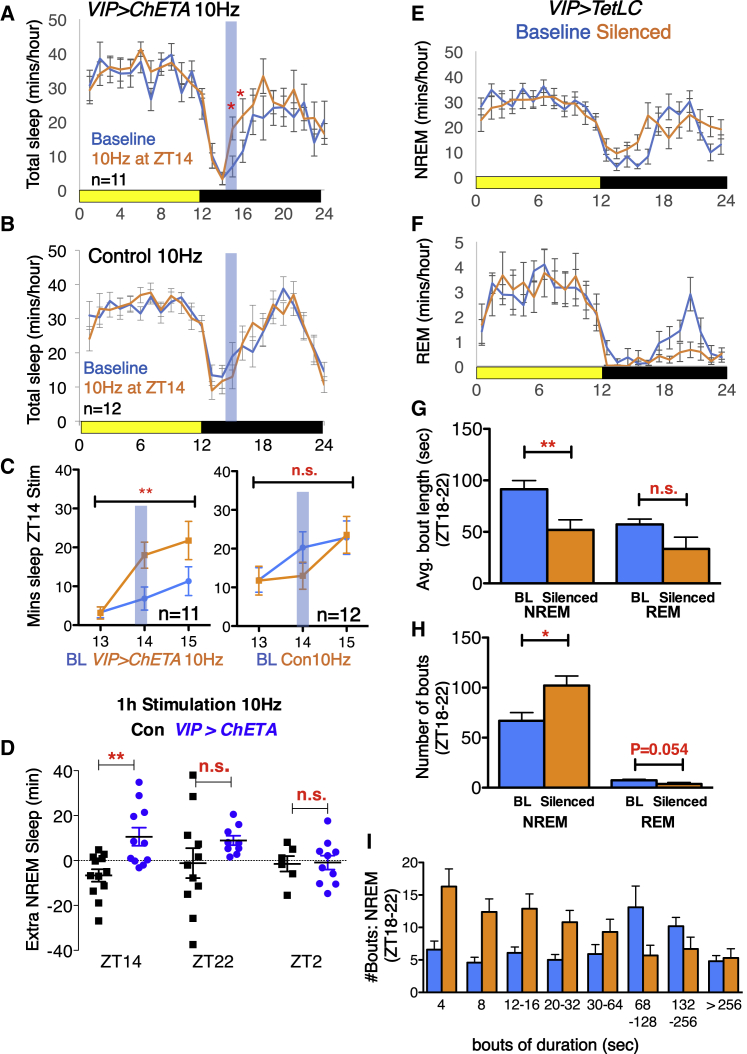
VIP+SCN Neuronal Activity Regulates Nighttime Sleep (A) Total sleep as minutes per hour in 1 h bins in *VIP>ChETA* mice on the day of stimulation of VIP+SCN neurons at ZT14 (orange line) compared to baseline (blue line). Stimulation for 1 h at 10 Hz is indicated at blue bar. (B) Same experiment, but with control-injected mice. (C) Statistical summary before, during, and after stimulation. Left: *VIP>ChETA*; two-way ANOVA, F(1, 60) = 7.373, p < 0.01. Right: control; F(1, 66) = 0.4899. (D) The change in NREM sleep (NREM during stimulation − baseline NREM) is plotted for ZT14, ZT22, or ZT2 for control (black) and *VIP>ChETA* (blue). NREM is significantly increased in *VIP>ChETA* mice at ZT14. All stimulations of *VIP>ChETA* mice at ZT22 increased NREM sleep, with activation at 10 Hz at ZT22 reducing variability between mice. F test to compare variance: NREM sleep, F(10, 8) = 12.26, p < 0.01. There was no effect of VIP+SCN neuron activation on sleep at ZT2. (E–I) Amount of NREM (E) and REM (F) sleep in *VIP>TetLC* mice at baseline prior to injection and post-silencing, in 1-h bins. Blue, baseline (BL), prior to silencing; orange, silenced. REM sleep is specifically reduced during the siesta; quantified in Figure S6. (G) Length of NREM and REM sleep bouts in *VIP>TetLC* mice at baseline and post-silencing. (H) Number of NREM and REM sleep bouts for same as in (G). (I) Distribution of bout lengths for same. Error bars represent SEM. Statistical comparison by two-tailed Student’s t test unless otherwise stated. ^∗^p < 0.05; ^∗∗^p < 0.01. See also Figures S6 and S7.

We next tested whether optogenetic activation of VIP+SCN neurons acutely affected sleep, using chronically implanted sleep electrodes to record electroencephalogram (EEG) and electromyogram (EMG) activity alongside RW activity; sleep data shown in Figure 4 are from the same cohort of mice as that providing RW data in Figure 3. During the first peak of RW activity (encompassing ZT14), mice normally show their lowest daily levels of sleep, and many mice do not sleep at all. With 10 Hz stimulation of *VIP>ChETA* SCN neurons at ZT14, total sleep (minutes of rapid eye movement [REM] + non-rapid eye movement [NREM]) during this interval was significantly increased, as were individual levels of NREM and REM sleep (Figures 4A, 4C, and 4D; Figures S6D, S6H, and S6I). No increase in sleep was detected during identical stimulations of control-injected animals (Figures 4B, 4C, and S6D) or in the same *VIP>ChETA* mice stimulated at 5Hz (Figures S6A and S6E) or in *VIP>ChETA* mice stimulated at 10 Hz at different times of day (2nd peak of activity/ZT22, quiescence/ZT2: Figures 4A, 4C, and 4D; Figures S6B–S6D and S6F–S6I). Overall, a highly significant cumulative increase in NREM but not REM sleep was seen when mice were stimulated at ZT14 during the first RW activity peak, a trend toward increase was seen at ZT22 during the second RW activity peak, and no increase was seen during quiescence at ZT2 (Figure 4D; Figure S6I). As mouse sleep is polyphasic, even at ZT2 mice are not asleep 100% of the time. We therefore conclude that signals from VIP+SCN neurons promote sleep either only at specific times of day or only when mice are normally most active.

### Silencing VIP+SCN Neurons Suppresses Nighttime but Not Daytime Sleep

Given that activation of VIP+SCN neurons both inhibits RW activity and promotes sleep, we tested whether *VIP>TetLC* mice show disrupted siesta sleep. We repeated the experiment described in Figure 3, injecting *Vip-CRE* mice with *AAV.Flex.TetLC* with sleep electrodes now implanted simultaneously with injection. We found a corresponding increase in the number of short NREM sleep bouts (<60 s) and reduction in the number of long NREM sleep bouts (>60 s) during the siesta after *VIP>TetLC*-induced silencing (ZT18–22; Figures 4E and 4G–4I). There was no effect on daytime sleep. Thus, blocking synaptic transmission from VIP+SCN neurons specifically increases siesta sleep fragmentation. Corresponding to this fragmentation, there was a dramatic reduction in REM sleep around the siesta (Figures 4F–4H, S7A, and S7B). It is likely that the reduction in the number of long NREM bouts decreases the likelihood that mice enter REM sleep, resulting in the reduction in levels of REM sleep observed. Consistent with this, the average number of REM sleep bouts during the siesta after VIP+SCN neuronal silencing is reduced, with some mice failing to enter REM sleep during this period (Figure 4H; Figure S7C). The effect of *VIP>TetLC* on REM sleep fits with previous reports of disruption of REM sleep in *vip*^−/−^ knockout mice (Husse et al., 2011); however, VIP signaling itself likely remains intact in the presence of TetLC (described earlier).

### *VIP+SCN* Neuronal Activity Gates Nighttime Sleep

**Figure 5:**
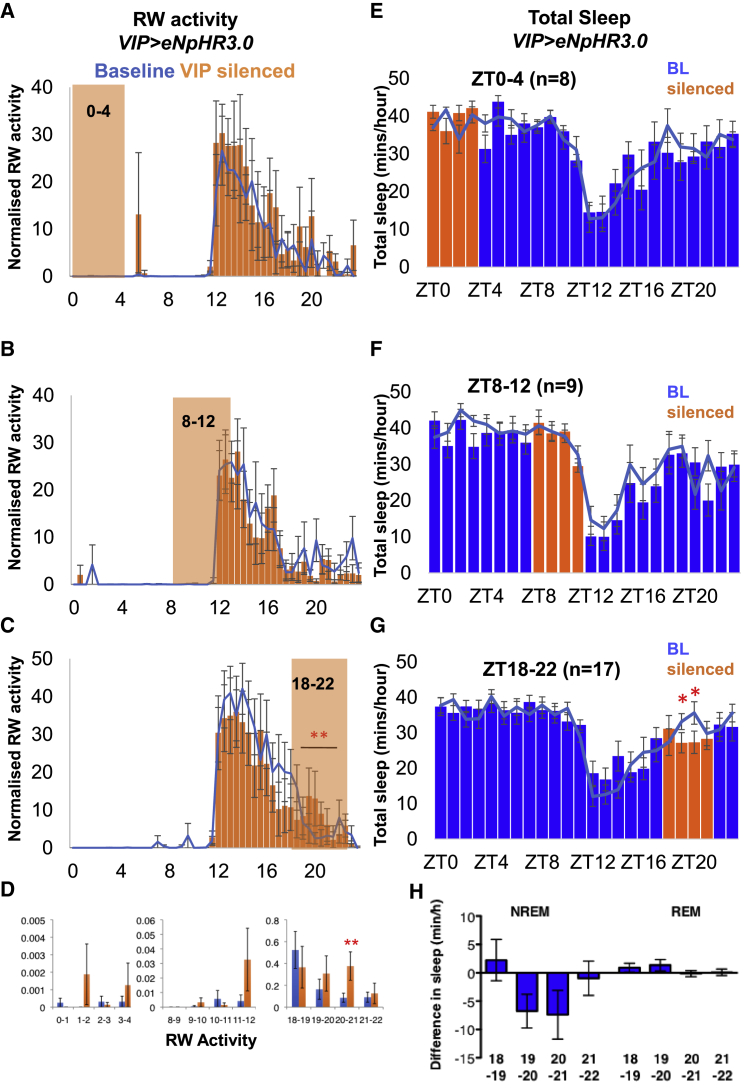
VIP+SCN Activity Regulates RW Activity and Sleep Only at Night (A–D) Normalized RW activity in 30 min bins in *VIP>eNpHR3.0* mice on the day of silencing of VIP+SCN neurons (orange bars) compared to baseline (blue line). VIP+SCN neurons were optogenetically silenced for 4 h in 2 min cycles of 1 min on, 1 min off (orange box). Silencing from (A) ZT0–4 (n = 5), (B) ZT8–12 (n = 8), and (C) ZT18–22 (n = 8). (D) Statistical summary showing a significant effect on RW activity only during silencing from ZT18–22. (E–H) Total sleep in 1 h bins in *VIP>eNpHR3.0* mice on the day of silencing of VIP+SCN neurons for 4 h in 2 min on/off cycles (blue bars; 4 h silencing is indicated with orange bars) compared to baseline (blue line). (E) ZT0–4. (F) ZT8-12. (G) ZT18–22. (H) The difference in NREM and REM sleep for each individual *VIP>eNpHR3.0* mouse between baseline and during 4 h silencing is shown for ZT18–22. Error bars represent SEM. Statistical comparison by two-tailed Student’s t test. ^∗^p < 0.05; ^∗∗^p < 0.01.

*VIP>TetLC* constitutively blocks the activity of VIP+SCN neurons but only affects nighttime sleep. This suggests that the effects of VIP+SCN neurons are gated to nighttime (or siesta) sleep. To test this, we injected *Vip-CRE* mice with a CRE-dependent virus encoding the optogenetic tool eNpHR3.0, which blocks neuronal firing in response to light delivered at 532 nm. VIP+SCN neurons were then silenced in 4 h windows from ZT0–4, ZT8–12, and ZT18–22 (Figure 5). Blocking activity of VIP+SCN neurons from ZT0–4, when mice are inactive and mostly asleep, or ZT8–12 when mice are transitioning from quiescence to anticipate the onset of night, had no effect on RW activity (Figures 5A, 5B, and 5D) or sleep (Figures 5E and 5F). In contrast, optogenetic silencing of VIP+SCN neurons during the siesta (ZT18–22) caused a significant increase in RW activity (Figures 5C and 5D) and concordant decrease in siesta sleep (Figures 5G and 5H). This confirms that the nighttime activity of VIP+SCN neurons is both required for and specific to the regulation of nighttime sleep. Thus, VIP+SCN neuronal signals provide a time-dependent gate on nighttime activity and sleep.

### The Molecular Clock of VIP+SCN Neuron Times the Daily Siesta

**Figure 6:**
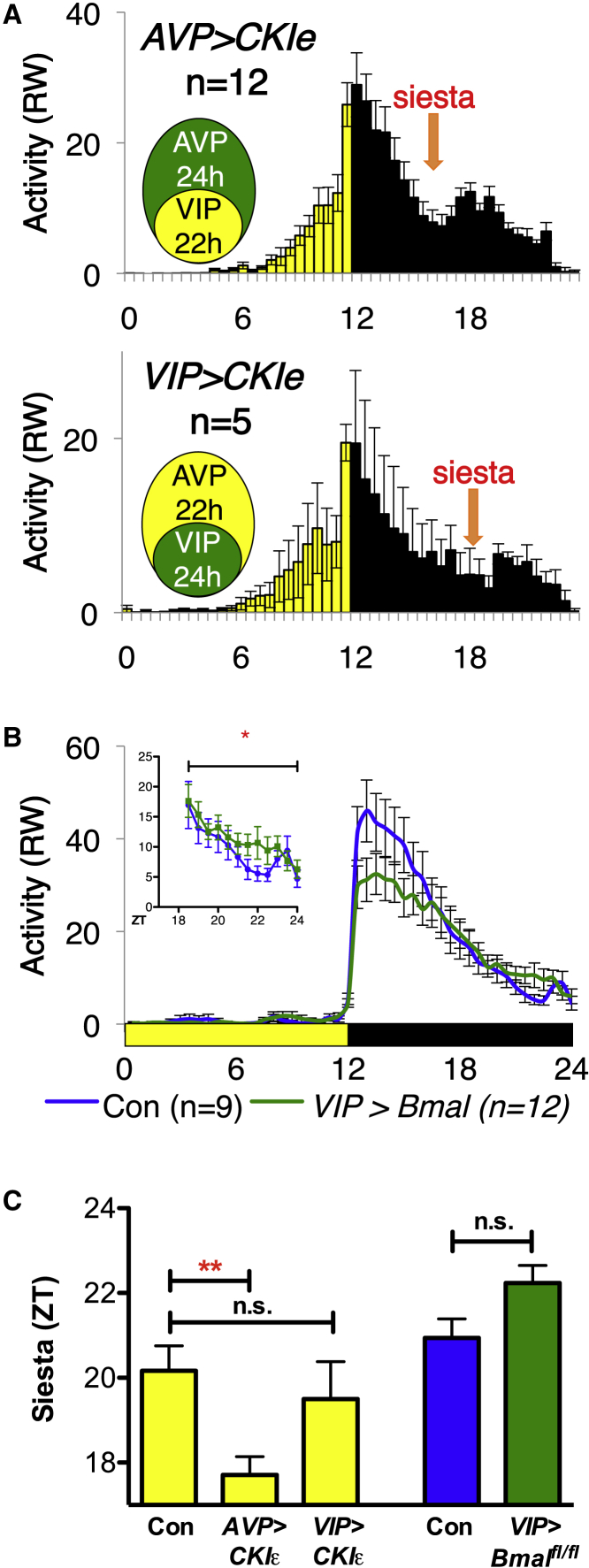
The Timing of the Siesta Is Regulated by the Molecular Clock RW activity was plotted in 30-min bins. Yellow:black bars represent 12 h:12 h LD cycles. Normalized RW activity is plotted in 30 min bins. (A) The *tau* allele of CKIe was used to shorten the period of VIP or AVP neurons. Top: *AVP>CKIe*^*tau*^ mice have a 22 h period in VIP neurons, 24 h in AVP neurons, and an advance in the timing of the siesta (orange arrow). Bottom: *VIP>CKIe*^*tau*^ ^*mice*^ with a 22 h period in AVP neurons but 24 h in VIP neurons have normal siesta timing (orange arrow). (B) *VIP > Bmal*^*fl/fl*^ mice lacking a functional clock in all VIP-CRE-expressing cells show increased RW activity during the siesta compared to sibling controls, F(1, 228) = 5.052, p < 0.05. (C) Quantification of the effect of VIP+SCN neurons upon siesta timing, defined as the point of lowest activity during the siesta. Comparisons by ANOVA with Tukey’s multiple comparison test for *CKIε*: F(2, 20) = 5.554, p = 0.0121 (*Bmal*^*fl/fl*^ by Student’s t test). Statistical comparisons as specified. Error bars represent SEM. ^∗^p < 0.05; ^∗∗^p < 0.01. See also Figure S3.

Time-dependent effects on behavior are often controlled by the circadian clock. Therefore, we tested whether the molecular clock within VIP+ neurons times the daily siesta using *CKIε*^*tau*^ mice (Meng et al., 2008) to change the period of either VIP or AVP neurons, measuring the effect on siesta timing. Heterozygous *CKIε*^*tau/+*^ mice have an ∼22-h period. CKIε^tau^ sits between flox sites and is deleted in the presence of CRE, resulting in a null allele; a single copy of wild-type CKIε is sufficient to generate a normal 24 h period. Thus, a mouse heterozygous for *CKIε*^*tau*^ crossed to a CRE line will result in a mouse where CRE-expressing cells have a 24 h period and CRE-negative cells have a ∼22 h period. Although both AVP-CRE and VIP-CRE are also expressed outside the SCN, RW rhythms depend on the SCN, so effects on RW activity are likely the result of SCN-specific manipulations. We generated *AVP>CKIε*^*tau/+*^ mice (24 h in AVP neurons/22 h in VIP SCN neurons) and *VIP>CKIε*^*tau/+*^ mice (22 h in AVP SCN neurons/24 h in VIP neurons). *AVP>CKIε*^*tau/+*^ mice in which VIP neurons have a short period have a siesta at ∼ZT17, significantly earlier than controls, whereas *VIP>CKIε*^*tau/+*^ mice, with a 24 h period in VIP neurons, have a siesta at ∼ZT20, similar to wild-type (Figures 6A and 6C). We conclude that the timing of the siesta is determined by the molecular clock within VIP+SCN neurons.

We also tested the effect of deleting a functional circadian clock in SCN neurons. BMAL1 is a core transcription factor at the heart of the molecular clock, and *Bmal1*^−/−^ mice are arrhythmic. Using a floxed allele of *Bmal1* (*Bmal1*^*fl/fl*^), *Bmal1* can be conditionally knocked out in specific groups of cells through expression of CRE recombinase (Storch et al., 2007). We generated mice carrying VIP-CRE and *Bmal1*^*fl/fl*^ (*VIP>Bmal*^*fl/fl*^), resulting in the deletion of *Bmal1* from all VIP-expressing cells. *VIP>Bmal1*^*fl/fl*^ mice show increased activity during the siesta (Figures 6B and 6C), similarly to *VIP>TetLC* mice (Figure 3B). Consistent with this, SCN siesta neuronal activity is reduced in *VIP>Bmal*^*fl/fl*^ mice compared to controls, as measured by MEA and c-FOS expression (Figure S4H). Thus, *VIP>Bmal1*^*fl/fl*^, like *VIP>TetLC*, reduces neurotransmission from VIP+SCN neurons, resulting in a disruption of the daily siesta. These data also suggest that the transcriptional state of the molecular SCN clock regulates neuronal activity. *VIP>Bmal1*^*fl/fl*^ mice also show reduced RW activity at the beginning of the night, a phenotype not observed in *VIP>TetLC* mice. Therefore, loss of a functional clock in VIP+ neurons likely affects pathways beyond those blocked by TetLC expression that are important at other times of day. Although we cannot rule out potential developmental defects caused by the constitutive removal of *Bmal1* from VIP+ neurons or expression of *CKIε*^*tau*^ within VIP+ or AVP+ neurons, this is not something that has been reported in any previous studies.

### Nighttime VIP+ Neuronal Firing Creates a Subsequent Circadian WMZ

We have demonstrated a clear causal relationship between firing of nighttime-active VIP+ neurons and a circadian clock-gated increase in siesta sleep. However, the physiological rationale for a clock-controlled siesta remains unclear. One function of sleep is to boost subsequent wakefulness, so we hypothesized that a pre-programmed siesta controlled by VIP+SCN neurons could increase alertness immediately afterward, at the end of the activity period. This end-of-activity wakefulness has been called the WMZ and has been extensively studied in humans (Zeeuw et al., 2018). To test the hypothesis that VIP+-driven nighttime sleep affects the WMZ, we re-examined our experiments to analyze sleep patterns in the hours following normal and (opto)genetically manipulated siesta sleep.

**Figure 7:**
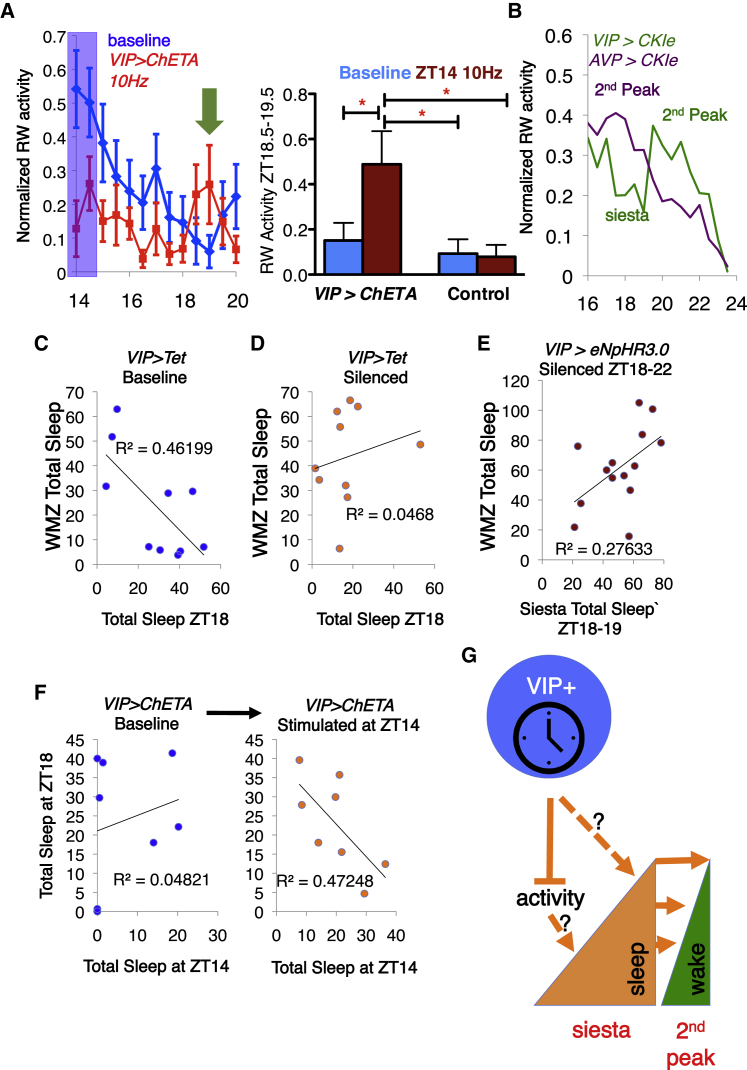
VIP+SCN Neuron-Driven Siesta Sleep Promotes Wake in the WMZ (A) Left: average RW activity of baseline (blue) and *VIP>ChETA* (red) is re-plotted from Figure 3D to show increased RW activity in mice ~4–5 h post-stimulation (green arrow). Blue box represents 1-h stimulation. Right: statistical analysis of same; RW activity is significantly increased from ZT18.5–19.5 in *VIP>ChETA* mice after induction of a “siesta” through stimulation at 10 Hz at ZT14, compared to baseline or control mice after same stimulation (one-way ANOVA). (B) RW activity of *AVP>CK1e* (purple) and *VIP>CKIe* (green) mice from Figure 6 re-plotted as normalized data to show the shift in timing of the second peak in *AVP>CKIe* mice. (C and D) Correlation between total amount of sleep in *VIP>Tet* mice at ZT18 and during the WMZ (ZT23–24), at baseline (C) and post-silencing (D). Note significant negative correlation at baseline, F(1, 8) = 6.87, p < 0.05; which is lost after silencing VIP+SCN neurons, F(1, 8) = 0.3928, n.s. Data are from Figure 3. (E) There is no significant correlation between siesta and WMZ sleep in *VIP>eNpHR3.0* mice upon optogenetic silencing from ZT18–22, F(1, 14) = 4.58, n.s. Data are re-plotted from 14/18 mice in Figure 4 that showed reduced siesta sleep from ZT19–20 during silencing. (F) Correlation between total amount of sleep in *VIP>ChETA* mice at ZT14 at baseline (left) or during 10 Hz stimulation (right) with sleep at ZT18. Note negative correlation between total sleep at ZT14 and sleep at ZT18 post-stimulation (data are from Figure 5); F(1, 5) = 6.771, p < 0.05; no correlation without stimulation, F(1, 5) = 0.4305, n.s. (G) Model for the generation of siesta sleep and promotion of wakefulness in the wake maintenance zone (WMZ). VIP+SCN neurons signal at night to inhibit locomotor activity and/or directly promote sleep. As sleep accumulates during the siesta, consolidated by signals from VIP neurons, the amount of sleep required during the 2nd peak of RW activity (WMZ) is reduced. Thus, we propose that VIP+SCN neurons promote activity during the WMZ by increasing sleep during the siesta. Statistical comparisons are as specified. Error bars represent SEM. ^∗^p < 0.05.
(2) Moving nighttime sleep earlier genetically via *CKIε*^*tau*^ (Figure 6A) also advances end-of-night activity (Figure 7B).
(3) More generally, higher levels of siesta sleep are associated with reduced sleep during the WMZ (Figure 7C), with silencing of VIP+SCN neurons disrupting this equilibrium (Figures 7D and 7E). Analogously, optogenetically stimulating sleep at ZT14 provokes a corresponding reduction in sleep at ZT18, effectively a new, artificial “circadian WMZ” (Figure 7F). A model illustrating the role of VIP neurons in driving the daily siesta and subsequent WMZ is shown in Figure 7G.

(1) Activation of VIP+SCN neurons for 1 h at ZT14 (but not ZT2 or ZT22) causes an immediate cessation of activity and increase in NREM and REM sleep (Figures 3D, 4A, 4D, S6D, S6H, and S6I), followed by a burst of RW activity from ∼ZT18.5–19.5 that resembles the peak of activity that normally marks the end of the siesta (Figure 7A), effectively moving WMZ-like behavior earlier to follow the premature siesta.

## Discussion

We show that a nighttime-active population of VIP+ neurons “sculpts” sleep and activity in gated fashion—i.e., the same neurons driven or silenced at other times of day do not exert these effects; moreover, a clock in these neurons determines the timing of their principal effects. This is the first report of an acute effect of mammalian SCN neurons upon sleep, a starting point in understanding how circadian clocks control what is arguably their major behavioral output.

Previous data suggest that the SCN is primarily active during the day in both nocturnal and diurnal mammals (Challet, 2007). However, these results do not preclude nighttime activity of some SCN neurons. Although studies of calcium signaling from VIP+ neurons have not specifically reported nighttime activity (Jones et al., 2018; Mei et al., 2018), several previous studies identify night-active SCN neurons using c-FOS labeling (Yan and Silver, 2008), electrophysiology (Belle et al., 2009), and membrane voltage indicators (Enoki et al., 2017). Here, we identify ∼20% of all SCN neurons as “night active” by c-FOS staining; our single-cell sequencing suggests that these night-active SCN neurons are either *avp*+ *nms*+, and active during the early night (to ∼ZT16) or *vip*+ *nms*+ and active during the daily siesta toward the end of the night. *Nms*+ neurons represent most SCN neurons and are essential for rhythmicity (Lee et al., 2015). By specifically targeting our optogenetic/chemogenetic experiments to VIP+ or AVP+SCN neurons, we were able to determine that the VIPergic, night-active *nms*+ population of SCN neurons is both necessary and sufficient to drive and time activity and sleep around the nighttime siesta, consistent with their phase of electrical activity. Using multiple bioinformatics tools, we have profiled gene ontologies unique to *vip*+ *nms*+ neurons (Figure S3). Beyond expression of *vip* itself, these tools depict changes in gene expression within the G-protein coupled receptor (GPCR) and adenyl cyclase signaling pathways, consistent with previously reported ERK1/2 signaling in response to VIP within the SCN (Hamnett et al., 2019). Also overrepresented are genes involved in the cellular response to light, suggesting that night-active *vip*+ *nms*+ neurons generally resemble neurons activated by light, even if no light stimulus occurred. Finally, upregulated genes within these pathways are also in common with a mouse circadian hyperactivity disorder model (Hagihara et al., 2016).

In normal LD cycles, VIP+SCN neurons have been shown to be important for changing circadian time (phase shifting) (Jones et al., 2018) and participate in neuropeptide-mediated coupling of SCN subregions (Maywood et al., 2011) (Hamnett et al., 2019). Because of their acute effects in silencing mouse activity, they have also been postulated to be essential to “masking,” the acute inhibition of rodent activity by light (Mazuski et al., 2018). Deletion of the gene encoding VIP silences SCN electrical activity (Brown et al., 2007), while high-frequency firing of VIP+SCN neurons is required for setting clock phase and behavior in a VIP-dependent manner (Mazuski et al., 2018). In circadian daytime, VIP neuronal signaling provides GABAergic inhibition to neighboring hypothalamic and thalamic regions to regulate circadian heart rate and corticosterone (Paul et al., 2020). Thus, a robust literature links VIP neurons to circadian function.

We suggest that these neurons play an even wider role, essentially sculpting nighttime behavior by determining “siesta napping” and the timing of subsequent wakefulness. Our data suggest two mechanisms by which VIP+SCN neuron activation could promote siesta sleep. First, VIP+SCN neurons may inhibit activity and promote quiescence, allowing mice to fall asleep more easily in response to sleep pressure, similarly to how humans lie down in bed before going to sleep. Alternatively, it is possible that VIP+SCN signals directly promote sleep and/or encourage fatigue.

For either mechanism, we find that a strict circadian gating occurs: firing or silencing these neurons has dramatic effects upon only nighttime activity and sleep. Even though mouse sleep is polyphasic, with periods of wake and sleep across the day and night, there is no discernible effect upon sleep of either activating or silencing VIP+SCN during the day. In this respect, the mechanism by which the SCN sculpts nighttime behavior is surprisingly similar to the *Drosophila* circadian clock circuit, in which a specific population of small ventral lateral neurons expressing the neuropeptide PDF are required for the M peak of activity anticipating dawn, while a subset of the remaining PDF− clock neurons (E cells) are required to anticipate dusk (Grima et al., 2004; Stoleru et al., 2004). In this scheme, VIP+SCN neurons might roughly resemble fly DN_1_ neurons that promote nighttime sleep by inhibiting activity-promoting E cells: these DN_1_s are also required for a normal daily siesta (Guo et al., 2016). Since the *Drosophila* siesta varies significantly between genders, it would also be interesting to examine gender differences in these proposed mechanisms in both mice and flies.

It has been suggested that a siesta occurs because of rising sleep pressure after prolonged periods of wake (Ehlen et al., 2015; Owens et al., 2010; Reichert et al., 2014). Even though a siesta is nonessential and genetically variable in mice (Franken et al., 2001), *Drosophila* (Ehlen et al., 2013)), and humans (Lopez-Minguez et al., 2017), our results suggest its specific utility: to maintain alertness at the end of the activity period. Across species, a period of increased activity anticipating dark:light transitions could be useful both metabolically as a last opportunity to forage before sleeping and to avoid risk of crepuscular predation. A programmed daily siesta could also aid entrainment to changing seasonal conditions by providing a mechanism by which an organism extends its active period; indeed, in rats, napping is highly dependent upon photoperiod, with longer nights creating more pronounced naps (Franken et al., 1995). In humans, a better understanding of the mechanisms underlying our own well-documented mid-afternoon fatigue and early evening WMZ (Lack and Wright, 2007) could provide an important intervention point to improve sleep consolidation.

## STAR★Methods

### Key Resources Table

**Table:** 

REAGENT or RESOURCE	SOURCE	IDENTIFIER
**Antibodies**		

mouse anti-c-Fos	Santa Cruz Biotechnologies	Cat#sc-166940; RRID: AB_10609634
rabbit anti-VIP	Peninsula Laboratories	Cat#T-4246.0050; RRID: AB_518682
chicken anti-GFP	Aves Labs	RRID: AB_2307313

**Bacterial and Virus Strains**		

AAV-EF1a-DIO-ChETA-EYFP	UNC Vector Core (Gunaydin et al., 2010)	N/A
AAV-EF1a-DIO-YFP	UNC Vector Core (Gunaydin et al., 2010)	N/A
AAV-EF1a-DIO-eNpHR3.0-EYFP	UNC Vector Core Gradinaru et al., 2010	N/A
AAV.Flex.TetLC	UZH Vector Core; Foster et al., 2015	N/A

**Critical Commercial Assays**		

SMART-Seq HT kit	Takara Bio	Cat#634438
Nextera XT DNA Sample Preparation Kit	Illumina	Cat#FC-131-1096
NextSeq 300 high-output kit	Illumina	Cat#20024908

**Deposited Data**		

Raw and analyzed data	This paper	Zenodo: 10.5281/zenodo.3946217
scRNAseq data	This paper	GEO: GSE154038

**Experimental Models: Organisms/Strains**		

VIP-CRE	Taniguchi et al., 2011	https://www.jax.org/strain/031628
AVP-CRE	Harris et al., 2014	https://www.jax.org/strain/023530
GRP-CRE	Sun et al., 2017	N/A
Syt10-CRE	Husse et al., 2011	N/A
*CKIe*^*tau*^	Hastings lab; Meng et al., 2008	N/A
*Bmal1*^*fl/fl*^	Storch et al., 2007	N/A
cFOS::GFP		https://www.jax.org/strain/018306

**Software and Algorithms**		

Trimmomatic	Bolger et al., 2014	http://www.usadellab.org/cms/index.php?page=trimmomatic; RRID: SCR_011848
Flexbar	Dodt et al., 2012	https://github.com/seqan/flexbar; RRID: SCR_013001
HTSeq	Anders et al., 2015	https://htseq.readthedocs.io; RRID: SCR_005514
scran	Lun et al., 2016	http://bioconductor.org/packages/release/bioc/html/scran.html; RRID: SCR_016944
samtools	Li et al., 2009	http://samtools.sourceforge.net/; RRID: SCR_002105
zinbwave	Risso et al., 2018	https://bioconductor.org/packages/release/bioc/html/zinbwave.html
DESeq2	Love et al., 2014	https://bioconductor.org/packages/release/bioc/html/DESeq2.html; RRID:SCR_015687
Seurat	Butler et al., 2018; Stuart et al., 2019	https://satijalab.org/seurat/; RRID:SCR_016341
GSEA	Subramanian et al., 2005; Mootha et al., 2003	https://www.gsea-msigdb.org/gsea/index.jsp; RRID:SCR_003199
STAR Aligner	Dobin et al., 2013	https://github.com/alexdobin/STAR; RRID: SCR_015899
pCLAMP	Molecular Devices	https://www.moleculardevices.com/products/axon-patch-clamp-system/acquisition-and-analysis-software/pclamp-software-suite
Neuroexplorer	Neuroexplorer	https://www.neuroexplorer.com/; RRID:SCR_001818
Offline Sorter	Plexon	http://plexon.com/products/offline-sorter; RRID:SCR_000012
Fiji	Fiji	https://fiji.sc; RRID:SCR_002285
Perseus	Perseus	http://maxquant.net/perseus; RRID:SCR_015753

### Resource Availability

#### Lead Contact

Further information and requests for resources and reagents should be directed to and will be fulfilled by the Lead Contact, Steven Brown (steven.brown@pharma.uzh.ch).

#### Materials Availability

No new materials were generated in this work

#### Data and Code Availability

Data is available in the Zenodo repository (10.5281/zenodo.3946217) and scRNaseq data is in the GEO NCBI repository (GEO: GSE154038). No novel code was generated in this work.

### Experimental Model and Subject Details

#### Mouse strains and husbandry

The following transgenic mouse strains were used: *Syt10-CRE* (Husse et al., 2011), *Vip-CRE* (Taniguchi et al., 2011), *Avp-CRE* (Harris et al., 2014), *Bmal*^*fl*^ (Storch et al., 2007), *CKIε*^*tau*^ (Meng et al., 2008), *Grp-CRE* (Sun et al., 2017) and *cFos-GFP* mice (https://www.jax.org/strain/018306).

### Method Details

All procedures were performed on 6-8 week old male mice backcrossed into a BL6/J background, under 12:12LD in single cage housing with running wheel and food/water available *ad lib*. No mice had been subjected to any previous procedures prior to the start of each experiment. For sleep and running wheel experiments, behavior of mice was recorded continuously. Control mice are siblings from the same litters as experimental animals. All animal experiments were conducted in accordance with applicable veterinary law and approved by the Zürich cantonal veterinary office.

### Quantification and Statistical Analysis

Statistical analysis was performed by Student’s t test or ANOVA, as specified in figure legends; electrophysiology and patch seq analysis was performed as described below. All statistical analysis and sample sizes are standards for the field, and therefore no specific estimation was made as to whether data met assumptions of the statistical approach.

#### Immunocytochemistry and cFOS quantification

Mice were perfused with PBS/4% PFA at 4h intervals over 24h of a 12:12LD cycle. Brains were incubated for 90 min in PBS/4% PFA at RT then in 30% sucrose solution in PBS overnight at 4°C, followed by freezing. 40μm coronal slices including the SCN were cut on a Thermo Scientific Microm HM 560 Cryostat. Free-floating SCN slices were washed 6x10 minutes in 10XPBS; incubated 1h in blocking solution (0.1M PBS, 2.5% NGS, 0.3% Triton-X, 0.005% sodium azide) at RT; incubated for 48h at 4°C in blocking solution plus mouse anti-c-Fos (1:50; Santa Cruz Biotechnologies), rabbit anti-VIP (1:600; Peninsula Laboratories) and/or chicken anti-GFP (1:1000); washed 6x in 10xPBS; incubated with 2° antibodies for 1h at RT in the dark; 6x10 min washes in 10xPBS with DAPI added to the last wash (1:1000). Slices were mounted using Vectashield (Vector Laboratories Inc.) then a Z stack was imaged on a Zeiss LSM 710 confocal microscope at 20x magnification. This image was converted into a Z-projection in *ImageJ.* The SCN was identified by DAPI staining, and a threshold was set to eliminate non-specific background outside the SCN. The option *Analyze Particles* was then used to quantify the number of cFOS positive cells within the SCN.

#### Patch clamp and multi-electrode recordings

##### Slice preparation

For patch clamp recordings, mice were sacrificed and brains were quickly removed. Brains were mounted on a Vibrating Microtome 7000 from Campden Instruments in a chamber filled with ice-cold and oxygenated artificial cerebrospinal fluid (ACSF) containing (in mM): 85 NaCl, 75 sucrose, 24 NaHCO_3_, 2.5 KCl, 1.25 NaH_2_PO_4_, 0.5 CaCl_2_, 4 MgCl_2_ and 25mM glucose saturated with 95% O_2_, 5% CO_2_ at pH 7.4. 300μm thick coronal slices containing the SCN were incubated for 30 min in ACSF before recording. For the MEA, mice were sacrificed and brains were quickly removed. Brains were cut with the vibratome in ice cold artificial cerebro-spinal fluid (ACSF in mM: NaCl 95; KCl 1.8; KH_2_PO_4_ 1.2; CaCl_2_ 0.5; MgSO_4_ 7; NaHCO_3_ 26; glucose 15; sucrose 50; oxygenated with 95% O_2_; 5% CO_2_; pH 7.4, measured osmolarity 310 mosmol kg-1). Slices were incubated for at least 1 hour in ACSF at room temperature and then transferred to the recording chamber, perfused continuously with ACSF at room temperature.

For patch-seq experiments, 300μm SCN coronal slices were incubated at 33°C in ice-cold sucrose ACSF (85 mM NaCl, 75 mM sucrose, 2.5 mM KCl, 25 mM glucose, 1.25 mM NaH2PO4, 4 mM MgCl2, 0.5 mM CaCl2 and 24 mM NaHCO3) for 30 min and then kept at room temperature until recording.

##### Whole cell recordings

Current clamp and voltage clamp recordings were performed using the HEKA EPC-10 amplifier and Patch Master software (HEKA Elektronik, Germany) at RT. Patch pipettes were pulled from borosilicate glass capillaries (DMZ Zeitz Puller), had a resistance of 5-7 MΩ and were filled with a K-gluconate intracellular solution (in mM: K-gluconate 115; KCl 20; Mg-ATP 2, Na_2_-ATP 2; Na_2_-phosphocreatine 10; GTP 0.3; HEPES 10; measured osmolarity 295-300 mosmol kg-1; pH 7.3-7.4). A GΩ seal was reached and the cell membrane was ruptured under voltage-clamp. Spontaneous activity of SCN neurons was measured in current-clamp mode. After measuring the resting membrane potential and the spontaneous firing frequency, the membrane potential was manipulated by current injection steps (increased steps of 10pA in 15 sweeps and decreased steps of 10pA in 10 sweeps). All the recordings were then analyzed with the software IgorPro and neurons with a difference of 20% in the test pulse were discarded.

For patch-seq experiments, patch pipettes were pulled from borosilicate glass pipettes with filament (Harvard Apparatus; GC150F-10; o.d., 1.5 mm; i.d., 0.86 mm; 10 cm length) and recordings were made with MultiClamp 700B Amplifier (Molecular Devices). Voltage-clamp and current-clamp recording were performed at 33°C with ACSF (126 mM NaCl, 2.5 mM KCl, 10 mM glucose, 1.25 mM NaH2PO4, 2 mM MgCl2, 2 mM CaCl2 and 26 mM NaHCO3). Only neurons with a series resistance lower than 25MΩ were selected for analysis. The firing frequency and resting membrane potential were analyzed with Clampfit 10.7.

##### Multi-electrode recordings

After 30 minutes incubation, a 300μm slice containing the SCN was placed on a 60pMEA100/30iR-Ti-gr perforated array (Multi Channel Systems). Slices were positioned so that the entire SCN was in contact with the electrode region of the array, and kept in place with a weight, with suction from underneath to maximize contact between the slice and array. Oxygenated ACSF at 34°C ran continuously through the MEA chamber for the duration of the experiment (1.2ml/min inflow/17ml/min outflow + gravity flow inflow/suction outflow at 65). Field potential detected by the MEA at 20,000Hz using *Multi-Channel Experimenter* (Multi Channel Systems). Because of the large file size, recordings were limited to 10 minutes at the start of each hour for the duration of the experiment. Data were analyzed using Offline Sorter (Plexon) as follows: files were run through a butterworth high pass filter at 300Hz and ‘spikes’ were detected using a threshold of ± 4 Standard Deviations. For each spike the waveform was analyzed and a unit assigned to each unique waveform detected from an individual electrode using the Valley Seeking spike sorting algorithm. Spikes were distinguished from noise by waveform. Data was analyzed using PRISM (GraphPad), and *Perseus* (http://maxquant.net/perseus/.) was used to hierarchically cluster individual unit activity across recordings.

#### Patch-Seq

##### Electrophysiology

See above.

##### Sample collection and processing

Methods and practices were as in (Földy et al., 2016). Briefly, a small amount of intracellular solution (< ∼1μl) in the patch pipette was used to record and collect neurons. Cell cytoplasm was aspirated into the patch pipette and immediately transferred into a microtube containing lysis buffer and RNAase inhibitor. The microtube was immediately frozen on dry ice and stored at −80°C until processing using Clontech’s SMART-Seq HT kit. cDNA was analyzed on the Fragment Analyzer (Advanced Analytical) before preparing the libraries with Nextera XT DNA Sample Preparation Kit (Illumina). Cells were pooled and sequenced using an Illumina NextSeq 500 system.

##### Bioinformatics

Raw reads were de-multiplexed and pre-processed using Trimmomatic and Flexbar then aligned to the Ensembl GRCm38 reference transcriptome (Version-2015-06-25), using the STAR aligner [trimLeft = 10, minTailQuality = 15, minAverageQuality = 20 and minReadLength = 30, ’Single-end/paired-end’ and ’sense/antisense/both’]. Gene counts were calculated using HTSeq. Ensembl gene IDs were converted to gene symbols using the mouse GRCm38 gtf file (ftp://ftp.ensembl.org/pub//release-86/gtf/mus_musculus/Mus_musculus.GRCm38.86.gtf.gz). In the few cases where different Ensembl gene IDs identified the same gene symbol, average gene counts were used.

For quality control, for each cell the total number of unique genes detected with at least 1 mapped read was calculated, and the number of mapped reads. We then calculated the median and median absolute deviation of these 2 values across all cells. Cells that were more than 3 median absolute deviations below the median in either category were rejected as poor quality. Cells that passed this quality control were then pooled together and normalized using scran (Lun et al., 2016), with sizes 40, 80, 120, 160, 200. Cells that had negative or zero size were removed.

To identify the neuronal subtype of each single neuron, Patch-seq data was projected to the SCN Drop-seq dataset from (Wen et al., 2020). The label transfer function from the R package Seurat was used. For each SCN neuron, a prediction score for each subtype is given. The cell type of a neuron corresponds to the subtype with the highest prediction score.

Differential gene expression analysis was performed using DESeq2 and EdgeR tools with the Zinb-Wave package as recommended for scRNaseq data, and the test = ”LRT” was used for significance. Gene ontology analysis was performed with GSEA tool (Mootha et al., 2003; Subramanian et al., 2005).

##### Locomotor activity recordings and analysis

Mice were individually housed in cages containing running wheels with *ad libitum* access to food and water. Mice were kept under 12:12LD cycles for the duration of experiments. Data was collected and analyzed using ClockLab (Actimetrics). For LD graphs, behavior was sorted into 30 min bins and the average behavior over 7 days for each mouse was used to generate activity plots, except in optogenetic experiments where a single day of data was used for baseline activity, paired with activity on the day of stimulation. For experiments where a sleep electrode was also in place, mice were placed in specialized sleep boxes allowing continuous recording of EMG, EEG and RW activity. Mice housed in sleep boxes showed increased variability in RW activity owing to differences in running wheel design, RW data from optogenetic stimulations was therefore normalized to allow comparisons between animals.

For siesta timing analysis, the time point of the siesta for each animal was calculated as the 30 min time bin with the lowest level of running wheel activity between ZT18 and ZT23 (as a proxy for the midpoint of the siesta). Where more than one bin had the same low level of activity, an average time was calculated.

#### Surgical Procedures

##### AAV Viral injections

To target the SCN, mice anaesthetized with isofluorane were injected with 750nl at 150nl/min of a 1:1 dilution of virus with PBS at ± 0.18mm x; −0.46mm y; −5.9mm z relative to bregma using a Kopf Stereotaxic instrument. In all experiments, mice were returned to the running wheel cage post injection, and allowed to recover while recording running wheel activity. The following Serotype 5 virus’ were injected: AAV.Flex.TetLC (Foster et al., 2015) (UZH Vector Core, 1x10^12^ viral particles/ml); AAV-EF1a-DIO-YFP and AAV-EF1a-DIO-ChETA-EYFP (Gunaydin et al., 2010), AAV-Ef1a-DIO-eNpHR3.0-EYFP (UNC Vector Core, 3.9x10^12^ viral particles/ml and 6x10^12^ viral particles/ml respectively). Post-experiment, sites of injection were histologically confirmed. Overall accuracy in targeting SCN was > 90%; unsuccessfully targeted animals were excluded from further analyses.

##### Optrode and sleep electrode implantation

Methods used here are based upon those in (Palchykova et al., 2010). For EEG recordings, mice were implanted epidurally with gold-plated screws (0.9mm diameter) under isoflurane inhalation anesthesia. Ceftriaxone was administered during surgery along with buprenorphine for analgesia, and enrofloxacin was provided in drinking water post surgery. Screws were placed in the right frontal (∼1.5mm anterior to bregma, 2mm lateral to the midline) and right parietal hemisphere (∼2mm posterior to bregma and 3mm lateral of the midline). Two gold wires (0.2mm diameter) were inserted bilaterally in the neck muscle for EMG recordings. Screws were connected to stainless steel wires and fixed to the skull with dental cement.

For optogenetic experiments, mice were injected with virus encoding the optogenetic tool and chronically implanted with an optrode between the 2 SCN lobes (0x; −0.46y; −6.0z from bregma) simultaneous with sleep electrode implantation.

##### Optogenetic stimulation and silencing

The optogenetic stimulation protocol was based upon the procedure previously shown to be sufficient to entrain the circadian clock (Jones et al., 2015). Experimental animals were VIP-CRE mice injected with AAV-EF1a-DIO-ChETA-EYFP; controls are sibling VIP-CRE mice injected with AAV-EF1a-DIO-YFP, or siblings of VIP-CRE mice that lack the VIP-CRE transgene, injected with AAV-EF1a-DIO-ChETA-EYFP. For optogenetic silencing, experimental animals were VIP-CRE mice injected with AAV-EF1a-DIO-eNpHR3.0-EYFP.

After recovery from surgery, a patch cord connecting a 473nm laser (activation) or 532nm (silencing) (LaserGlow Technologies) was attached to the ferrule covering the optical fiber. With the patch cord in place, the whole implant was covered and painted to prevent light leakage during stimulation. This was kept in place for the duration of the experiment (multiple stimulations at different times). For stimulation, 1h of 10ms pulses of light stimulation at 5Hz or 10Hz were driven by a Master-9 Pulse stimulator (A.M.P.I.), triggered at the appropriate time. For silencing, 532nm light stimulation was delivered in 2 minute cycles of 1 min on/1 min off for 4h, triggered by a Master-9 Pulse stimulator (A.M.P.I.), triggered at the appropriate time. Individual mice were stimulated every 4-5 days, enough time to recover from the previous stimulation.

In all optogenetic experiments, RW activity and sleep were recorded simultaneously. For analysis, RW activity and sleep on the day of stimulation was compared to baseline where mice, with patch cord attached, were not stimulated. Stimulation experiments ceased when mice damaged the opto-sleep implants, when repeated stimulations caused mice to stop running and/or become arrhythmic under LD conditions, or when mice reached ∼24 weeks of age.

##### Sleep recording and analysis

The EEG and EMG signals were amplified (amplification factor, ∼2000), filtered (high pass filter: –3 dB at 0.016 Hz; low pass filter: –3 dB at 40 Hz) sampled with 512Hz, digitally filtered [EEG: low pass finite impulse response (FIR) filter, 25 Hz; EMG: bandpass FIR filter, 20–50 Hz or 10-30Hz], and stored with a resolution of 128 Hz. Data analyses and statistics were performed in Excel, PRISM and the MATLAB software package (MathWorks). Sleep was scored by computer assisted staging (Miladinović et al., 2019) followed by visual inspection according to criteria in (Deboer et al., 1994; Franken et al., 1994).

## Supplemental Information

Document S1. Figures S1–S7 and Tables S1 and S2

Document S2. Article plus Supplemental Information
