# Supplementary material for: Circadian VIPergic Neurons of the Suprachiasmatic Nuclei Sculpt the Sleep-Wake Cycle
Source: Neuron. 2020 Nov 11;108(3):486–499.e5. doi: 10.1016/j.neuron.2020.08.001 (PMC7803671; doi:10.1016/j.neuron.2020.08.001)
Supplement: Document S1. Figures S1–S7 and Tables S1 and S2 [file mmc1.pdf]

Neuron, Volume *108*

Supplemental Information

Circadian VIPergic Neurons of the Suprachiasmatic Nuclei Sculpt the Sleep-Wake Cycle

Ben Collins, Sara Pierre-Ferrer, Christine Muheim, David Lukacsovich, Yuchen Cai, Andrea Spinnler, Carolina Gutierrez Herrera, Shao'Ang Wen, Jochen Winterer, Mino D.C. Belle, Hugh D. Piggins, Michael Hastings, Andrew Loudon, Jun Yan, Csaba Földy, Antoine Adamantidis, and Steven A. Brown

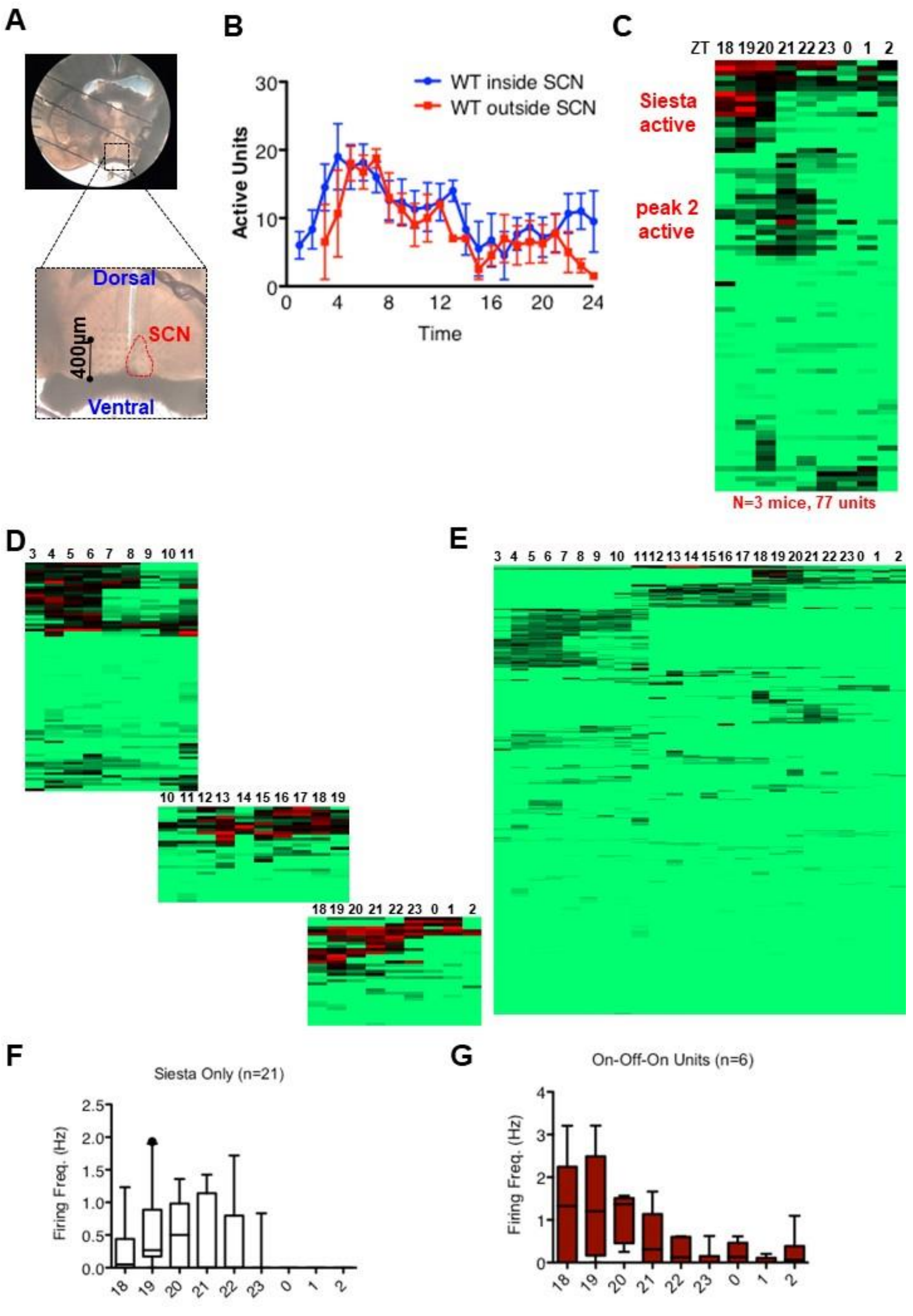

Figure S1: Further characterization of night active SCN neurons

Related to Fig 1

A) Representative image of a typical SCN-containing acute slice *in situ* on the multi-electrode array, with electrodes visible through the slice. **B)** The number of detectable units for a single slice on the MEA varies over time both inside (blue) or outside (red) the SCN. Note that if ~16 electrodes contact the SCN, ~44 are outside the SCN. Thus there are 3x as many active units/electrode within the SCN as outside the SCN. Error bars represent SEM **C)** SCN Electrical activity recorded from the MEA at ZT18-ZT2, plotted as unit activity over time. Note siesta units, some of which become active again at the beginning of the day (quantified in **Fig S1F,G**). Each row represents a unit active at any time point, columns indicate measurement time. Green=0Hz; Red=3Hz. **D)** Example of individual overlapping MEA recordings spanning 24h. Each panel represents a single experiment**. E)** Composite figure showing the timing of all units recorded from all experiments (n=10). **F-G)** Activity of siesta-active SCN units over time; **F)** Most siesta active units are active only during the siesta and are quiescent by ZT23; **G)** a minority of siesta active units are active during the siesta then turn off, then become active again at a much lower firing rate.

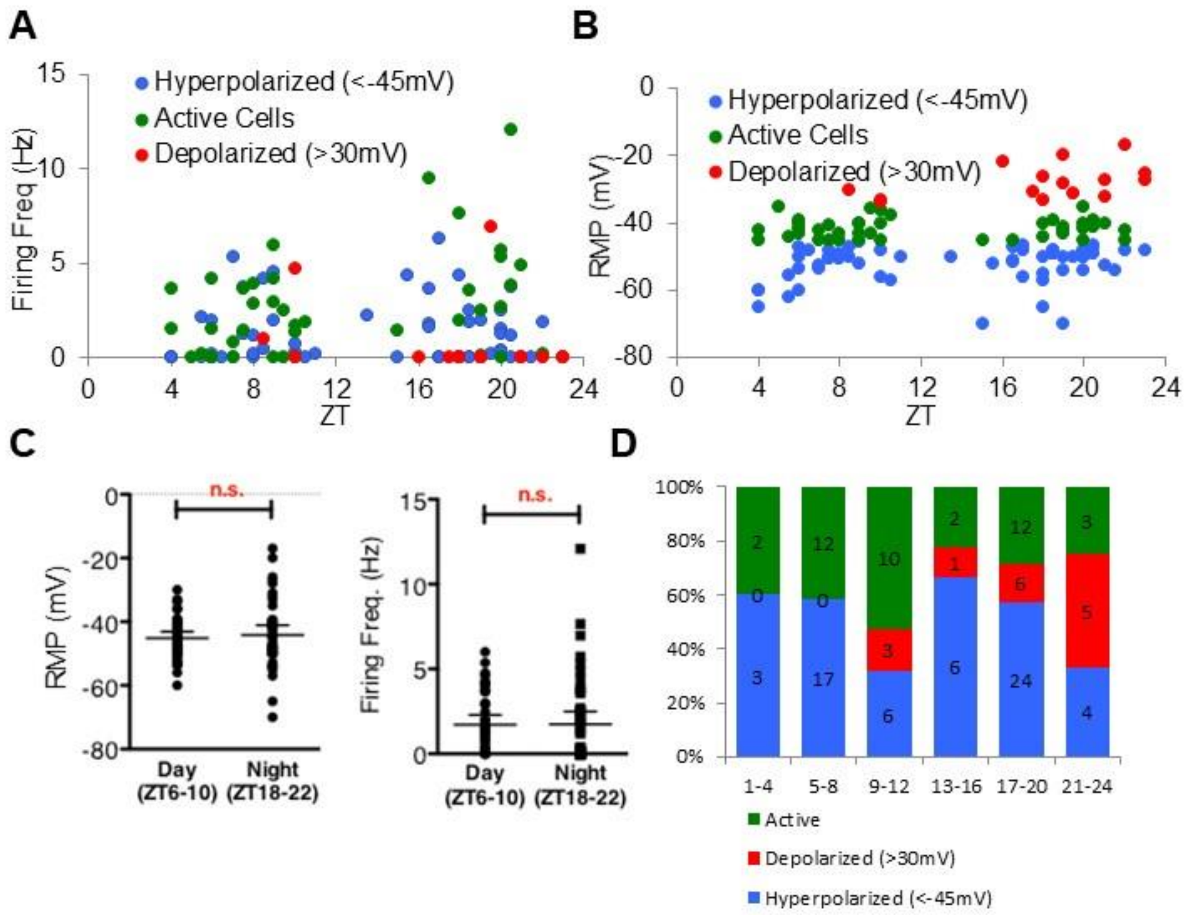

Figure S2: Characterization of night active SCN neurons by patch clamp

Related to Fig 1

A-D) Patch clamp recordings from randomly selected SCN neurons around the clock. Active neurons are shown in green, neurons that were so depolarized that they could not fire (Resting Membrane Potential (RMP)>--30mV) are shown in red, and neurons that were hyperpolarized and could not fire (RMP<-45mV) are shown in blue. **A)** Firing frequency (Hz) of SCN neurons over time. **B)** RMP of SCN neurons over time. **C)** There is no difference in RMP or firing frequency between active SCN neurons recorded during the night (ZT12-24) or day (ZT0-12). **D)** Percentage distribution of the 3 classes of SCN neuron, with recordings divided into six 4h time bins. During the night, the greatest number of active neurons are detected between ZT17 and 22, roughly coincident with the daily siesta.

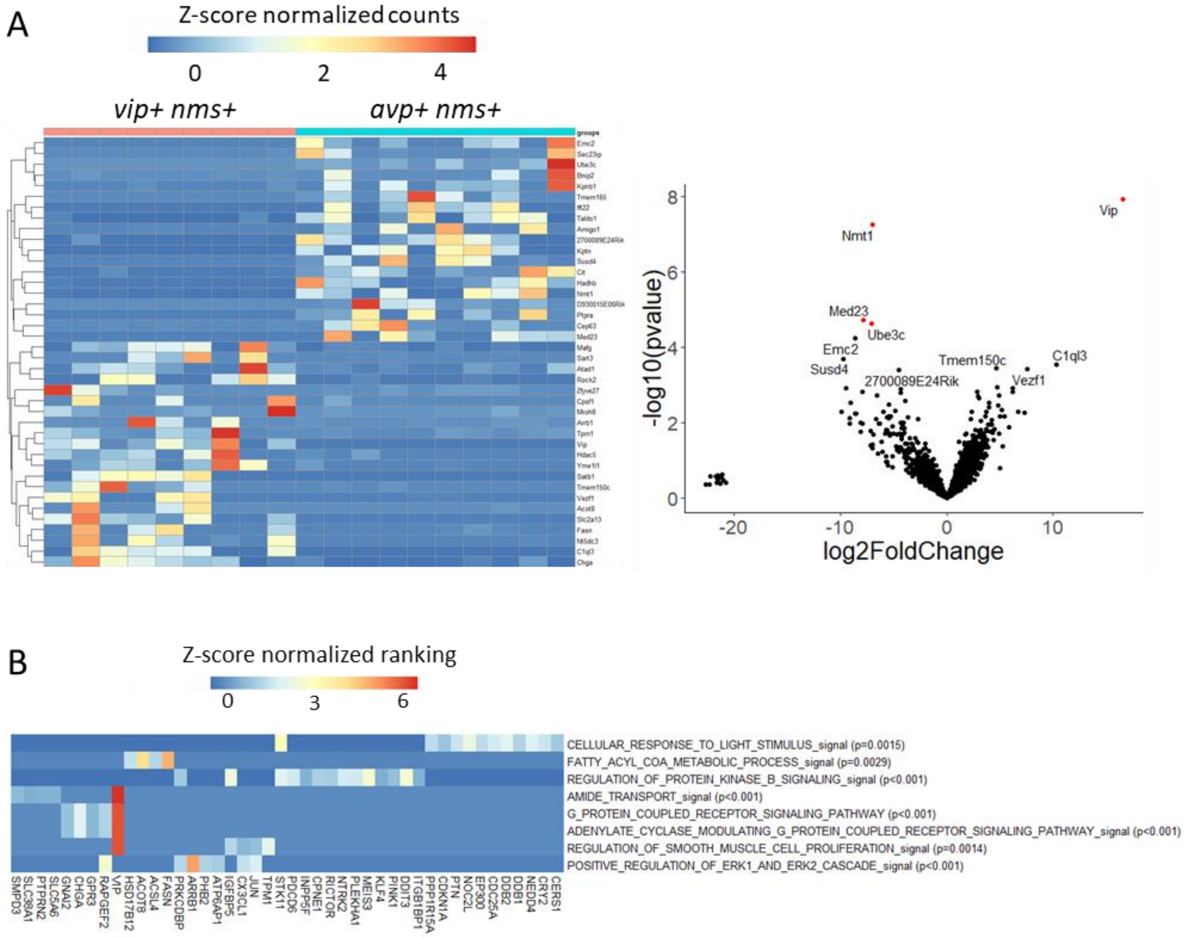

Figure S3: Differential gene expression analysis and pathway analysis between *avp+ nms+* and *vip+ nms+* neurons. Related to Fig 2.

A) Gene expression differences between *vip+ nms+* and *avp+ nms+* cFOS::GFP+ neurons using DESeq2. **Left,** heatmap of the 40 most differentially expressed genes, z-scored by row. **Right,** volcano plot comparison of gene expression between types. Red dots, transcripts with padj<0.01. Genes enriched in *vip+ nms+* cFOS::GFP+ neurons are located to the right. **B)** Gene Set Enrichment analysis. Heatmap showing genes contributing to the most represented gene ontology terms upregulated in *vip+ nms+* neurons compared to a*vp+ nms+* neurons. Color code represents the gene ranking calculated based on DESEq2 results. Nominal pvalue is indicated for each GO term.

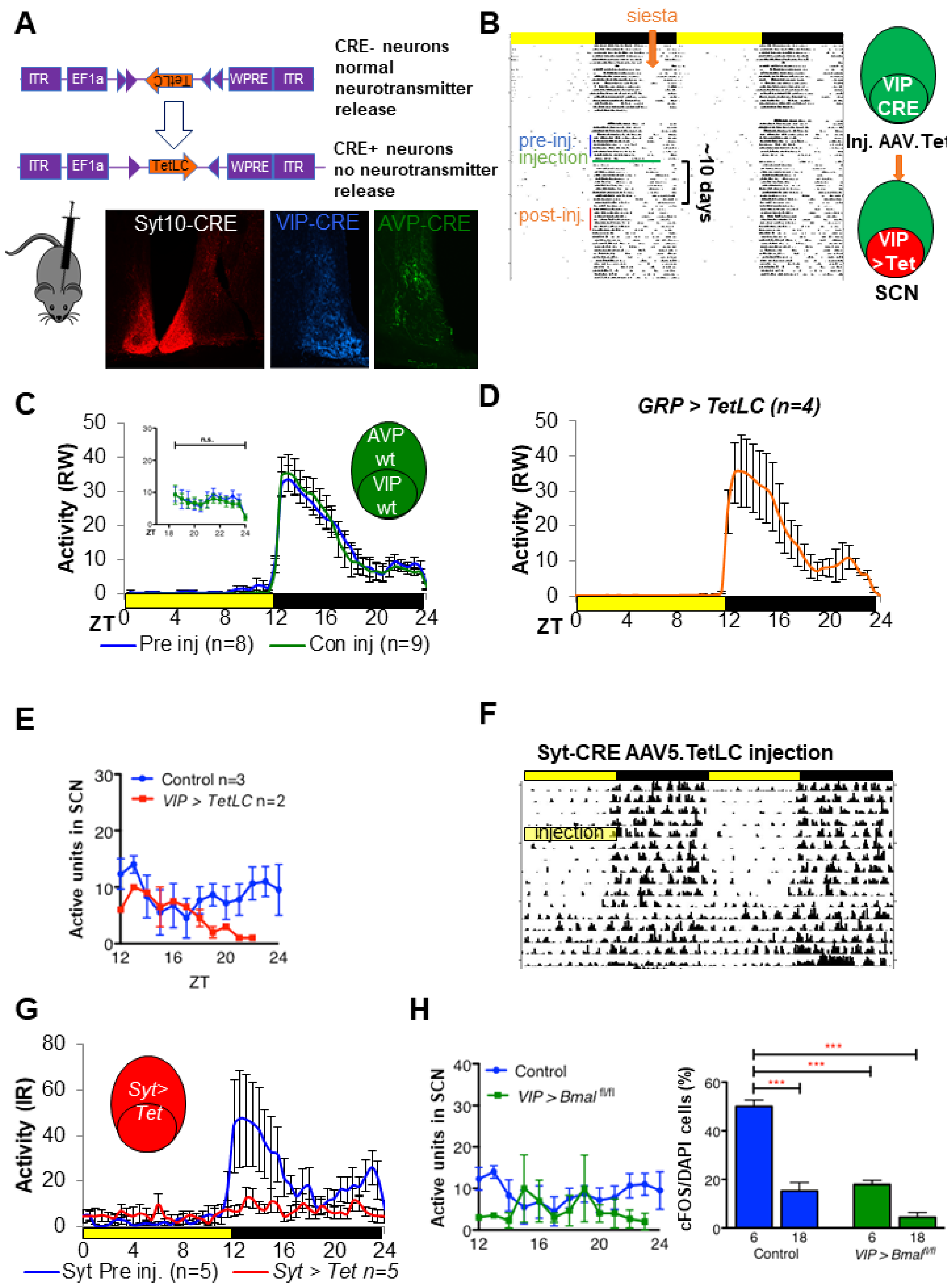

Figure S4: Blocking electrical activity in the SCN affects RW behavior. Related to Fig 3. Error bars represent SEM. ***P<0.005; ****P<0.0001.

A) Top: mice were injected with a CRE-dependent *AAV.Flex.TetLC* virus targeted to the SCN, blocking synaptic transmission from CRE+ SCN neurons. **Bottom:** Examples of *Syt10-CRE*, *Vip-CRE* and *Avp-CRE* driven expression of CRE-dependent YFP in the SCN. **B)** Representative actogram showing the effect of injection of *AAV.Flex.TetLC* into the SCN of *Vip-CRE* mice on RW activity under 12:12 light:dark cycles. Blue bar represents days of data used to plot activity before injection, orange arrow indicates daily siesta, yellow box indicates day of injection, and red bar indicates days used to plot post-injection activity. **C)** Average RW plots showing no effect of injection of control virus into *Vip-CRE* mice. The average of 7 days RW activity in 30 min bins is plotted pre-injection (blue line), compared to 7 days RW activity at least 2 weeks after injection (green line), when the virus should be fully expressed. ($F_{1,180}$=0.6722, n.s.)

D) Injection of *AAV.Flex.TetLC* into the SCN of *Grp-CRE* mice does not disrupt the siesta, as measured by RW activity. **E)** *AAV.Flex.TetLC* reduces VIP neuronal activity within the SCN. Depicted is the number of active units detected from SCN slices on measured in vitro by multielectrode array (MEA) over time. Active units are reduced specifically during the siesta (n=2) in *VIP>TetLC* vs control SCN slices. **E-F)** Synaptic transmission from the SCN is required for normal rhythms under LD cycles. *Syt10-CRE* mice were injected at the SCN with *AAV.Flex.TetLC*, blocking synaptic transmission from all CRE-expressing neurons in the SCN. **E)** Representative actogram of infrared activity under 12:12LD, showing the loss of rhythmicity after injection of *AAV.Flex.TetLC* into the SCN of a *Syt10-CRE* mouse. Yellow box indicates day of injection. **F)** Average activity of infrared behavior under 12:12LD cycles of 5 mice that became arrhythmic after *AAV.Flex.TetLC* injection (blue line pre-injection, red line post injection). Infrared, rather than running wheel activity is shown, as *Syt10-CRE* mice showed reduced to zero running wheel activity post injection. **G)** Left: *VIP>Bmal*$^{fl/fl}$ mice show reduced electrical activity within the SCN, as measured by MEA. Fewer detectable units were observed at night in *VIP>Bmal*$^{fl/fl}$ mice (green) than in controls. Right: cFOS expression is significantly reduced at both ZT6 and ZT18 in *VIP>Bmal*$^{fl/fl}$ SCN slices (green) compared to controls (blue); Statistics: one-way ANOVA with Tukey's post hoc comparisons. $F_{3,11}$=28.79; P<0.0001.

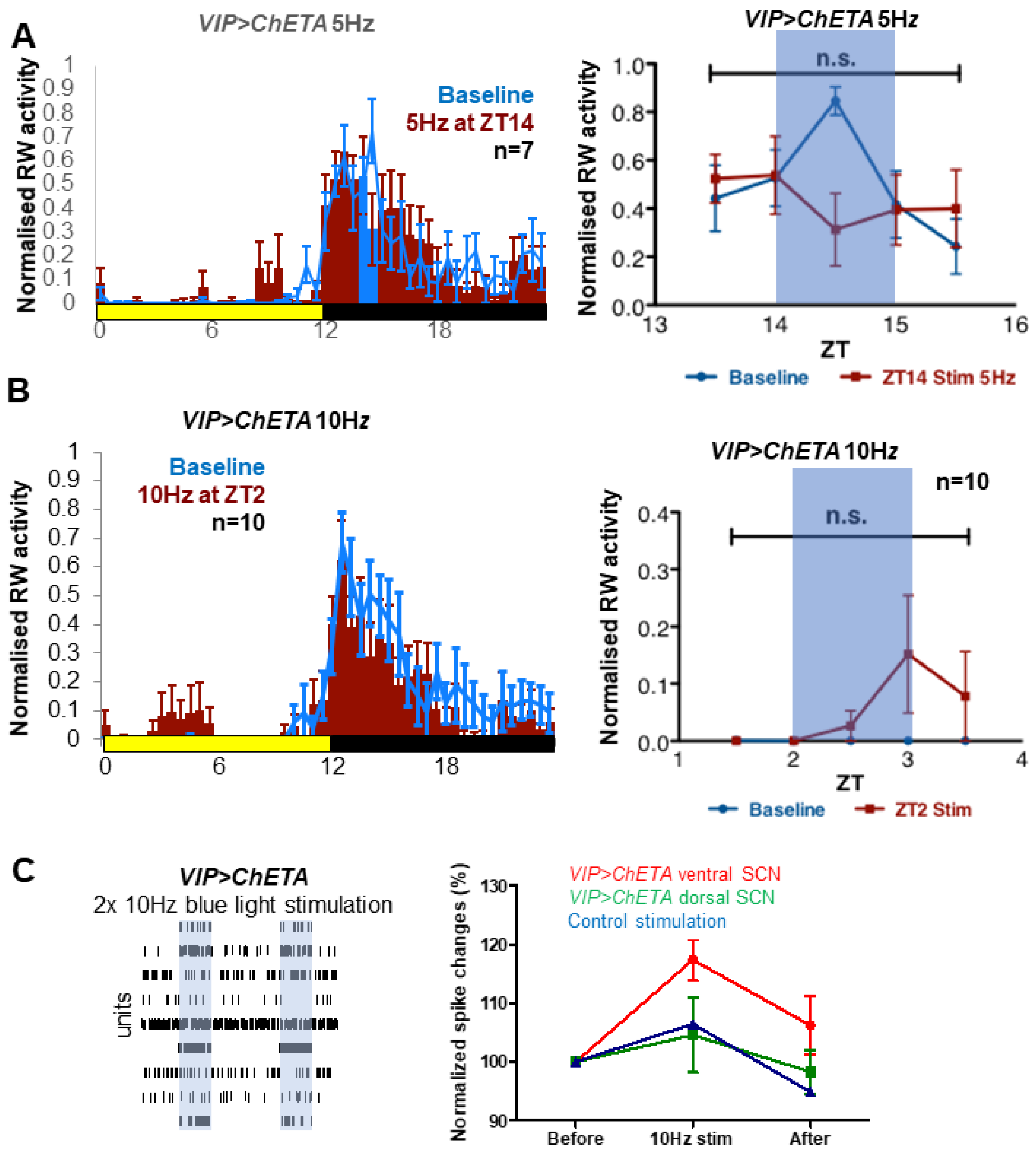

Figure S5: Effect of optogenetic stimulation of VIP+ SCN neurons on RW activity
Related to Fig 3.

Vip-CRE mice were injected at the SCN with the CRE-dependent AAV *VIP>ChETA*, All stimulations (10ms light at 473nm) are for 1hr at the specified frequency at the time specified, and RW activity is plotted in 30 min bins. Blue bars indicate time of stimulation. Error bars represent SEM. All statistical comparisons are by 2 Way ANOVA unless specified. *P<0.05 **A)** Optogenetic stimulation of VIP+ SCN neurons (Fig 3) was repeated at 5Hz. Left: Running wheel activity of *VIP >ChETA* before (baseline, blue line) or after stimulation at ZT14-15 at 5Hz (red and blue bars). Right: Stimulation of *VIP >ChETA* at 5Hz has no significant effect overall on running wheel activity ($P_{1,60}$=0.54, n.s.; however, timepoint 14.5h alone does show significant suppression of activity, p=0-0061 student T-test). **B) left:** RW activity at baseline (blue line) and after stimulation(red bars) at 10Hz at ZT2 (no RW

activity, so blue bar not visible) in *VIP>ChETA* mice. **Right:** statistical comparison of same. Apparent increase in RW activity after stimulation at ZT2 is due to a single mouse (1 of 10 included in plot) increasing running after stimulation; overall there is no significant effect on RW activity due to VIP+ SCN neuron activation at ZT2 ($F_{1,88}$=1.135, n.s.). **C)** MEA recording of *VIP>ChETA* SCN slice before, during (blue shading) and after 10Hz stimulation with 473nm light. **Left,** sample units responding to optogenetically driven activity or indifferent to it. (Units mirroring the 10Hz stimulation itself are not depicted.) **Right,** quantification of all units detected, shown as a percentage of baseline activity for dorsal (green) and ventral (blue) units, and for a control SCN slice lacking ChETA.

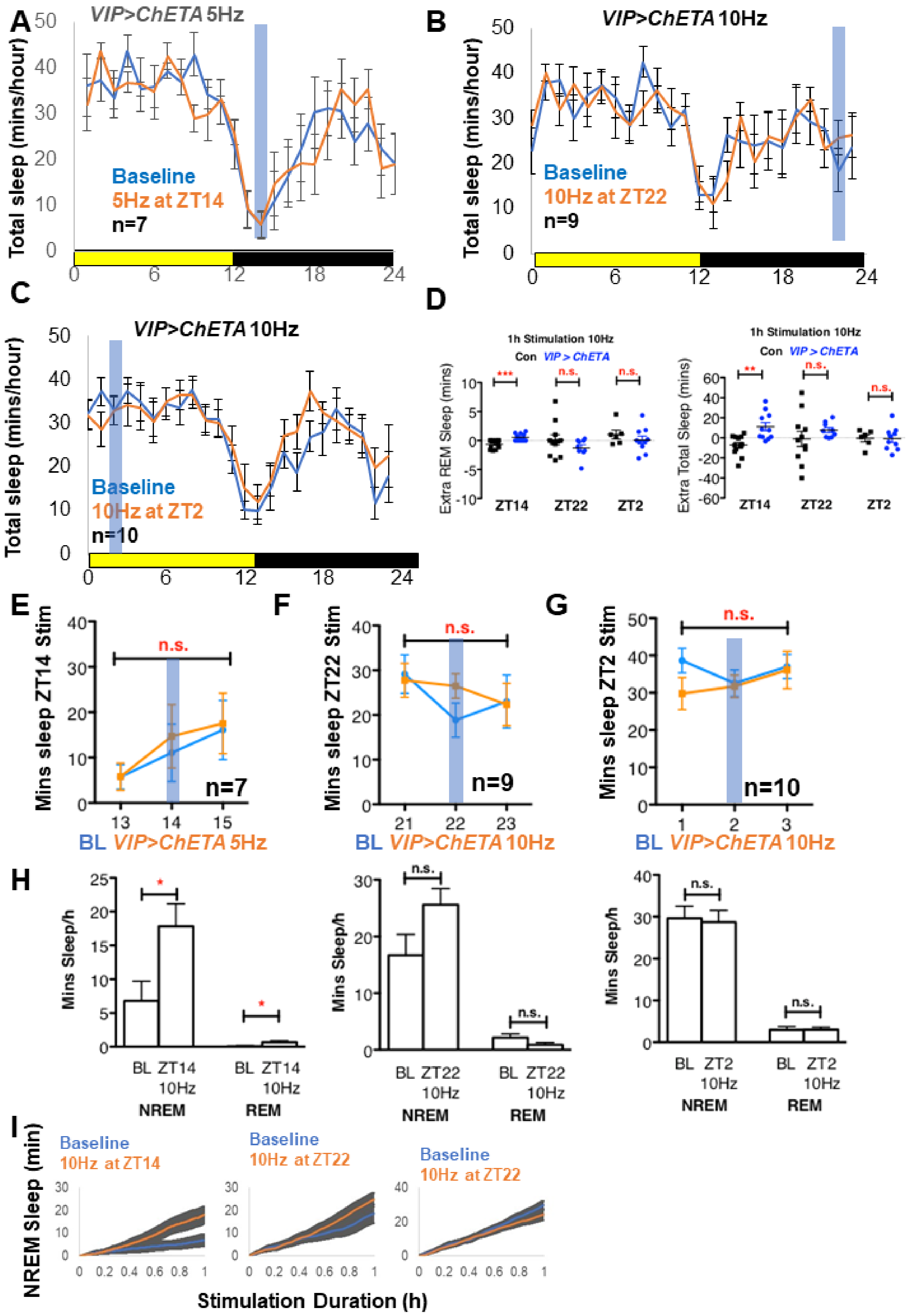

Fig S6: VIP+ SCN neurons regulate sleep during the dark phase

Related to Fig 4.

A-C) Sleep was recorded simultaneously with RW activity during optogenetic stimulations in *VIP>ChETA* mice. Total sleep is plotted in 1h bins for baseline (blue) or on the day of optogenetic stimulation of *VIP>ChETA* mice at 473nm/1h/10Hz (orange). **A)** Stimulation at 5Hz at ZT14 (no effect on sleep) **B)** Stimulation at 10Hz at ZT22 (small, non-significant increase in sleep) **C)** Stimulation at 10Hz at ZT2 (no effect on sleep). Time of stimulation is indicated by blue bar. Error bars represent SEM. Statistical comparisons by 2-tailed Student's t-test unless otherwise specified *P<0.05; **P<0.01; ***P<0.005; Yellow/Black bars represent 12:12LD cycle. **D)** Change in REM and total sleep during stimulation at ZT14, ZT22 or ZT2. (NREM in Fig 4D.) Control, black; *VIP>ChETA*, blue. Note that total and REM sleep are significantly increased in *VIP>ChETA* mice during stimulation at ZT14, but not at ZT22 or ZT2. At ZT22 there is a reduction in the variance in Total Sleep between mice [F test to compare variance: Total sleep $F_{10,8}$=11.17, P<0.01; REM sleep $F_{10,8}$=3.401, n.s.; no differences in variance at ZT22 or ZT2]. **E)** Stimulation of VIP+ SCN neurons at 5Hz had no effect on total sleep at ZT14 (2 way ANOVA, $F_{1,36}$=0.1377) **F)** Stimulation of VIP+ SCN neurons at 10Hz had no effect on total sleep at ZT22 ($F_{1,48}$=0.2775) or **G)** ZT2 ($F_{1,54}$=1.311). **H)** Minutes of NREM and REM sleep were calculated for baseline and during stimulation at ZT14, ZT22 and ZT2 in *VIP>ChETA* mice. Both NREM and REM sleep are significantly increased upon 1h 10Hz stimulation at ZT14. **I)** Cumulative NREM sleep is plotted compared to baseline for *VIP>ChETA* mice during 1h stimulation at ZT14, 22 or 2.

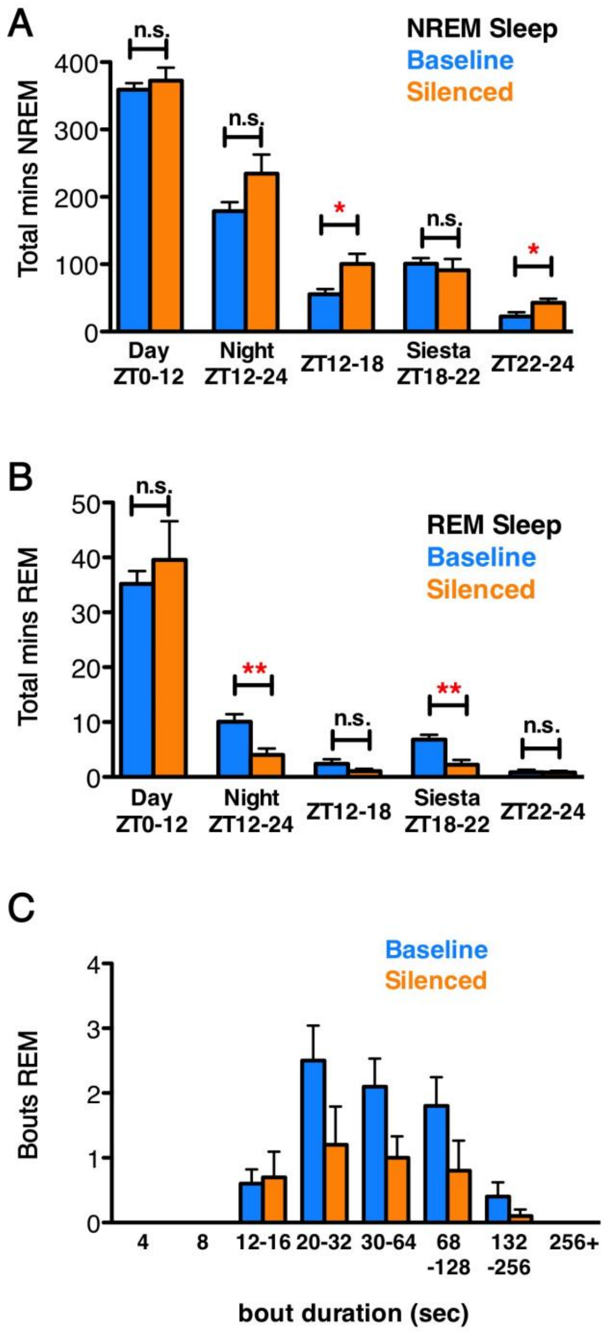
A
NREM Sleep
Baseline
Silenced
Total mins NREM
400
300
200
100
0
n.s.
n.s.
*
n.s.
*
Day ZT0-12
Night ZT12-24
ZT12-18
Siesta ZT18-22
ZT22-24
B
REM Sleep
Baseline
Silenced
Total mins REM
50
40
30
20
10
0
n.s.
**
n.s.
**
n.s.
Day ZT0-12
Night ZT12-24
ZT12-18
Siesta ZT18-22
ZT22-24
C
Baseline
Silenced
Bouts REM
4
3
2
1
0
4
8
12-16
20-32
30-64
68 -128
132 -256
256+
bout duration (sec)

Figure S7: Blocking synaptic transmission from VIP+ SCN neurons disrupts NREM and REM sleep. Related to Fig 4.

A-B) The amount of NREM **(A)** and REM sleep **(B)** during baseline (blue) and post-silencing (orange) sleep recordings, is quantified. There is no overall difference in NREM sleep between baseline and post-silencing recordings during the day or night, but REM sleep is significantly reduced at night in post-silencing recordings. NREM sleep is significantly increased from ZT12-18 and 22-24 (but not 18-22), whilst REM sleep is significantly decreased from ZT18-22, during the siesta (n=5).

C) The number of REM sleep bouts for baseline (blue) and post-silencing (orange) sleep recordings. There is a significant reduction in the number of long REM bouts after silencing of VIP neurons. All comparisons by 2 tailed Student's t-test *P<0.05, **P<0.01

	predicted.id	Prediction score for: *cck+c1ql3+*	*grp+vip+*	*avp+nms+*	*vip+nms+*	*cck+bdnf+*
**C1**	*avp+nms+*	0.021353623	0.00026924	0.571027769	0.400841347	0.006508021
**C2**	*avp+nms+*	0.193437347	0	0.615400778	0.034996949	0.156164926
**C3**	*avp+nms+*	0.05247504	0.001083735	0.903832172	0.03916144	0.003447613
**C4**	*avp+nms+*	0.07961211	0.284657091	0.633412218	0.00231858	0
**C5**	*avp+nms+*	0.101066818	0.011250188	0.615044044	0.237125087	0.035513864
**C6**	*avp+nms+*	0.005699213	0.174127868	0.617451093	0.129800636	0.072921189
**C7**	*avp+nms+*	0.257903376	0	0.59290322	0.014766738	0.134426665
**C8**	*avp+nms+*	0.013169991	0.178772488	0.654811269	0.021025798	0.132220454
**C9**	*vip+nms+*	0.028154312	0.236276879	0.123316132	0.591409949	0.020842729
**C10**	*avp+nms+*	0.221928656	0	0.724242359	0.04157603	0.012252955
**C11**	*vip+nms+*	0.247018916	0.028005422	0.068808226	0.656167436	0
**C12**	*grp+vip+*	0.00043107	0.917710029	0.025511705	0.055070915	0.001276281
**C13**	*vip+nms+*	0.172252056	0.120785151	0.042369427	0.589388112	0.075205254
**C14**	*vip+nms+*	0.01590468	0.00916313	0.241732322	0.725261153	0.007938715
**C15**	*vip+nms+*	0.172777742	0.053196529	0.176805979	0.586531736	0.010688015
**C16**	*vip+nms+*	0.032138468	0.023151609	0.026930338	0.917779585	0
**C17**	*vip+nms+*	0.104622129	0.161502066	0.183401159	0.550474646	0
**C18**	*vip+nms+*	0.26096296	0.019058914	0.024862168	0.695115958	0
**C19**	*avp+nms+*	0.056619818	0.137631003	0.605896805	0.005711688	0.194140686
**C20**	*vip+nms+*	0.033389867	0.167181893	0.244379047	0.555049193	0

Table S1. Prediction scores for collected cFOS-GFP+ neurons. Related to Fig 2.

SCN slices were collected from cFOS::GFP mice between ZT12-24. GFP+ neurons were identified visually, and electrical activity was recorded in different neurons by patch clamp. Cell contents of these neurons were then collected subjected to single-cell RNA sequencing (scSEQ). Transcriptomes

were matched against the scSEQ atlas of SCN neurons (Wen et al., 2020). Prediction scores for each neuron for each of the 5 classes in Wen et al are shown. 0=no transcriptome similarity in discriminating genes; 1=complete transcriptome similarity in discriminating genes. The subtype with the highest prediction score for each cell is the predicted neuron ID.

Cell	Celltype	ZT	C (pF)	iR (MΩ)	sR (MΩ)	RMP (mV)	FF (Hz)
**C1**	*avp+nms+*	15	17.5	749	22.2	-48	5.03
**C2**	*avp+nms+*	15	7.6	594	21.8	-57.9	6.30
**C3**	*avp+nms+*	15	17.0	725	16.5	-53	4.04
**C4**	*avp+nms+*	15.5	27.9	514	21.3	-41	10.61
**C5**	*avp+nms+*	15.5	11.6	569	15.6	-35	0.59
**C6**	*avp+nms+*	15.5	31.3	347	15.9	-58	0.00
**C7**	*avp+nms+*	16	14.1	2708	24.1	-61	2.02
**C8**	*avp+nms+*	16	19.4	3261	23.3	-57	0.08
**C9**	*vip+nms+*	16	12.8	938	16.5	-50	2.87
**C10**	*avp+nms+*	16.5	17.3	2591	19.7	-60	0.47
**C11**	*vip+nms+*	16.5	12.3	843	15.1	-54	5.66
**C12**	*grp+vip+*	17.5	13.0	1242	22.6	-63	1.11
**C13**	*vip+nms+*	17.5	11.3	583	17.1	-53	0.13
**C14**	*vip+nms+*	17.5	13.3	3194	17.6	-58	1.84
**C15**	*vip+nms+*	17.5	12.0	1676	18.9	-46	2.63
**C16**	*vip+nms+*	18	14.7	1376	18.0	-47	1.55
**C17**	*vip+nms+*	18.5	13.0	1581	16.1	-45	2.79
**C18**	*vip+nms+*	19	20.0	690	18.5	-47	3.71
**C19**	*avp+nms+*	19	31.1	556	13.2	-49	3.50
**C20**	*vip+nms+*	19.5	12.3	494	22.4	-57	6.65

Table S2. Electrophysiological properties of SCN neurons from Patch-seq data. Related to Fig 2

Membrane Capacitance (pF), input Resistance (MΩ) and series Resistance (MΩ) were measured in voltage-clamp mode. Resting Membrane Potential (mV) and Firing Frequency (Hz) were measured in current-clamp mode.
