# Supplementary material for: Circadian VIPergic Neurons of the Suprachiasmatic Nuclei Sculpt the Sleep-Wake Cycle
Source: Neuron. 2020 Nov 11;108(3):486–499.e5. doi: 10.1016/j.neuron.2020.08.001 (PMC7803671; doi:10.1016/j.neuron.2020.08.001)
Supplement: Document S2. Article plus Supplemental Information [file mmc2.pdf]

Neuron

Article

Circadian VIPergic Neurons of the Suprachiasmatic Nuclei Sculpt the Sleep-Wake Cycle

Highlights

- Specific VIP+ neurons of the SCN fire at night when most others are silent
- Activating or silencing these VIP+ neurons controls nighttime but not daytime sleep
- Circadian clockwork within these specific neurons times the daily “siesta”
- The activity of these neurons, in turn, programs subsequent end-of-day alertness

Authors

Ben Collins, Sara Pierre-Ferrer, Christine Muheim, ..., Csaba Földy, Antoine Adamantidis, Steven A. Brown

Correspondence

ben.collins01@gmail.com (B.C.),
steven.brown@pharma.uzh.ch (S.A.B.)

In Brief

The “master” circadian clock generally indicates day and night by tonic daytime firing. A specific population of neurons, active when the rest of the SCN is silent, directs nighttime “siesta” sleep but not daytime sleep, thereby timing end-of-day alertness.

Check for updates

Collins et al., 2020, Neuron *108*, 486–499
November 11, 2020 © 2020 Elsevier Inc.
https://doi.org/10.1016/j.neuron.2020.08.001

CellPress

CellPress

Neuron

Article

Circadian VIPergic Neurons of the Suprachiasmatic Nuclei Sculpt the Sleep-Wake Cycle

Ben Collins,[1,11,*] Sara Pierre-Ferrer,[1,11] Christine Muheim,[1,2] David Lukacsovich,[3] Yuchen Cai,[4] Andrea Spinnler,[1] Carolina Gutierrez Herrera,[5] Shao'Ang Wen,[4] Jochen Winterer,[3] Mino D.C. Belle,[6] Hugh D. Piggins,[7] Michael Hastings,[8] Andrew Loudon,[9] Jun Yan,[4] Csaba Földy,[3] Antoine Adamantidis,[5,10] and Steven A. Brown[1,12,*]

[1]Chronobiology and Sleep Research Group, Institute of Pharmacology and Toxicology, University of Zürich, Winterthurerstrasse 190, 8057 Zürich, Switzerland
[2]Department of Biomedical Sciences, Washington State University, Spokane, WA 99202, USA
[3]Laboratory of Neural Connectivity, Brain Research Institute, University of Zürich, Winterthurerstrasse 190, 8057 Zürich, Switzerland
[4]Institute of Neuroscience, Chinese Academy of Sciences, 320 Yueyang Road, Shanghai 200031, P.R. China
[5]Department of Neurology, Inselspital University Hospital Bern, Freiburgstrasse 18, 3010 Bern, Switzerland
[6]Institute of Biomedical and Clinical Sciences, University of Exeter Medical School, University of Exeter, Exeter EX4 4PS, UK
[7]School of Physiology, Pharmacy, and Neuroscience, University of Bristol, Bristol BS8 1TH, UK
[8]Division of Neurobiology, MRC Laboratory of Molecular Biology, Cambridge CB2 0QH, UK
[9]Centre for Biological Timing, Faculty of Biology, Medicine and Health, School of Medical Sciences, University of Manchester, Manchester M13 9PT, UK
[10]Department of Biomedical Research, Inselspital University Hospital Bern, Freiburgstrasse 18, 3010 Bern, Switzerland
[11]These authors contributed equally
[12]Lead Contact
*Correspondence: ben.collins01@gmail.com (B.C.), steven.brown@pharma.uzh.ch (S.A.B.)
https://doi.org/10.1016/j.neuron.2020.08.001

SUMMARY

Although the mammalian rest-activity cycle is controlled by a "master clock" in the suprachiasmatic nucleus (SCN) of the hypothalamus, it is unclear how firing of individual SCN neurons gates individual features of daily activity. Here, we demonstrate that a specific transcriptomically identified population of mouse VIP+ SCN neurons is active at the "wrong" time of day—nighttime—when most SCN neurons are silent. Using chemogenetic and optogenetic strategies, we show that these neurons and their cellular clocks are necessary and sufficient to gate and time nighttime sleep but have no effect upon daytime sleep. We propose that mouse nighttime sleep, analogous to the human siesta, is a "hard-wired" property gated by specific neurons of the master clock to favor subsequent alertness prior to dawn (a circadian "wake maintenance zone"). Thus, the SCN is not simply a 24-h metronome: specific populations sculpt critical features of the sleep-wake cycle.

INTRODUCTION

Almost all organisms have internal circadian clocks that consolidate sleep to night in diurnal organisms or day in nocturnal ones. Sleep duration and intensity are additionally regulated by a homeostatic mechanism dependent upon prior waking experience (Borbély, 1982). Within this two-process model, it remains unclear to what extent the circadian clock acts as a "metronome"—indicating day and night—versus acting as an "orchestral conductor"—specifically shaping features of the sleep-wake cycle. Most animal species and all human cultures consolidate the majority of sleep to either day or night. Many also exhibit an additional period of sleep—a "siesta"—during the second half of the wake phase, followed by a period of increased alertness—the "wake maintenance zone" (WMZ)—thought to depend on sleep pressure (Ehlen et al., 2015; Owens et al., 2010; Reichert et al., 2014). How the mammalian circadian oscillator shapes this complex pattern is unknown.

The molecular basis of circadian behavior is based on transcription-translation feedback loops that oscillate every 24 h. Molecular rhythms in peripheral cells are entrained by a central clock located in the suprachiasmatic nucleus (SCN) 20,000 neurons at the base of the hypothalamus (Brown and Azzi, 2013). Overall, SCN neurons are most electrically active during the day; in mice, this correlates with periods of quiescence and sleep (Belle et al., 2009; Colwell, 2011; Lee et al., 2009), and blocking SCN neuronal firing during the day with tetrodotoxin (TTX) results in increased locomotor activity (Houben et al., 2014). Within the SCN circadian clock, genes and neuronal networks cooperate to generate robust rhythms and then translate these cellular oscillations into daily rhythms of behavior that persist under constant conditions (An et al., 2011; Aton et al., 2005, 2006; Brancaccio et al., 2013; Freeman et al., 2013; Harmar et al., 2002; Maywood et al., 2006; O'Neill et al., 2008). Although all SCN neurons contain the same molecular clock, anatomically distinct regions of the SCN—a ventral "core" expressing vasoactive intestinal

 © 2020 Elsevier Inc.

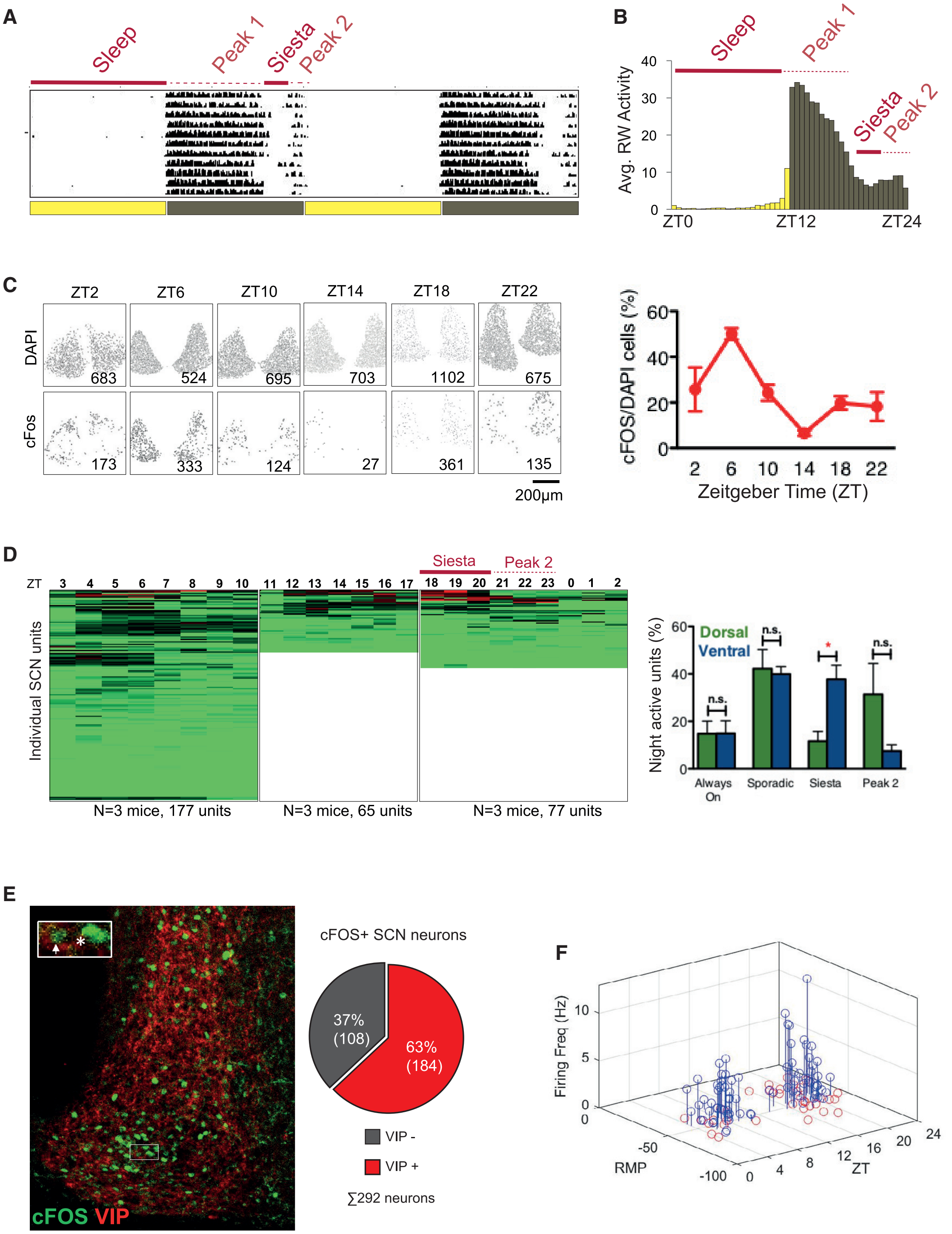

Figure 1. Identification of Night-Active SCN Neurons

(A) Representative actogram of wild-type mouse RW activity in LD12:12. Note 2 peaks of RW activity separated by a period of quiescence and sleep, the siesta.

(B) Average RW activity of 18 mice recorded for 7 days plotted in 30-min bins. Yellow bars indicate light; black bars indicate dark.

(C) Left: representative immunostained SCN slices collected at indicated times (ZT, time relative to light onset). Top: DAPI (cell bodies). Bottom: c-FOS (marker of neuronal activity). Images were processed to remove background; only positive cells are shown. Inset: number of detected cells. Right: percentage of SCN DAPI+ cells co-expressing c-FOS (n = 3 animals per time point).

(legend continued on next page)

polypeptide (VIP) and a dorsal "shell" expressing arginine vasopressin (AVP) (Varadarajan et al., 2018)—display circadian gene expression in slightly different phases (Aton and Herzog, 2005), a pattern thought to be important for enabling clock plasticity encoding seasonality and day length (Azzi et al., 2014, 2017; Evans et al., 2013). It has been suggested that different oscillator populations within the SCN track dawn and dusk and drive distinct peaks of behavioral activity: the morning (M) and evening (E) oscillator model (Pittendrigh and Daan, 1976). Supporting this idea, in *Drosophila*, distinct groups of clock neurons display phases of electrical activity that anticipate dawn or dusk and promote M or E locomotor activity, respectively (Grima et al., 2004; Liang et al., 2016; Stoleru et al., 2004), while other populations of clock neurons regulate sleep timing (Guo et al., 2016; Parisky et al., 2008). So far, little behavioral evidence exists to support this idea in mammals, though the overall "firing window" of SCN neurons expands or contracts with changing day length (VanderLeest et al., 2007).

Here, we demonstrate that a specific population of mouse VIP+SCN neurons is active at the "wrong" time of day—nighttime when most SCN neurons are silent—and is necessary and sufficient to gate nighttime sleep but has no effect on daytime sleep. In turn, the molecular clock within these neurons determines the specific timing of this nighttime sleep (a rodent "siesta") and subsequent alertness prior to dawn (a circadian WMZ). Thus, the SCN is not just a metronome for 24 h rhythms but micromanages critical features of the sleep-wake cycle.

RESULTS

Identification of Night-Active SCN Neurons

Mouse running wheel (RW) behavior under 12:12 light:dark (LD) cycles includes 3 major time-of-day-dependent features: (1) a prolonged early-night activity peak starting just prior to dark onset at zeitgeber time (ZT)12, (2) a siesta (a period of quiescence and sleep) centered around ZT20, and (3) a second bout of activity anticipating lights on at ZT0 (Figures 1A and 1B). Although the SCN is most electrically active during the day (Colwell, 2011), for the SCN clock to control nighttime activity we hypothesized, some SCN neurons would be electrically active during one or more of these nighttime events. We therefore quantified SCN neuron electrical activity at six time points by immunostaining for the immediate-early protein c-FOS, a marker of neural activity (Schilling et al., 1991) (Figure 1C). Consistent with previous reports, significantly more neurons express c-FOS during the day than during the night (Colwell, 2011). However, populations of presumably active c-FOS-expressing neurons were identifiable at night in both dorsal and ventral regions of the SCN, albeit in lower numbers than during the day (Figure 1C). Interestingly, there appeared to be two peaks of firing, with the first (major) peak corresponding to the peak of daytime sleep and a second roughly corresponding to the peak of nighttime sleep (the siesta).

Characterization of Night-Active SCN Neurons

To characterize night-active SCN neurons in more detail, we placed SCN slices on a multi-electrode array (MEA), allowing recording of unit activity over time (Figure S1A). We maintained individual SCN slices on the MEA for 9–10 h, recording for 10 min/h, and then identified and sorted spikes by waveform to identify active units—individual neurons or electrically similar groups of neurons—at each time point. Although only ~16/59 electrodes contacted the SCN, 2/3 of all detected units came from within the SCN, with the main peak of SCN unit firing preceded firing from extra-SCN units by ~2 h (Figure S1B). During the day (ZT0–12), we detected 177 units active at one or more time points (3 experiments), with the highest number of active units detected between ~ZT5 and ZT8, during the daytime sleep period. At night (ZT16–24), we detected 77 active units across 3 experiments. Figure 1D shows these data as a proportionally compressed composite view, trimmed to eliminate overlapping recordings. (An expanded view of night-active neurons is shown in Figure S1C, representative recordings are in Figure S1D, and a composite sorting of all units is in Figure S1E.)

About 15% of night-active units (16/106; 4 experiments) were firing for the duration of the recording ("always on"), and ~40% (40/106) were active for a brief period at one or more time points ("sporadic"). Both of these were equally distributed between the dorsal and ventral SCNs. Of the remainder, a further ~20% (21/106) became active toward the end of the siesta and continued to fire to the end of the night (~ZT21–24), coincident with the second RW activity peak; these "2nd peak" active units were predominantly dorsal SCN neurons, consistent with previous reports of AVP+SCN neuronal activity at this time (Gizowski et al., 2016). The remaining ~25%, (25/106) were, by far, the most active, with a period of activity coincident with nighttime (siesta) sleep (~ZT18–22): an expanded heatmap showing these units is in Figure S1C. These "siesta" active units were predominantly ventral (Figure 1D, right panel). As the ventral core region of the SCN is defined by VIP expression, we looked for co-expression of VIP and c-FOS at ZT18, around the beginning of the siesta (Figure 1E). Consistent with our previous results, roughly two thirds of c-FOS-positive neurons at ZT18 were VIPergic (Figure 1E). We next determined whether siesta-active neurons fired

(D) Left: unit activity detected across the day. Each row represents a unit active at any time point from one of 3 acute slices; totals are at bottom. Columns indicate time. Three trimmed, overlapping sets of measurements shown. Green, 0 Hz; Red, 3 Hz. Full data and quantification are in Figure S1. Right: Dorsal/ventral location and type of activity for each unit. Note that a higher proportion of ventral units show siesta-specific activity.
(E) 40× maximum-projection images of SCN immunostained for c-FOS (green) and VIP (red) at ZT18. VIP+ axonal and dendritic staining is observed throughout the SCN, while VIP cell bodies are primarily ventral. Inset: arrow indicates neuron expressing VIP and c-FOS, Asterisk indicates neuron expressing only c-FOS. Pie chart: quantification of c-FOS and VIP cell body co-localization (red) versus c-FOS alone (gray); n = 6.
(F) Patch clamp recordings of equal numbers of wild-type neurons recorded from nighttime and daytime slices. Blue, active neurons; red, inactive neurons. Axes indicate time, resting membrane potential, and firing rate. 1 h gap between daytime and nighttime units due to time of slice preparation. Further analysis is in Figure S2.
Error bars represent SEM. *$p < 0.05$, 2-tailed Student's t test.
See also Figures S1 and S2.

only during the siesta or were also active along with the majority of SCN neurons during the light period. Strikingly, most siesta-active neurons were siesta specific: of 27 siesta-active units, 21 were active only during the siesta (Figure S1F). Another 6 units showed an on-off-on pattern, beginning to fire again at the start of the next day, but even these fired more vigorously during the siesta (Figure S1G).

To further investigate night-active SCN neurons, we recorded electrical activity from randomly selected SCN neurons around the clock by patch clamp: Figure 1F shows all recorded neurons in a 3D graph, and Figure S2 shows individual neuron characteristics. As previously reported (Belle et al., 2009), we found SCN neurons that were firing (active cells) and two classes of inactive neurons: one hyperpolarized class with a resting membrane potential (RMP) ≤ -45 mV and a second depolarized population with a RMP ≥ -30 mV. (We injected current to repolarize each depolarized cell and verify that it could fire normal healthy action potentials.) Scoring for the time when neuronal activity was recorded, two peaks of firing were again observed: one during the day and one during the night, with equal RMP and firing frequency (Figures 1F and S2A–S2C). The majority of night-active neurons were visible during the siesta period (Figure S2D).

Categorization of Night-Active SCN Neurons

To better characterize night-active SCN neurons, we used c-FOS::GFP transgenic mice (Reijmers et al., 2007), where sustained neuronal activity induces GFP expression for a ~3 h window. We took SCN slices from c-FOS::GFP mice to be ready to examine from ZT15 (~2 h prior to siesta onset), identified GFP+ neurons throughout the siesta, recorded electrical activity in these different neurons by patch clamp, and then collected the cell contents of these neurons and characterized their transcriptomes by single-cell RNA sequencing (scRNA-seq; Figure 2A). First, we examined neuropeptide expression relative to time of collection of each active neuron. As predicted, VIP is predominantly expressed in siesta-active neurons compared to pre-siesta-active neurons (Figure 2B). Since one limitation of scRNA-seq is that all expressed genes are not detected in each library, we characterized each neuron by matching transcriptomes against the recently published scRNA-seq atlas of SCN neurons (Wen et al., 2020) to classify their type (prediction scores are shown in Table S1; see STAR Methods). From 20 ventral c-FOS::GFP+ SCN neurons, we identified two classes of night-active neurons within the SCN: a *vip*+ *nms*+ population (n = 9) and an *avp*+ *nms*+ population (n = 10), as well as a single *vip*+ *grp*+ neuron (Figure 2C). As expected, ~75% of c-FOS::GFP+ neurons were electrically active at the time of collection (Figures 2D and 2E; c-FOS::GFP expression in non-active neurons likely represents recent prior electrical activity; see Table S2 for electrophysiological properties). Interestingly, *avp*+ *nms*+ SCN neurons were all collected in the early night (~ZT15–16, during the first peak of RW activity), while *vip*+ *nms*+ neurons were collected toward the end of the night (~ZT17–20, approximately the time of the siesta). These data suggest strongly that nighttime-active *vip*+ *nms*+ neurons are firing during the siesta, while *avp*+ *nms*+ nighttime-active neuronal activity precedes it.

VIP+ SCN Neurons Are Necessary for Normal Nighttime Sleep Patterns

Given that *avp*+ and *vip*+ SCN neurons are active at different times of night (Figure 2E), we hypothesized that each population could regulate different aspects of nighttime behavior. To test this, we measured the contribution of each subset of SCN neurons to RW activity using three strains of mice bred into a homogeneous C57bl6 background: *Vip-CRE* to target VIPergic neurons (Taniguchi et al., 2011), *Avp-CRE* to target AVPergic neurons (Harris et al., 2014), and *Syt10-CRE* to target all SCN neurons (Husse et al., 2011). Consistent with Franken et al. (2001), >90% of mice displayed a robust nighttime siesta in the presence of a RW; only mice displaying a daily siesta were used for subsequent experiments. Mice were injected at the SCN with either control or an *AAV.Flex.TetLC* virus (Adeno-associated virus) encoding tetanus toxin light chain (TetLC) in reverse orientation between *loxP* sites (Foster et al., 2015). Using this intersectional approach, in the presence of CRE, *TetLC* is flipped into the correct orientation and transcribed, blocking synaptic transmission (Figure S4A). We then compared RW activity in 12-h:12-h LD conditions for each mouse in the week prior to injection to RW activity in the week starting ~14 days post-injection, allowing TetLC to be fully expressed in VIP+SCN neurons (hereinafter abbreviated *VIP*>), AVP+SCN neurons (*AVP*>) or the entire SCN (*Syt10*>). Injection of *AAV.Flex.TetLC* into the SCN after mice have reached adulthood eliminates the possibility of developmental defects caused by silencing VIP neurons, although we cannot rule out unknown compensatory mechanisms occurring after silencing. Control-injected mice showed no differences in distribution of activity pre- and post-injection (Figures 3C and S4C). *AVP>TetLC* mice showed a significant increase in RW activity from ZT12–18 post-injection, suggesting that AVP neurons may contribute to daily activity in the early night; however, siesta behavior was unaffected (Figures 3A and 3C), consistent with *avp*+ *nms*+ SCN neurons being active in the early night only (Figure 2E). By contrast, *VIP>TetLC* disrupted the daily siesta, consistent with the late night activity of *vip*+ *nms*+ SCN neurons (Figure 2E): the only significant effect of blocking VIP+SCN signaling was to increase RW activity from ZT18.5–22 (Figure 3B). Furthermore, the timing of the siesta, as determined by the time of lowest RW activity in each mouse at ZT18–23 was delayed by ~2 h in *VIP>TetLC* mice compared to that in controls or *AVP>TetLC* mice (Figure 3C). An example *VIP>TetLC* actogram is shown in Figure S4B; individual mice where VIP+SCN activity is silenced no longer show a defined period of quiescence and, instead, display random bouts of activity and inactivity across the latter half of the night. It should be noted that, although TetLC blocks synaptic transmission (*VIP>TetLC* caused a significant decrease in electrical activity within VIP+SCN neurons; Figure S4E), it does not affect the release of neuropeptides stored in dense core vesicles. Thus, the neuropeptide VIP itself may still be released, and we observe no changes in circadian period in *VIP>TetLC* mice (data not shown), unlike in $vip^{-/-}$ mutants (Aton et al., 2005).

Since we also identified a single *vip*+ *grp*+ night-active neuron (Figure 2B), we performed *AAV.Flex.TetLC* injections on *Grp-CRE* mice (Sun et al., 2017): silencing of GRP+SCN neurons alone was insufficient to disrupt siesta behavior

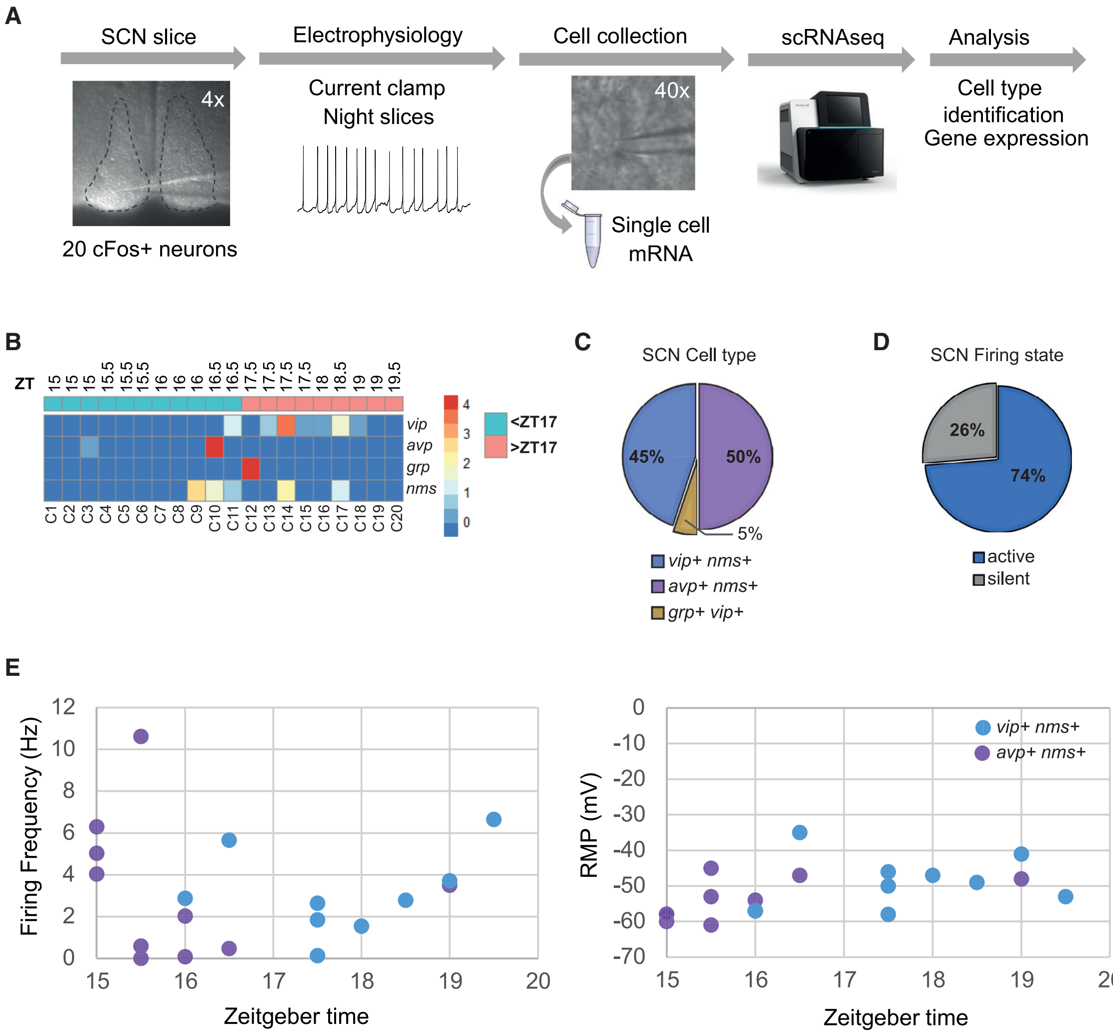

Figure 2. Characterization of Night-Active SCN Neurons by Patch-RNA-Seq

(A) Ventral c-FOS::GFP+ neurons were recorded from ZT15–20 by patch clamp, then cytoplasm was collected for scRNA-seq (n = 20 neurons).

(B) Heatmap showing expression of *vip*, *avp*, *grp*, and *nms* in neurons firing before and after ZT17. Color code shows *Z*-score normalized reads. Neurons firing after ZT17 express more *vip* compared to neurons firing before ZT17.

(C) Pie chart of identified SCN neuronal classes, as defined by projecting each transcriptome to the SCN Drop-seq atlas (Wen et al., 2020).

(D) Proportion of active (firing rate, >1 Hz) and silent neurons.

(E) Electrophysiological properties of neurons from the two major classes: *avp+ nms+* and *vip+ nms+*. Plots show firing frequency (in Hz; left) and resting membrane potential (RMP, in mV; right) over time. *avp+ nms+* c-FOS::GFP+ neurons are mostly active in the early night (<ZT17) and *vip+ nms+* c-FOS::GFP+ neurons from ~ZT17.

(Figure S4D). Interestingly, blocking synaptic transmission from the entire SCN (*Syt>TetLC*) results in ~50% of mice becoming arrhythmic, even under LD cycles ~10–15 days post-injection (representative mouse is shown in Figure S4F; average activity is shown in Figure S4G). This is in contrast to a previous report where ablation of the SCN did not prevent mice entraining to LD cycles (Schwartz and Zimmerman, 1991), but it is consistent with another report where SCN ablation blocked daily rhythms of sleep under LD cycles (Easton et al., 2004) as well as one in which TTX silencing of SCN neurons provoked acute activity (Houben et al., 2014).

Activating VIP+SCN Neurons Inhibits Nighttime Activity

To confirm that VIP+SCN neurons regulate nighttime behavior, we next tested the effect of activating VIP neurons on RW activity. We injected the SCN of *Vip-CRE* mice with an AAV virus encoding a CRE-dependent version of the optogenetic tool ChETA (Gunaydin et al., 2010) (*VIP>ChETA*), which causes a neuron to fire upon stimulation with blue light (473 nm). We first asked what happened when we activated VIP neurons at ZT14, when most SCN neurons are normally inactive and mice display the first peak in RW activity. Stimulation of VIP+SCN neurons was achieved through illumination with 10 ms pulses of light at

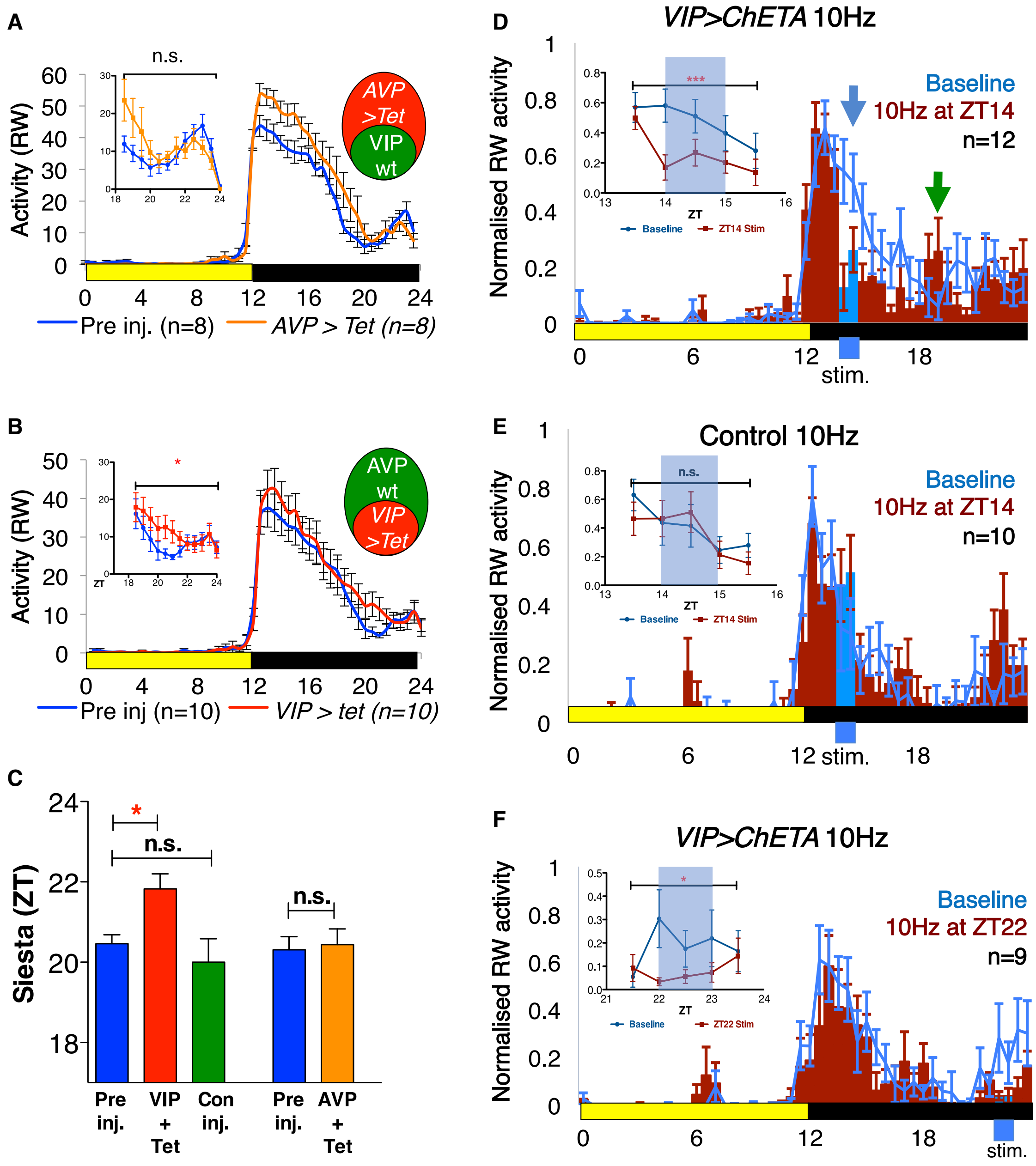

Figure 3. Neuronal Activity in VIP+SCN Neurons Regulates the Daily Siesta

(A–C) RW activity of *Vip-CRE* and *Avp-CRE* mice injected at the SCN with AAV.Flex.TetLC. An average of 7 days of RW activity in 30 min bins is plotted for each genotype pre-injection (blue line), compared to 7 days of RW activity ≥2 weeks post-injection (green, orange, and red lines) when the virus should be fully expressed. Inset: ZT18.5–23.5 RW activity, 2-way ANOVA. (A) No effect of injection of TetLC virus into AVP-CRE mice on siesta activity, $F(1, 168) = 2.776$, n.s. (B) Injection of TetLC virus into VIP-CRE mice significantly increases RW activity during the siesta, $F(1, 216) = 6.5$, $p < 0.05$. (C) Quantification of the effect of VIP+SCN neurons upon siesta timing, defined as the point of lowest activity during the siesta. Comparisons by ANOVA with Tukey's multiple comparison test for *VIP>Tet*: $F(2, 35) = 5.892$, $p = 0.0062$, otherwise Student's t test.

(D–F) RW activity of VIP-CRE mice injected at the SCN with the optogenetic probe AAV.ChETA. (D) RW activity of *VIP>ChETA* over 24 h before (baseline; blue line) or on day of stimulation (at 473 nm, which drives neurons to fire, via an optic fiber implanted between SCN lobes; red and blue bars). Stimulation for 1 h at 10 Hz at ZT14 (blue bars and blue arrow). Green arrow indicates new peak of RW activity that appears ~4 h post-stimulation (red bars, quantified in Figure S3). Inset: statistical summary before, during, and after stimulation; two-way ANOVA, $F(1, 106) = 12.15$, $p < 0.005$. (E) No effect of simulation for 1 h at 10 Hz at ZT14 on control-injected mice; $F(1, 80) = 0.28$, n.s. (F) Stimulation of VIP+SCN neurons for 1 h at 10Hz at ZT22 inhibits RW activity; $F(1, 75) = 4.95$, $p < 0.05$.

In all panels, yellow: black bars represent 12-h:12-h LD cycles. Statistical comparisons are as specified. Error bars represent SEM. *$p < 0.05$; **$p < 0.01$; ***$p < 0.005$.

See also Figures S4 and S5.

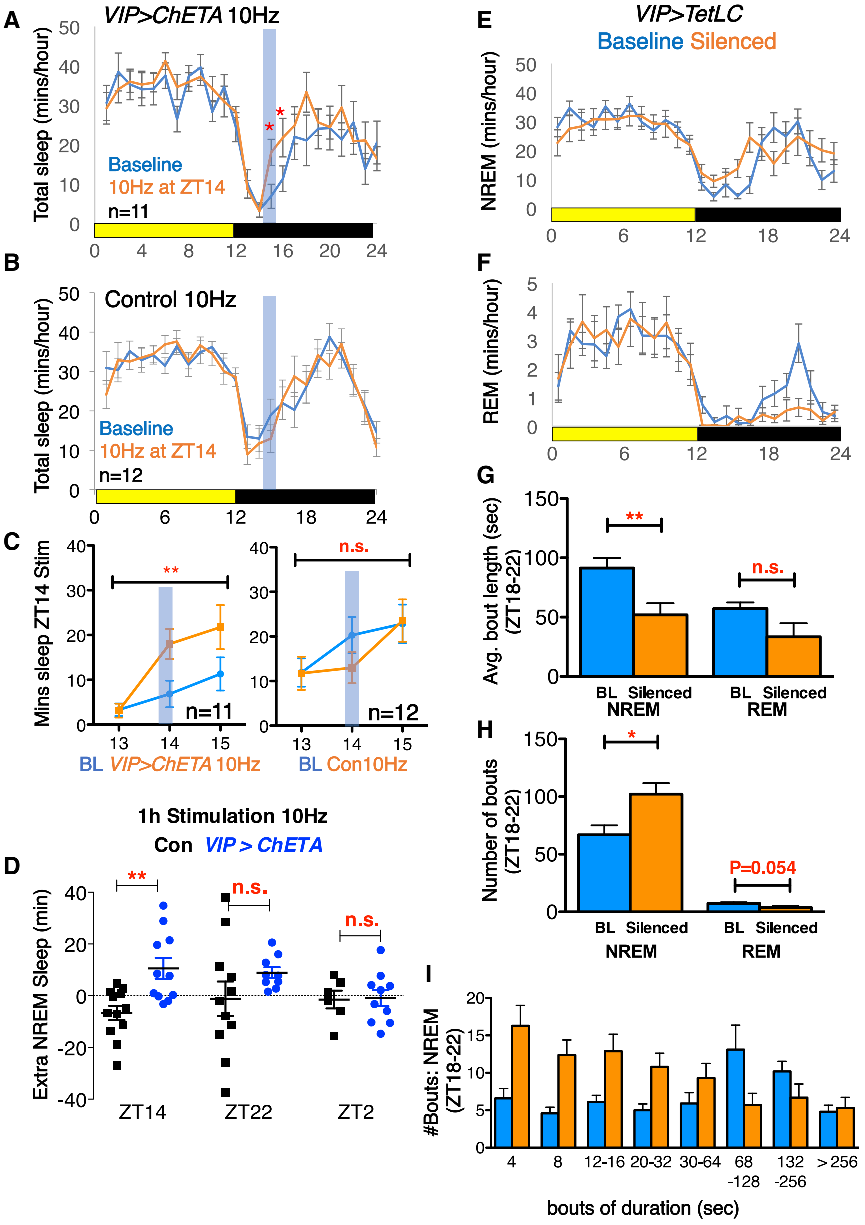

Figure 4. VIP+SCN Neuronal Activity Regulates Nighttime Sleep

(A) Total sleep as minutes per hour in 1 h bins in *VIP>ChETA* mice on the day of stimulation of VIP+SCN neurons at ZT14 (orange line) compared to baseline (blue line). Stimulation for 1 h at 10 Hz is indicated at blue bar.

(B) Same experiment, but with control-injected mice.

(C) Statistical summary before, during, and after stimulation. Left: *VIP>ChETA*; two-way ANOVA, $F(1, 60) = 7.373$, $p < 0.01$. Right: control; $F(1, 66) = 0.4899$.

(D) The change in NREM sleep (NREM during stimulation − baseline NREM) is plotted for ZT14, ZT22, or ZT2 for control (black) and *VIP>ChETA* (blue). NREM is significantly increased in *VIP>-ChETA* mice at ZT14. All stimulations of *VIP>-ChETA* mice at ZT22 increased NREM sleep, with activation at 10 Hz at ZT22 reducing variability between mice. F test to compare variance: NREM sleep, $F(10, 8) = 12.26$, $p < 0.01$. There was no effect of VIP+SCN neuron activation on sleep at ZT2.

(E–I) Amount of NREM (E) and REM (F) sleep in *VIP>TetLC* mice at baseline prior to injection and post-silencing, in 1-h bins. Blue, baseline (BL), prior to silencing; orange, silenced. REM sleep is specifically reduced during the siesta; quantified in Figure S6. (G) Length of NREM and REM sleep bouts in *VIP>TetLC* mice at baseline and post-silencing. (H) Number of NREM and REM sleep bouts for same as in (G). (I) Distribution of bout lengths for same.

Error bars represent SEM. Statistical comparison by two-tailed Student's t test unless otherwise stated. $^{*}p < 0.05$; $^{**}p < 0.01$.

See also Figures S6 and S7.

473 nm at a frequency of 10 Hz for 1 h; optogenetic activation was verified by optogenetically stimulating SCN slices *in vitro* (Figure S5C). Similar stimulation conditions were previously reported to be sufficient to entrain the clock in constant darkness, using a slightly different version of the optogenetic channel rhodopsin (Jones et al., 2015). Optogenetic activation of VIP+SCN neurons for 1 h at 10 Hz at ZT14 caused a rapid and significant reduction in RW activity compared to baseline for the duration of stimulation (Figure 3D, blue arrow and bar), followed by a significant increase in RW activity ~4 h post-stimulation compared to baseline (green arrow in Figure 3D: see also Figure 7). By contrast, stimulation at a lower frequency of 5 Hz resulted in a non-significant decrease in activity (Figure S5A): we suggest that activating VIP+SCN neurons at 10 Hz (but not 5 Hz) mimics the high-frequency VIP neuron activation that rapidly entrains circadian locomotor rhythms (Mazuski et al., 2018). No inhibition of activity was observed in control mice stimulated at 10 Hz (Figure 3E). An analogous, significant cessation of activity was observed for the duration of stimulation at ZT22, during the second peak of RW activity (Figure 3F). By contrast, no effects of stimulation were observed at ZT2, when the mouse is already quiescent (Figure S5B). We conclude that activation of VIP+SCN neurons is sufficient to induce siesta-like quiescence in active mice.

Activation of VIP+SCN Neurons Increases Nighttime but Not Daytime Sleep

We next tested whether optogenetic activation of VIP+SCN neurons acutely affected sleep, using chronically implanted sleep electrodes to record electroencephalogram (EEG) and electromyogram (EMG) activity alongside RW activity; sleep data shown in Figure 4 are from the same cohort of mice as that

providing RW data in Figure 3. During the first peak of RW activity (encompassing ZT14), mice normally show their lowest daily levels of sleep, and many mice do not sleep at all. With 10 Hz stimulation of *VIP>ChETA* SCN neurons at ZT14, total sleep (minutes of rapid eye movement [REM] + non-rapid eye movement [NREM]) during this interval was significantly increased, as were individual levels of NREM and REM sleep (Figures 4A, 4C, and 4D; Figures S6D, S6H, and S6I). No increase in sleep was detected during identical stimulations of control-injected animals (Figures 4B, 4C, and S6D) or in the same *VIP>ChETA* mice stimulated at 5Hz (Figures S6A and S6E) or in *VIP>ChETA* mice stimulated at 10 Hz at different times of day (2nd peak of activity/ZT22, quiescence/ZT2: Figures 4A, 4C, and 4D; Figures S6B–S6D and S6F–S6I). Overall, a highly significant cumulative increase in NREM but not REM sleep was seen when mice were stimulated at ZT14 during the first RW activity peak, a trend toward increase was seen at ZT22 during the second RW activity peak, and no increase was seen during quiescence at ZT2 (Figure 4D; Figure S6I). As mouse sleep is polyphasic, even at ZT2 mice are not asleep 100% of the time. We therefore conclude that signals from VIP+SCN neurons promote sleep either only at specific times of day or only when mice are normally most active.

Silencing VIP+SCN Neurons Suppresses Nighttime but Not Daytime Sleep

Given that activation of VIP+SCN neurons both inhibits RW activity and promotes sleep, we tested whether *VIP>TetLC* mice show disrupted siesta sleep. We repeated the experiment described in Figure 3, injecting *Vip-CRE* mice with *AAV.Flex.TetLC* with sleep electrodes now implanted simultaneously with injection. We found a corresponding increase in the number of short NREM sleep bouts (<60 s) and reduction in the number of long NREM sleep bouts (>60 s) during the siesta after *VIP>TetLC*-induced silencing (ZT18–22; Figures 4E and 4G–4I). There was no effect on daytime sleep. Thus, blocking synaptic transmission from VIP+SCN neurons specifically increases siesta sleep fragmentation. Corresponding to this fragmentation, there was a dramatic reduction in REM sleep around the siesta (Figures 4F–4H, S7A, and S7B). It is likely that the reduction in the number of long NREM bouts decreases the likelihood that mice enter REM sleep, resulting in the reduction in levels of REM sleep observed. Consistent with this, the average number of REM sleep bouts during the siesta after VIP+SCN neuronal silencing is reduced, with some mice failing to enter REM sleep during this period (Figure 4H; Figure S7C). The effect of *VIP>TetLC* on REM sleep fits with previous reports of disruption of REM sleep in $vip^{-/-}$ knockout mice (Husse et al., 2011); however, VIP signaling itself likely remains intact in the presence of TetLC (described earlier).

VIP+SCN Neuronal Activity Gates Nighttime Sleep

VIP>TetLC constitutively blocks the activity of VIP+SCN neurons but only affects nighttime sleep. This suggests that the effects of VIP+SCN neurons are gated to nighttime (or siesta) sleep. To test this, we injected *Vip-CRE* mice with a CRE-dependent virus encoding the optogenetic tool eNpHR3.0, which blocks neuronal firing in response to light delivered at 532 nm. VIP+SCN neurons were then silenced in 4 h windows from ZT0–4, ZT8–12, and ZT18–22 (Figure 5). Blocking activity of VIP+SCN neurons from ZT0–4, when mice are inactive and mostly asleep, or ZT8–12 when mice are transitioning from quiescence to anticipate the onset of night, had no effect on RW activity (Figures 5A, 5B, and 5D) or sleep (Figures 5E and 5F). In contrast, optogenetic silencing of VIP+SCN neurons during the siesta (ZT18–22) caused a significant increase in RW activity (Figures 5C and 5D) and concordant decrease in siesta sleep (Figures 5G and 5H). This confirms that the nighttime activity of VIP+SCN neurons is both required for and specific to the regulation of nighttime sleep. Thus, VIP+SCN neuronal signals provide a time-dependent gate on nighttime activity and sleep.

The Molecular Clock of VIP+SCN Neuron Times the Daily Siesta

Time-dependent effects on behavior are often controlled by the circadian clock. Therefore, we tested whether the molecular clock within VIP+ neurons times the daily siesta using $CKI\varepsilon^{tau}$ mice (Meng et al., 2008) to change the period of either VIP or AVP neurons, measuring the effect on siesta timing. Heterozygous $CKI\varepsilon^{tau/+}$ mice have an ~22-h period. $\mathrm{CKI}\varepsilon^{\mathrm{tau}}$ sits between flox sites and is deleted in the presence of CRE, resulting in a null allele; a single copy of wild-type CKIε is sufficient to generate a normal 24 h period. Thus, a mouse heterozygous for $CKI\varepsilon^{tau}$ crossed to a CRE line will result in a mouse where CRE-expressing cells have a 24 h period and CRE-negative cells have a ~22 h period. Although both AVP-CRE and VIP-CRE are also expressed outside the SCN, RW rhythms depend on the SCN, so effects on RW activity are likely the result of SCN-specific manipulations. We generated *AVP>*$CKI\varepsilon^{tau/+}$ mice (24 h in AVP neurons/22 h in VIP SCN neurons) and *VIP>*$CKI\varepsilon^{tau/+}$ mice (22 h in AVP SCN neurons/24 h in VIP neurons). *AVP>*$CKI\varepsilon^{tau/+}$ mice in which VIP neurons have a short period have a siesta at ~ZT17, significantly earlier than controls, whereas *VIP>*$CKI\varepsilon^{tau/+}$ mice, with a 24 h period in VIP neurons, have a siesta at ~ZT20, similar to wild-type (Figures 6A and 6C). We conclude that the timing of the siesta is determined by the molecular clock within VIP+SCN neurons.

We also tested the effect of deleting a functional circadian clock in SCN neurons. BMAL1 is a core transcription factor at the heart of the molecular clock, and $Bmal1^{-/-}$ mice are arrhythmic. Using a floxed allele of *Bmal1* ($Bmal1^{fl/fl}$), *Bmal1* can be conditionally knocked out in specific groups of cells through expression of CRE recombinase (Storch et al., 2007). We generated mice carrying VIP-CRE and $Bmal1^{fl/fl}$ (*VIP>B-*$mal^{fl/fl}$), resulting in the deletion of *Bmal1* from all VIP-expressing cells. *VIP>*$Bmal1^{fl/fl}$ mice show increased activity during the siesta (Figures 6B and 6C), similarly to *VIP>TetLC* mice (Figure 3B). Consistent with this, SCN siesta neuronal activity is reduced in *VIP>*$Bmal^{fl/fl}$ mice compared to controls, as measured by MEA and c-FOS expression (Figure S4H). Thus, *VIP>*$Bmal1^{fl/fl}$, like *VIP>TetLC*, reduces neurotransmission from VIP+SCN neurons, resulting in a disruption of the daily siesta. These data also suggest that the transcriptional state of the molecular SCN clock regulates neuronal activity. *VIP>*$Bmal1^{fl/fl}$ mice also show reduced RW activity at the beginning of the night, a phenotype not observed in *VIP>TetLC* mice. Therefore, loss of a functional clock in VIP+ neurons likely affects pathways

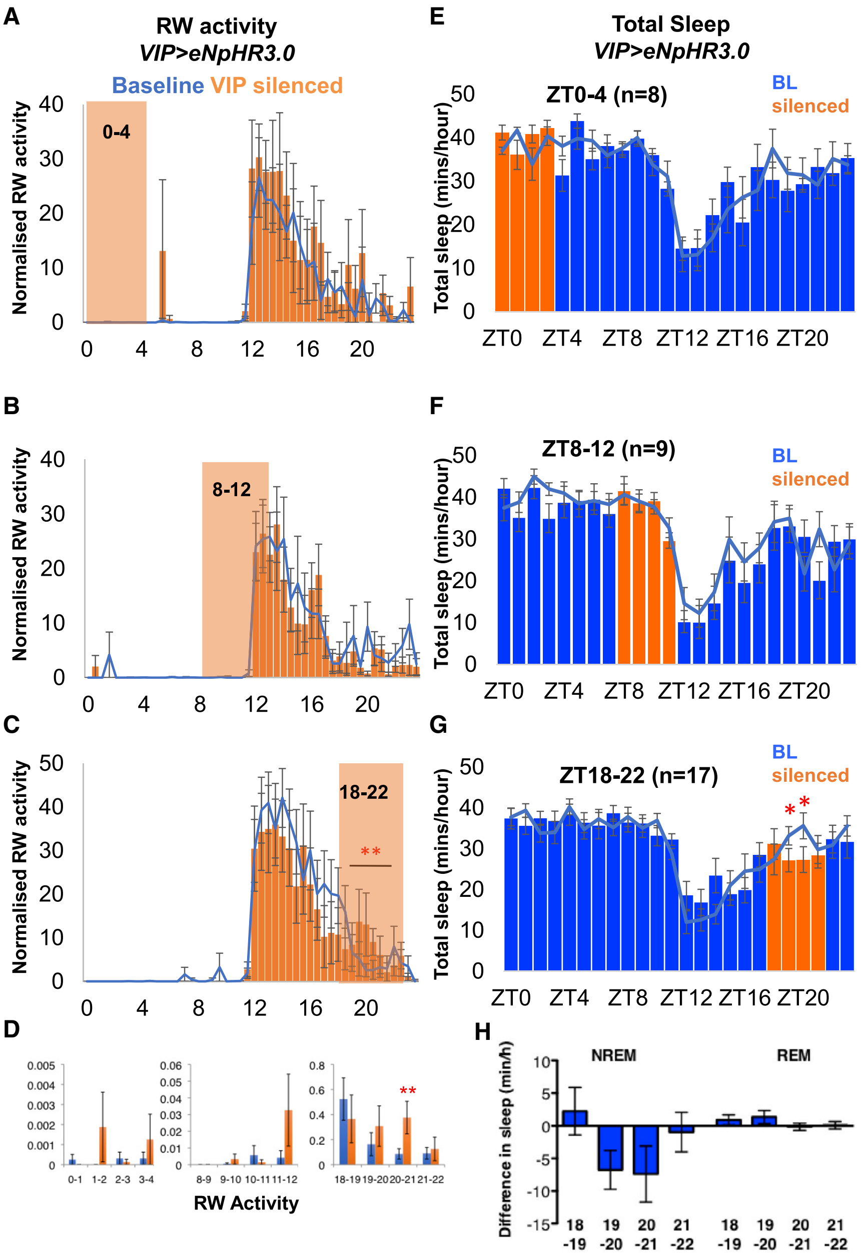

Figure 5. VIP+SCN Activity Regulates RW Activity and Sleep Only at Night

(A–D) Normalized RW activity in 30 min bins in *VIP>eNpHR3.0* mice on the day of silencing of VIP+SCN neurons (orange bars) compared to baseline (blue line). VIP+SCN neurons were optogenetically silenced for 4 h in 2 min cycles of 1 min on, 1 min off (orange box). Silencing from (A) ZT0–4 (n = 5), (B) ZT8–12 (n = 8), and (C) ZT18–22 (n = 8). (D) Statistical summary showing a significant effect on RW activity only during silencing from ZT18–22.

(E–H) Total sleep in 1 h bins in *VIP>eNpHR3.0* mice on the day of silencing of VIP+SCN neurons for 4 h in 2 min on/off cycles (blue bars; 4 h silencing is indicated with orange bars) compared to baseline (blue line). (E) ZT0–4. (F) ZT8-12. (G) ZT18–22. (H) The difference in NREM and REM sleep for each individual *VIP>eNpHR3.0* mouse between baseline and during 4 h silencing is shown for ZT18–22. Error bars represent SEM. Statistical comparison by two-tailed Student's t test. *p < 0.05; **p < 0.01.

beyond those blocked by TetLC expression that are important at other times of day. Although we cannot rule out potential developmental defects caused by the constitutive removal of *Bmal1* from VIP+ neurons or expression of $CKI\varepsilon^{tau}$ within VIP+ or AVP+ neurons, this is not something that has been reported in any previous studies.

Nighttime VIP+ Neuronal Firing Creates a Subsequent Circadian WMZ

We have demonstrated a clear causal relationship between firing of nighttime-active VIP+ neurons and a circadian clock-gated increase in siesta sleep. However, the physiological rationale for a clock-controlled siesta remains unclear. One function of sleep is to boost subsequent wakefulness, so we hypothesized that a pre-programmed siesta controlled by VIP+SCN neurons could increase alertness immediately afterward, at the end of the activity period. This end-of-activity wakefulness has been called the WMZ and has been extensively studied in humans (Zeeuw et al., 2018). To test the hypothesis that VIP+-driven nighttime sleep affects the WMZ, we re-examined our experiments to analyze sleep patterns in the hours following normal and (opto) genetically manipulated siesta sleep.

(1) Activation of VIP+SCN neurons for 1 h at ZT14 (but not ZT2 or ZT22) causes an immediate cessation of activity and increase in NREM and REM sleep (Figures 3D, 4A,

4D, S6D, S6H, and S6I), followed by a burst of RW activity from ~ZT18.5–19.5 that resembles the peak of activity that normally marks the end of the siesta (Figure 7A), effectively moving WMZ-like behavior earlier to follow the premature siesta.

(2) Moving nighttime sleep earlier genetically via *CKIε*^{tau} (Figure 6A) also advances end-of-night activity (Figure 7B).
(3) More generally, higher levels of siesta sleep are associated with reduced sleep during the WMZ (Figure 7C), with silencing of VIP+SCN neurons disrupting this equilibrium (Figures 7D and 7E). Analogously, optogenetically stimulating sleep at ZT14 provokes a corresponding reduction in sleep at ZT18, effectively a new, artificial "circadian WMZ" (Figure 7F). A model illustrating the role of VIP neurons in driving the daily siesta and subsequent WMZ is shown in Figure 7G.

DISCUSSION

We show that a nighttime-active population of VIP+ neurons "sculpts" sleep and activity in gated fashion—i.e., the same neurons driven or silenced at other times of day do not exert these effects; moreover, a clock in these neurons determines the timing of their principal effects. This is the first report of an acute effect of mammalian SCN neurons upon sleep, a starting point in understanding how circadian clocks control what is arguably their major behavioral output.

Previous data suggest that the SCN is primarily active during the day in both nocturnal and diurnal mammals (Challet, 2007). However, these results do not preclude nighttime activity of some SCN neurons. Although studies of calcium signaling from VIP+ neurons have not specifically reported nighttime activity (Jones et al., 2018; Mei et al., 2018), several previous studies identify night-active SCN neurons using c-FOS labeling (Yan and Silver, 2008), electrophysiology (Belle et al., 2009), and membrane voltage indicators (Enoki et al., 2017). Here, we identify ~20% of all SCN neurons as "night active" by c-FOS staining; our single-cell sequencing suggests that these night-active SCN neurons are either *avp+ nms+*, and active during the early night (to ~ZT16) or *vip+ nms+* and active during the daily siesta toward the end of the night. *Nms+* neurons represent most SCN neurons and are essential for rhythmicity (Lee et al., 2015). By specifically targeting our optogenetic/chemogenetic experiments to VIP+ or AVP+SCN neurons, we were able to determine that the VIPergic, night-active *nms+* population of SCN neurons is both necessary and sufficient to drive and time activity and sleep around the nighttime siesta, consistent with their phase of electrical activity. Using multiple bioinformatics tools, we have profiled gene ontologies unique to *vip+ nms+* neurons

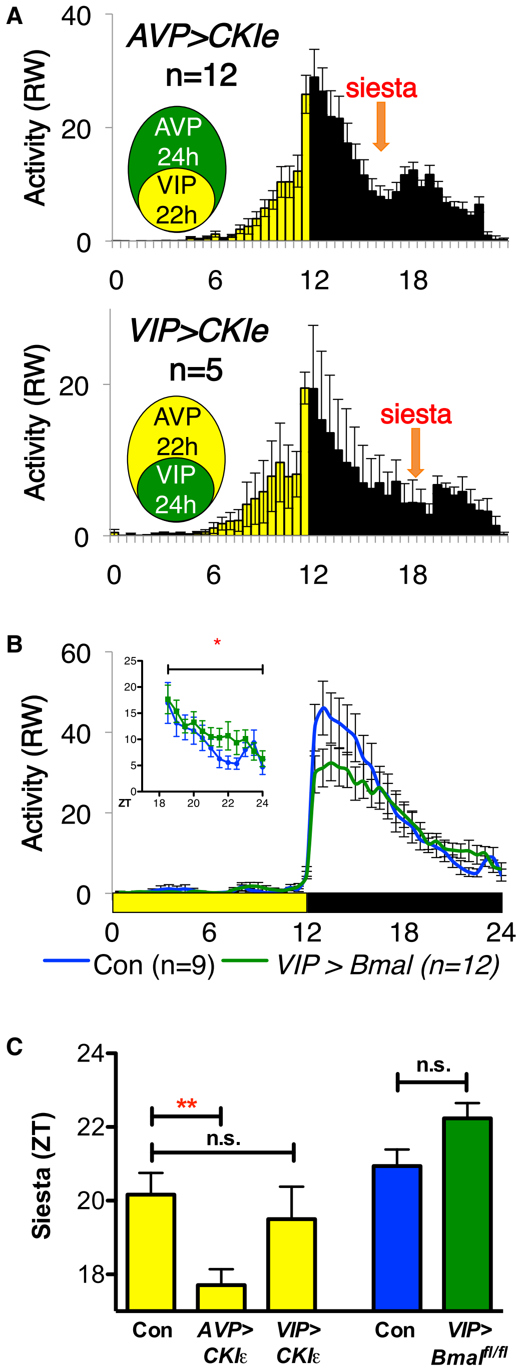

Figure 6. The Timing of the Siesta Is Regulated by the Molecular Clock

RW activity was plotted in 30-min bins. Yellow:black bars represent 12 h:12 h LD cycles. Normalized RW activity is plotted in 30 min bins.

(A) The *tau* allele of CKIe was used to shorten the period of VIP or AVP neurons. Top: *AVP>CKIe*^{tau} mice have a 22 h period in VIP neurons, 24 h in AVP neurons, and an advance in the timing of the siesta (orange arrow). Bottom: *VIP>CKIe*$^{tau\ mice}$ with a 22 h period in AVP neurons but 24 h in VIP neurons have normal siesta timing (orange arrow).

(B) *VIP > Bmal*$^{fl/fl}$ mice lacking a functional clock in all VIP-CRE-expressing cells show increased RW activity during the siesta compared to sibling controls, $F(1, 228) = 5.052$, $p < 0.05$.

(C) Quantification of the effect of VIP+SCN neurons upon siesta timing, defined as the point of lowest activity during the siesta. Comparisons by ANOVA with Tukey's multiple comparison test for *CKIε*: $F(2, 20) = 5.554$, $p = 0.0121$ (*Bmal*$^{fl/fl}$ by Student's t test).

Statistical comparisons as specified. Error bars represent SEM. *$p < 0.05$; **$p < 0.01$.

See also Figure S3.

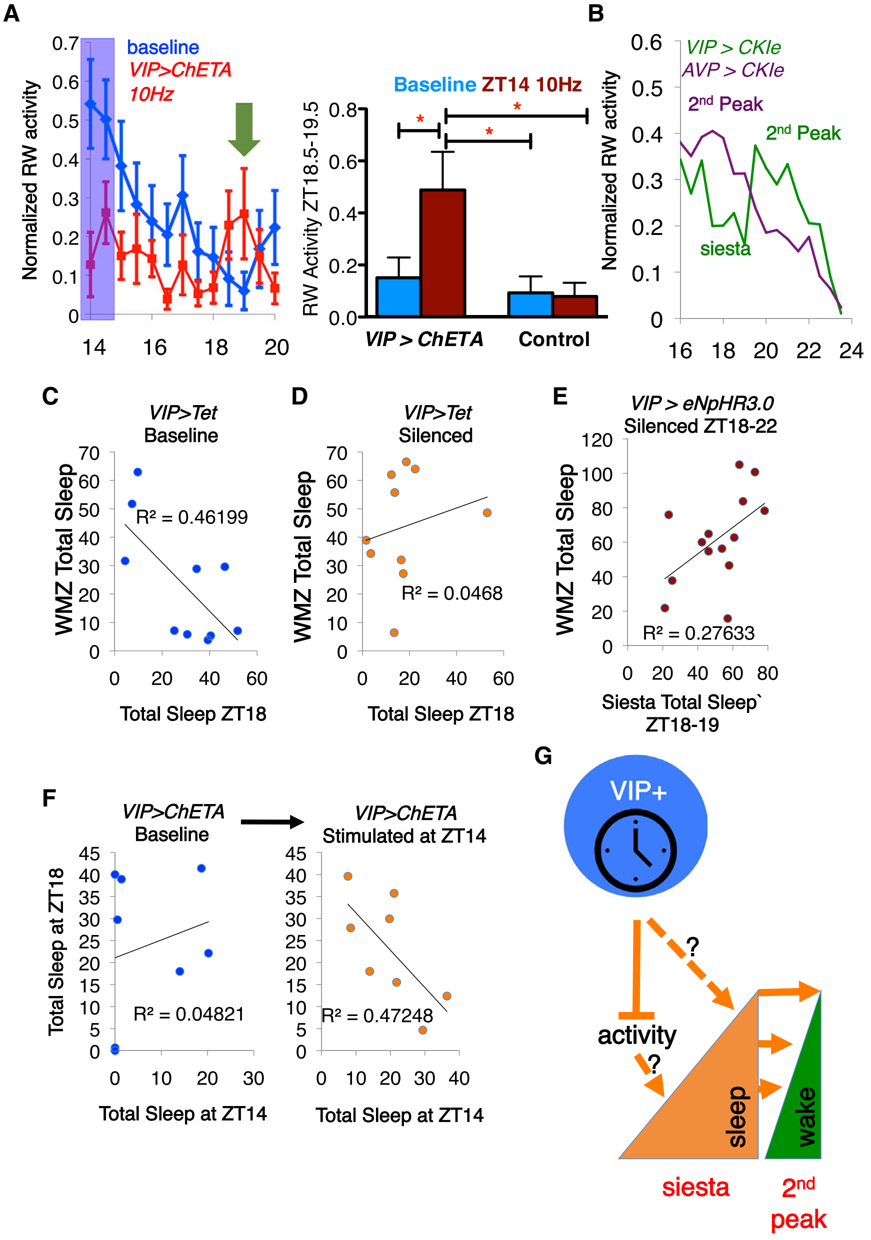

Figure 7. VIP+SCN Neuron-Driven Siesta Sleep Promotes Wake in the WMZ

(A) Left: average RW activity of baseline (blue) and *VIP>ChETA* (red) is re-plotted from Figure 3D to show increased RW activity in mice ~4–5 h post-stimulation (green arrow). Blue box represents 1-h stimulation. Right: statistical analysis of same; RW activity is significantly increased from ZT18.5–19.5 in *VIP>ChETA* mice after induction of a "siesta" through stimulation at 10 Hz at ZT14, compared to baseline or control mice after same stimulation (one-way ANOVA).

(B) RW activity of *AVP>CK1e* (purple) and *VIP>CKIe* (green) mice from Figure 6 re-plotted as normalized data to show the shift in timing of the second peak in *AVP>CKIe* mice.

(C and D) Correlation between total amount of sleep in *VIP>Tet* mice at ZT18 and during the WMZ (ZT23–24), at baseline (C) and post-silencing (D). Note significant negative correlation at baseline, $F(1, 8) = 6.87$, $p < 0.05$; which is lost after silencing VIP+SCN neurons, $F(1, 8) = 0.3928$, n.s. Data are from Figure 3.

(E) There is no significant correlation between siesta and WMZ sleep in *VIP>eNpHR3.0* mice upon optogenetic silencing from ZT18–22, $F(1, 14) = 4.58$, n.s. Data are re-plotted from 14/18 mice in Figure 4 that showed reduced siesta sleep from ZT19–20 during silencing.

(F) Correlation between total amount of sleep in *VIP>ChETA* mice at ZT14 at baseline (left) or during 10 Hz stimulation (right) with sleep at ZT18. Note negative correlation between total sleep at ZT14 and sleep at ZT18 post-stimulation (data are from Figure 5); $F(1, 5) = 6.771$, $p < 0.05$; no correlation without stimulation, $F(1, 5) = 0.4305$, n.s.

(G) Model for the generation of siesta sleep and promotion of wakefulness in the wake maintenance zone (WMZ). VIP+SCN neurons signal at night to inhibit locomotor activity and/or directly promote sleep. As sleep accumulates during the siesta, consolidated by signals from VIP neurons, the amount of sleep required during the 2nd peak of RW activity (WMZ) is reduced. Thus, we propose that VIP+SCN neurons promote activity during the WMZ by increasing sleep during the siesta.

Statistical comparisons are as specified. Error bars represent SEM. *$p < 0.05$.

(Figure S3). Beyond expression of *vip* itself, these tools depict changes in gene expression within the G-protein coupled receptor (GPCR) and adenyl cyclase signaling pathways, consistent with previously reported ERK1/2 signaling in response to VIP within the SCN (Hamnett et al., 2019). Also overrepresented are genes involved in the cellular response to light, suggesting that night-active *vip+ nms+* neurons generally resemble neurons activated by light, even if no light stimulus occurred. Finally, upregulated genes within these pathways are also in common with a mouse circadian hyperactivity disorder model (Hagihara et al., 2016).

In normal LD cycles, VIP+SCN neurons have been shown to be important for changing circadian time (phase shifting) (Jones et al., 2018) and participate in neuropeptide-mediated coupling of SCN subregions (Maywood et al., 2011) (Hamnett et al., 2019). Because of their acute effects in silencing mouse activity, they have also been postulated to be essential to "masking," the acute inhibition of rodent activity by light (Mazuski et al., 2018). Deletion of the gene encoding VIP silences SCN electrical activity (Brown et al., 2007), while high-frequency firing of VIP+-SCN neurons is required for setting clock phase and behavior in a VIP-dependent manner (Mazuski et al., 2018). In circadian daytime, VIP neuronal signaling provides GABAergic inhibition to neighboring hypothalamic and thalamic regions to regulate circadian heart rate and corticosterone (Paul et al., 2020). Thus, a robust literature links VIP neurons to circadian function.

We suggest that these neurons play an even wider role, essentially sculpting nighttime behavior by determining "siesta napping" and the timing of subsequent wakefulness. Our data suggest two mechanisms by which VIP+SCN neuron activation could promote siesta sleep. First, VIP+SCN neurons may inhibit activity and promote quiescence, allowing mice to fall asleep more easily in response to sleep pressure, similarly to how humans lie down in bed before going to sleep. Alternatively, it is possible that VIP+SCN signals directly promote sleep and/or encourage fatigue.

For either mechanism, we find that a strict circadian gating occurs: firing or silencing these neurons has dramatic effects upon only nighttime activity and sleep. Even though mouse sleep is polyphasic, with periods of wake and sleep across the day and night, there is no discernible effect upon sleep of either activating or silencing VIP+SCN during the day. In this respect, the mechanism by which the SCN sculpts nighttime behavior is surprisingly similar to the *Drosophila* circadian clock circuit, in which a specific population of small ventral lateral neurons expressing the neuropeptide PDF are required for the M peak of activity anticipating dawn, while a subset of the remaining PDF− clock neurons (E cells) are required to anticipate dusk (Grima et al., 2004; Stoleru et al., 2004). In this scheme, VIP+SCN neurons might roughly resemble fly DN_1 neurons that promote nighttime sleep by inhibiting activity-promoting E cells: these DN_1s are also required for a normal daily siesta (Guo et al., 2016). Since the *Drosophila* siesta varies significantly between genders, it would also be interesting to examine gender differences in these proposed mechanisms in both mice and flies.

It has been suggested that a siesta occurs because of rising sleep pressure after prolonged periods of wake (Ehlen et al., 2015; Owens et al., 2010; Reichert et al., 2014). Even though a siesta is nonessential and genetically variable in mice (Franken et al., 2001), *Drosophila* (Ehlen et al., 2013)), and humans (Lopez-Minguez et al., 2017), our results suggest its specific utility: to maintain alertness at the end of the activity period. Across species, a period of increased activity anticipating dark:light transitions could be useful both metabolically as a last opportunity to forage before sleeping and to avoid risk of crepuscular predation. A programmed daily siesta could also aid entrainment to changing seasonal conditions by providing a mechanism by which an organism extends its active period; indeed, in rats, napping is highly dependent upon photoperiod, with longer nights creating more pronounced naps (Franken et al., 1995). In humans, a better understanding of the mechanisms underlying our own well-documented mid-afternoon fatigue and early evening WMZ (Lack and Wright, 2007) could provide an important intervention point to improve sleep consolidation.

STAR★METHODS

Detailed methods are provided in the online version of this paper and include the following:

- KEY RESOURCES TABLE
- RESOURCE AVAILABILITY
  - Lead Contact
  - Materials Availability
  - Data and Code Availability
- EXPERIMENTAL MODEL AND SUBJECT DETAILS
  - Mouse strains and husbandry
- METHOD DETAILS
- QUANTIFICATION AND STATISTICAL ANALYSIS
  - Immunocytochemistry and cFOS quantification
  - Patch clamp and multi-electrode recordings
  - Patch-Seq
  - Surgical Procedures

SUPPLEMENTAL INFORMATION

Supplemental Information can be found online at https://doi.org/10.1016/j.neuron.2020.08.001.

ACKNOWLEDGMENTS

Special thanks to Prof. Hanns Ulrich Zeilhofer and Dr. Laetitia Tudeau for equipment and assistance with electrophysiology; to Dr. Hendrik Wilder for AAV.Flex.TetLC virus; to the UZH Vector Core and the UNC Vector Core for viral production; and to Prof. K. Deisseroth for permission to use the optogenetic virus. This work was supported by the Velux Foundation, the UZH Clinical Research Priority Project "Sleep and Health," the Swiss National Science Foundation, the Borbely-Hess Foundation, and the Human Frontiers Science Program. Zürich researchers are members of the Zürich Neurozentrum and the Life Sciences Zürich graduate program.

AUTHOR CONTRIBUTIONS

This work was conceived by B.C. and S.A.B. Experiments were performed by B.C., S.P.-F., C.M., and A.S., with guidance from A.A., C.G.H., J.W., and C.F. M.H. and A.L. contributed mice. M.D.C.B. and H.D.P. advised on MEA. D.L. contributed to bioinformatical analysis. Y.C., S.W., and J.Y. provided Drop-seq data and identified SCN cell types. Data analysis was performed by B.C. and S.P.-F., and the manuscript was written by B.C., S.P.-F., and S.A.B.

DECLARATION OF INTERESTS

The authors declare no competing interests.

Received: June 30, 2019
Revised: October 7, 2019
Accepted: July 31, 2020
Published: September 10, 2020

REFERENCES

An, S., Irwin, R.P., Allen, C.N., Tsai, C., and Herzog, E.D. (2011). Vasoactive intestinal polypeptide requires parallel changes in adenylate cyclase and phospholipase C to entrain circadian rhythms to a predictable phase. J. Neurophysiol. *105*, 2289–2296.

Aton, S.J., and Herzog, E.D. (2005). Come together, right...now: synchronization of rhythms in a mammalian circadian clock. Neuron *48*, 531–534.

Anders, S., Pyl, P.T., and Huber, W. (2015). HTSeq—a Python framework to work with high-throughput sequencing data. Bioinformatics *31*, 166–169.

Aton, S.J., Colwell, C.S., Harmar, A.J., Waschek, J., and Herzog, E.D. (2005). Vasoactive intestinal polypeptide mediates circadian rhythmicity and synchrony in mammalian clock neurons. Nat. Neurosci. *8*, 476–483.

Aton, S.J., Huettner, J.E., Straume, M., and Herzog, E.D. (2006). GABA and Gi/o differentially control circadian rhythms and synchrony in clock neurons. Proc. Natl. Acad. Sci. USA *103*, 19188–19193.

Azzi, A., Dallmann, R., Casserly, A., Rehrauer, H., Patrignani, A., Maier, B., Kramer, A., and Brown, S.A. (2014). Circadian behavior is light-reprogrammed by plastic DNA methylation. Nat. Neurosci. *17*, 377–382.

Azzi, A., Evans, J.A., Leise, T., Myung, J., Takumi, T., Davidson, A.J., and Brown, S.A. (2017). Network Dynamics Mediate Circadian Clock Plasticity. Neuron *93*, 441–450.

Belle, M.D., Diekman, C.O., Forger, D.B., and Piggins, H.D. (2009). Daily electrical silencing in the mammalian circadian clock. Science *326*, 281–284.

Bolger, A.M., Lohse, M., and Usadel, B. (2014). Trimmomatic: a flexible trimmer for Illumina sequence data. Bioinformatics *30*, 2114–2120.

Borbély, A.A. (1982). A two process model of sleep regulation. Hum. Neurobiol. *1*, 195–204.

Brancaccio, M., Maywood, E.S., Chesham, J.E., Loudon, A.S., and Hastings, M.H. (2013). A Gq-Ca2+ axis controls circuit-level encoding of circadian time in the suprachiasmatic nucleus. Neuron *78*, 714–728.

Brown, S.A., and Azzi, A. (2013). Peripheral circadian oscillators in mammals. Handb. Exp. Pharmacol. (217), 45–66.

Brown, T.M., Colwell, C.S., Waschek, J.A., and Piggins, H.D. (2007). Disrupted neuronal activity rhythms in the suprachiasmatic nuclei of vasoactive intestinal polypeptide-deficient mice. J. Neurophysiol. *97*, 2553–2558.

Butler, A., Hoffman, P., Smibert, P., Papalexi, E., and Satija, R. (2018). Integrating single-cell transcriptomic data across different conditions, technologies, and species. Nat. Biotechnol. *36*, 411–420.

Challet, E. (2007). Minireview: Entrainment of the suprachiasmatic clockwork in diurnal and nocturnal mammals. Endocrinology *148*, 5648–5655.

Colwell, C.S. (2011). Linking neural activity and molecular oscillations in the SCN. Nat. Rev. Neurosci. *12*, 553–569.

Deboer, T., Franken, P., and Tobler, I. (1994). Sleep and cortical temperature in the Djungarian hamster under baseline conditions and after sleep deprivation. J. Comp. Physiol. A Neuroethol. Sens. Neural Behav. Physiol. *174*, 145–155.

Dobin, A., Davis, C.A., Schlesinger, F., Drenkow, J., Zaleski, C., Jha, S., Batut, P., Chaisson, M., and Gingeras, T.R. (2013). STAR: ultrafast universal RNA-seq aligner. Bioinformatics *29*, 15–21.

Dodt, M., Roehr, J.T., Ahmed, R., and Dieterich, C. (2012). Flexbar—flexible barcode and adapter processing for next-generation sequencing platforms. Biology (Basel) *1*, 895–905.

Easton, A., Meerlo, P., Bergmann, B., and Turek, F.W. (2004). The suprachiasmatic nucleus regulates sleep timing and amount in mice. Sleep *27*, 1307–1318.

Ehlen, J.C., Hesse, S., Pinckney, L., and Paul, K.N. (2013). Sex chromosomes regulate nighttime sleep propensity during recovery from sleep loss in mice. PLoS ONE *8*, e62205.

Ehlen, J.C., Jones, K.A., Pinckney, L., Gray, C.L., Burette, S., Weinberg, R.J., Evans, J.A., Brager, A.J., Zylka, M.J., Paul, K.N., et al. (2015). Maternal Ube3a Loss Disrupts Sleep Homeostasis But Leaves Circadian Rhythmicity Largely Intact. J. Neurosci. *35*, 13587–13598.

Enoki, R., Oda, Y., Mieda, M., Ono, D., Honma, S., and Honma, K.I. (2017). Synchronous circadian voltage rhythms with asynchronous calcium rhythms in the suprachiasmatic nucleus. Proc. Natl. Acad. Sci. USA *114*, E2476–E2485.

Evans, J.A., Leise, T.L., Castanon-Cervantes, O., and Davidson, A.J. (2013). Dynamic interactions mediated by nonredundant signaling mechanisms couple circadian clock neurons. Neuron *80*, 973–983.

Földy, C., Darmanis, S., Aoto, J., Malenka, R.C., Quake, S.R., and Südhof, T.C. (2016). Single-cell RNAseq reveals cell adhesion molecule profiles in electrophysiologically defined neurons. Proc. Natl. Acad. Sci. USA *113*, E5222–E5231.

Foster, E., Wildner, H., Tudeau, L., Haueter, S., Ralvenius, W.T., Jegen, M., Johannssen, H., Hösli, L., Haenraets, K., Ghanem, A., et al. (2015). Targeted ablation, silencing, and activation establish glycinergic dorsal horn neurons as key components of a spinal gate for pain and itch. Neuron *85*, 1289–1304.

Franken, P., Dijk, D.J., Tobler, I., and Borbély, A.A. (1994). High-frequency components of the rat electrocorticogram are modulated by the vigilance states. Neurosci. Lett. *167*, 89–92.

Franken, P., Tobler, I., and Borbély, A.A. (1995). Varying photoperiod in the laboratory rat: profound effect on 24-h sleep pattern but no effect on sleep homeostasis. Am. J. Physiol. *269*, R691–R701.

Franken, P., Chollet, D., and Tafti, M. (2001). The homeostatic regulation of sleep need is under genetic control. J. Neurosci. *21*, 2610–2621.

Freeman, G.M., Jr., Krock, R.M., Aton, S.J., Thaben, P., and Herzog, E.D. (2013). GABA networks destabilize genetic oscillations in the circadian pacemaker. Neuron *78*, 799–806.

Gizowski, C., Zaelzer, C., and Bourque, C.W. (2016). Clock-driven vasopressin neurotransmission mediates anticipatory thirst prior to sleep. Nature *537*, 685–688.

Grima, B., Chélot, E., Xia, R., and Rouyer, F. (2004). Morning and evening peaks of activity rely on different clock neurons of the *Drosophila* brain. Nature *431*, 869–873.

Gunaydin, L.A., Yizhar, O., Berndt, A., Sohal, V.S., Deisseroth, K., and Hegemann, P. (2010). Ultrafast optogenetic control. Nat. Neurosci. *13*, 387–392.

Guo, F., Yu, J., Jung, H.J., Abruzzi, K.C., Luo, W., Griffith, L.C., and Rosbash, M. (2016). Circadian neuron feedback controls the Drosophila sleep–activity profile. Nature *536*, 292–297.

Hagihara, H., Horikawa, T., Nakamura, H.K., Umemori, J., Shoji, H., Kamitani, Y., and Miyakawa, T. (2016). Circadian Gene Circuitry Predicts Hyperactive Behavior in a Mood Disorder Mouse Model. Cell Rep. *14*, 2784–2796.

Hamnett, R., Crosby, P., Chesham, J.E., and Hastings, M.H. (2019). Vasoactive intestinal peptide controls the suprachiasmatic circadian clock network via ERK1/2 and DUSP4 signalling. Nat. Commun. *10*, 542.

Harmar, A.J., Marston, H.M., Shen, S., Spratt, C., West, K.M., Sheward, W.J., Morrison, C.F., Dorin, J.R., Piggins, H.D., Reubi, J.C., et al. (2002). The VPAC(2) receptor is essential for circadian function in the mouse suprachiasmatic nuclei. Cell *109*, 497–508.

Harris, J.A., Hirokawa, K.E., Sorensen, S.A., Gu, H., Mills, M., Ng, L.L., Bohn, P., Mortrud, M., Ouellette, B., Kidney, J., et al. (2014). Anatomical characterization of Cre driver mice for neural circuit mapping and manipulation. Front. Neural Circuits *8*, 76.

Houben, T., Coomans, C.P., and Meijer, J.H. (2014). Regulation of circadian and acute activity levels by the murine suprachiasmatic nuclei. PLoS ONE *9*, e110172.

Husse, J., Zhou, X., Shostak, A., Oster, H., and Eichele, G. (2011). Synaptotagmin10-Cre, a driver to disrupt clock genes in the SCN. J. Biol. Rhythms *26*, 379–389.

Jones, J.R., Tackenberg, M.C., and McMahon, D.G. (2015). Manipulating circadian clock neuron firing rate resets molecular circadian rhythms and behavior. Nat. Neurosci. *18*, 373–375.

Jones, J.R., Simon, T., Lones, L., and Herzog, E.D. (2018). SCN VIP Neurons Are Essential for Normal Light-Mediated Resetting of the Circadian System. J. Neurosci. *38*, 7986–7995.

Lack, L.C., and Wright, H.R. (2007). Clinical management of delayed sleep phase disorder. Behav. Sleep Med. *5*, 57–76.

Lee, M.L., Swanson, B.E., and de la Iglesia, H.O. (2009). Circadian timing of REM sleep is coupled to an oscillator within the dorsomedial suprachiasmatic nucleus. Curr. Biol. *19*, 848–852.

Lee, I.T., Chang, A.S., Manandhar, M., Shan, Y., Fan, J., Izumo, M., Ikeda, Y., Motoike, T., Dixon, S., Seinfeld, J.E., et al. (2015). Neuromedin s-producing neurons act as essential pacemakers in the suprachiasmatic nucleus to couple clock neurons and dictate circadian rhythms. Neuron *85*, 1086–1102.

Li, H., Handsaker, B., Wysoker, A., Fennell, T., Ruan, J., Homer, N., Marth, G., Abecasis, G., and Durbin, R.; 1000 Genome Project Data Processing Subgroup (2009). The Sequence Alignment/Map format and SAMtools. Bioinformatics *25*, 2078–2079.

Liang, X., Holy, T.E., and Taghert, P.H. (2016). Synchronous *Drosophila* circadian pacemakers display nonsynchronous Ca^{2+} rhythms in vivo. Science *351*, 976–981.

CellPress

Lopez-Minguez, J., Morosoli, J.J., Madrid, J.A., Garaulet, M., and Ordoñana, J.R. (2017). Heritability of siesta and night-time sleep as continuously assessed by a circadian-related integrated measure. Sci. Rep. *7*, 12340.

Love, M.I., Huber, W., and Anders, S. (2014). Moderated estimation of fold change and dispersion for RNA-seq data with DESeq2. Genome Biol. *15*, 550.

Lun, A.T., McCarthy, D.J., and Marioni, J.C. (2016). A step-by-step workflow for low-level analysis of single-cell RNA-seq data with Bioconductor. F1000Res. *5*, 2122.

Maywood, E.S., Reddy, A.B., Wong, G.K., O'Neill, J.S., O'Brien, J.A., McMahon, D.G., Harmar, A.J., Okamura, H., and Hastings, M.H. (2006). Synchronization and maintenance of timekeeping in suprachiasmatic circadian clock cells by neuropeptidergic signaling. Curr. Biol. *16*, 599–605.

Maywood, E.S., Chesham, J.E., O'Brien, J.A., and Hastings, M.H. (2011). A diversity of paracrine signals sustains molecular circadian cycling in suprachiasmatic nucleus circuits. Proc. Natl. Acad. Sci. USA *108*, 14306–14311.

Mazuski, C., Abel, J.H., Chen, S.P., Hermanstyne, T.O., Jones, J.R., Simon, T., Doyle, F.J., 3rd, and Herzog, E.D. (2018). Entrainment of Circadian Rhythms Depends on Firing Rates and Neuropeptide Release of VIP SCN Neurons. Neuron *99*, 555–563.e5.

Mei, L., Zhan, C., and Zhang, E.E. (2018). In Vivo Monitoring of Circadian Clock Gene Expression in the Mouse Suprachiasmatic Nucleus Using Fluorescence Reporters. J. Vis. Exp. *137*, 56765.

Meng, Q.J., Logunova, L., Maywood, E.S., Gallego, M., Lebiecki, J., Brown, T.M., Sládek, M., Semikhodskii, A.S., Glossop, N.R.J., Piggins, H.D., et al. (2008). Setting clock speed in mammals: the CK1 epsilon tau mutation in mice accelerates circadian pacemakers by selectively destabilizing PERIOD proteins. Neuron *58*, 78–88.

Miladinović, Đ., Muheim, C., Bauer, S., Spinnler, A., Noain, D., Bandarabadi, M., Gallusser, B., Krummenacher, G., Baumann, C., Adamantidis, A., et al. (2019). SPINDLE: End-to-end learning from EEG/EMG to extrapolate animal sleep scoring across experimental settings, labs and species. PLoS Comput. Biol. *15*, e1006968.

Mootha, V.K., Lindgren, C.M., Eriksson, K.F., Subramanian, A., Sihag, S., Lehar, J., Puigserver, P., Carlsson, E., Ridderstråle, M., Laurila, E., et al. (2003). PGC-1alpha-responsive genes involved in oxidative phosphorylation are coordinately downregulated in human diabetes. Nat. Genet. *34*, 267–273.

O'Neill, J.S., Maywood, E.S., Chesham, J.E., Takahashi, J.S., and Hastings, M.H. (2008). cAMP-dependent signaling as a core component of the mammalian circadian pacemaker. Science *320*, 949–953.

Owens, J.F., Buysse, D.J., Hall, M., Kamarck, T.W., Lee, L., Strollo, P.J., Reis, S.E., and Matthews, K.A. (2010). Napping, nighttime sleep, and cardiovascular risk factors in mid-life adults. J. Clin. Sleep Med. *6*, 330–335.

Palchykova, S., Winsky-Sommerer, R., Shen, H.Y., Boison, D., Gerling, A., and Tobler, I. (2010). Manipulation of adenosine kinase affects sleep regulation in mice. J. Neurosci. *30*, 13157–13165.

Parisky, K.M., Agosto, J., Pulver, S.R., Shang, Y., Kuklin, E., Hodge, J.J., Kang, K., Liu, X., Garrity, P.A., Rosbash, M., and Griffith, L.C. (2008). PDF cells are a GABA-responsive wake-promoting component of the *Drosophila* sleep circuit. Neuron *60*, 672–682.

Paul, S., Hanna, L., Harding, C., Hayter, E.A., Walmsley, L., Bechtold, D.A., and Brown, T.M. (2020). Output from VIP cells of the mammalian central clock regulates daily physiological rhythms. Nat. Commun. *11*, 1453.

Pittendrigh, C., and Daan, S. (1976). A functional analysis of circadian pacemakers in nocturnal rodents. V. Pacemaker structure: a clock for all seasons. J. Comp. Physiol. A Neuroethol. Sens. Neural Behav. Physiol. *106*, 333–355.

Reichert, C.F., Maire, M., Gabel, V., Viola, A.U., Kolodyazhniy, V., Strobel, W., Götz, T., Bachmann, V., Landolt, H.P., Cajochen, C., and Schmidt, C. (2014). Insights into behavioral vulnerability to differential sleep pressure and circadian phase from a functional ADA polymorphism. J. Biol. Rhythms *29*, 119–130.

Reijmers, L.G., Perkins, B.L., Matsuo, N., and Mayford, M. (2007). Localization of a stable neural correlate of associative memory. Science *317*, 1230–1233.

Risso, D., Perraudeau, F., Gribkova, S., Dudoit, S., and Vert, J.-P. (2018). A general and flexible method for signal extraction from single-cell RNA-seq data. Nat. Commun. *9*, 284.

Schilling, K., Luk, D., Morgan, J.I., and Curran, T. (1991). Regulation of a fos-lacZ fusion gene: a paradigm for quantitative analysis of stimulus-transcription coupling. Proc. Natl. Acad. Sci. USA *88*, 5665–5669.

Schwartz, W.J., and Zimmerman, P. (1991). Lesions of the suprachiasmatic nucleus disrupt circadian locomotor rhythms in the mouse. Physiol. Behav. *49*, 1283–1287.

Stoleru, D., Peng, Y., Agosto, J., and Rosbash, M. (2004). Coupled oscillators control morning and evening locomotor behaviour of *Drosophila*. Nature *431*, 862–868.

Storch, K.F., Paz, C., Signorovitch, J., Raviola, E., Pawlyk, B., Li, T., and Weitz, C.J. (2007). Intrinsic circadian clock of the mammalian retina: importance for retinal processing of visual information. Cell *130*, 730–741.

Stuart, T., Butler, A., Hoffman, P., Hafemeister, C., Papalexi, E., Mauck, W.M., III, Hao, Y., Stoeckius, M., Smibert, P., and Satija, R. (2019). Comprehensive Integration of Single-Cell Data. Cell *177*, 1888–1902.

Subramanian, A., Tamayo, P., Mootha, V.K., Mukherjee, S., Ebert, B.L., Gillette, M.A., Paulovich, A., Pomeroy, S.L., Golub, T.R., Lander, E.S., and Mesirov, J.P. (2005). Gene set enrichment analysis: a knowledge-based approach for interpreting genome-wide expression profiles. Proc. Natl. Acad. Sci. USA *102*, 15545–15550.

Sun, S., Xu, Q., Guo, C., Guan, Y., Liu, Q., and Dong, X. (2017). Leaky Gate Model: Intensity-Dependent Coding of Pain and Itch in the Spinal Cord. Neuron *93*, 840–853.e5.

Taniguchi, H., He, M., Wu, P., Kim, S., Paik, R., Sugino, K., Kvitsiani, D., Fu, Y., Lu, J., Lin, Y., et al. (2011). A resource of Cre driver lines for genetic targeting of GABAergic neurons in cerebral cortex. Neuron *71*, 995–1013.

VanderLeest, H.T., Houben, T., Michel, S., Deboer, T., Albus, H., Vansteensel, M.J., Block, G.D., and Meijer, J.H. (2007). Seasonal encoding by the circadian pacemaker of the SCN. Curr. Biol. *17*, 468–473.

Varadarajan, S., Tajiri, M., Jain, R., Holt, R., Ahmed, Q., LeSauter, J., and Silver, R. (2018). Connectome of the Suprachiasmatic Nucleus: New Evidence of the Core-Shell Relationship. eNeuro *5*, ENEURO0205-18.2018.

Wen, S., Ma, D., Zhao, M., Xie, L., Wu, Q., Gou, L., Zhu, C., Fan, Y., Wang, H., and Yan, J. (2020). Spatiotemporal single-cell analysis of gene expression in the mouse suprachiasmatic nucleus. Nat. Neurosci. *23*, 456–467.

Yan, L., and Silver, R. (2008). Day-length encoding through tonic photic effects in the retinorecipient SCN region. Eur. J. Neurosci. *28*, 2108–2115.

Zeeuw, J., Wisniewski, S., Papakonstantinou, A., Bes, F., Wahnschaffe, A., Zaleska, M., Kunz, D., and Münch, M. (2018). The alerting effect of the wake maintenance zone during 40 hours of sleep deprivation. Sci. Rep. *8*, 11012.

STAR★METHODS

KEY RESOURCES TABLE

REAGENT or RESOURCE	SOURCE	IDENTIFIER
Antibodies		
mouse anti-c-Fos	Santa Cruz Biotechnologies	Cat#sc-166940; RRID: AB_10609634
rabbit anti-VIP	Peninsula Laboratories	Cat#T-4246.0050; RRID: AB_518682
chicken anti-GFP	Aves Labs	RRID: AB_2307313
Bacterial and Virus Strains		
AAV-EF1a-DIO-ChETA-EYFP	UNC Vector Core (Gunaydin et al., 2010)	N/A
AAV-EF1a-DIO-YFP	UNC Vector Core (Gunaydin et al., 2010)	N/A
AAV-EF1a-DIO-eNpHR3.0-EYFP	UNC Vector Core Gradinaru et al., 2010	N/A
AAV.Flex.TetLC	UZH Vector Core; Foster et al., 2015	N/A
Critical Commercial Assays		
SMART-Seq HT kit	Takara Bio	Cat#634438
Nextera XT DNA Sample Preparation Kit	Illumina	Cat#FC-131-1096
NextSeq 300 high-output kit	Illumina	Cat#20024908
Deposited Data		
Raw and analyzed data	This paper	Zenodo: 10.5281/zenodo.3946217
scRNAseq data	This paper	GEO: GSE154038
Experimental Models: Organisms/Strains		
VIP-CRE	Taniguchi et al., 2011	https://www.jax.org/strain/031628
AVP-CRE	Harris et al., 2014	https://www.jax.org/strain/023530
GRP-CRE	Sun et al., 2017	N/A
Syt10-CRE	Husse et al., 2011	N/A
CKIe^{tau}	Hastings lab; Meng et al., 2008	N/A
Bmal1$^{fl/fl}$	Storch et al., 2007	N/A
cFOS::GFP		https://www.jax.org/strain/018306
Software and Algorithms		
Trimmomatic	Bolger et al., 2014	http://www.usadellab.org/cms/index.php?page=trimmomatic; RRID: SCR_011848
Flexbar	Dodt et al., 2012	https://github.com/seqan/flexbar; RRID: SCR_013001
HTSeq	Anders et al., 2015	https://htseq.readthedocs.io; RRID: SCR_005514
scran	Lun et al., 2016	http://bioconductor.org/packages/release/bioc/html/scran.html; RRID: SCR_016944
samtools	Li et al., 2009	http://samtools.sourceforge.net/; RRID: SCR_002105
zinbwave	Risso et al., 2018	https://bioconductor.org/packages/release/bioc/html/zinbwave.html
DESeq2	Love et al., 2014	https://bioconductor.org/packages/release/bioc/html/DESeq2.html; RRID:SCR_015687
Seurat	Butler et al., 2018; Stuart et al., 2019	https://satijalab.org/seurat/; RRID:SCR_016341
GSEA	Subramanian et al., 2005; Mootha et al., 2003	https://www.gsea-msigdb.org/gsea/index.jsp; RRID:SCR_003199
STAR Aligner	Dobin et al., 2013	https://github.com/alexdobin/STAR; RRID: SCR_015899
pCLAMP	Molecular Devices	https://www.moleculardevices.com/products/axon-patch-clamp-system/acquisition-and-analysis-software/pclamp-software-suite
Neuroexplorer	Neuroexplorer	https://www.neuroexplorer.com/; RRID:SCR_001818

(Continued on next page)

Continued

REAGENT or RESOURCE	SOURCE	IDENTIFIER
Offline Sorter	Plexon	http://plexon.com/products/offline-sorter; RRID:SCR_000012
Fiji	Fiji	https://fiji.sc; RRID:SCR_002285
Perseus	Perseus	http://maxquant.net/perseus; RRID:SCR_015753

RESOURCE AVAILABILITY

Lead Contact

Further information and requests for resources and reagents should be directed to and will be fulfilled by the Lead Contact, Steven Brown (steven.brown@pharma.uzh.ch).

Materials Availability

No new materials were generated in this work

Data and Code Availability

Data is available in the Zenodo repository (10.5281/zenodo.3946217) and scRNaseq data is in the GEO NCBI repository (GEO: GSE154038). No novel code was generated in this work.

EXPERIMENTAL MODEL AND SUBJECT DETAILS

Mouse strains and husbandry

The following transgenic mouse strains were used: *Syt10-CRE* (Husse et al., 2011), *Vip-CRE* (Taniguchi et al., 2011), *Avp-CRE* (Harris et al., 2014), *Bmal*^{fl} (Storch et al., 2007), *CKIε*^{tau} (Meng et al., 2008), *Grp-CRE* (Sun et al., 2017) and *cFos-GFP* mice (https://www.jax.org/strain/018306).

METHOD DETAILS

All procedures were performed on 6-8 week old male mice backcrossed into a BL6/J background, under 12:12LD in single cage housing with running wheel and food/water available *ad lib*. No mice had been subjected to any previous procedures prior to the start of each experiment. For sleep and running wheel experiments, behavior of mice was recorded continuously. Control mice are siblings from the same litters as experimental animals. All animal experiments were conducted in accordance with applicable veterinary law and approved by the Zürich cantonal veterinary office.

QUANTIFICATION AND STATISTICAL ANALYSIS

Statistical analysis was performed by Student's t test or ANOVA, as specified in figure legends; electrophysiology and patch seq analysis was performed as described below. All statistical analysis and sample sizes are standards for the field, and therefore no specific estimation was made as to whether data met assumptions of the statistical approach.

Immunocytochemistry and cFOS quantification

Mice were perfused with PBS/4% PFA at 4h intervals over 24h of a 12:12LD cycle. Brains were incubated for 90 min in PBS/4% PFA at RT then in 30% sucrose solution in PBS overnight at 4°C, followed by freezing. 40μm coronal slices including the SCN were cut on a Thermo Scientific Microm HM 560 Cryostat. Free-floating SCN slices were washed 6x10 minutes in 10XPBS; incubated 1h in blocking solution (0.1M PBS, 2.5% NGS, 0.3% Triton-X, 0.005% sodium azide) at RT; incubated for 48h at 4°C in blocking solution plus mouse anti-c-Fos (1:50; Santa Cruz Biotechnologies), rabbit anti-VIP (1:600; Peninsula Laboratories) and/or chicken anti-GFP (1:1000); washed 6x in 10xPBS; incubated with 2° antibodies for 1h at RT in the dark; 6x10 min washes in 10xPBS with DAPI added to the last wash (1:1000). Slices were mounted using Vectashield (Vector Laboratories Inc.) then a Z stack was imaged on a Zeiss LSM 710 confocal microscope at 20x magnification. This image was converted into a Z-projection in *ImageJ.* The SCN was identified by DAPI staining, and a threshold was set to eliminate non-specific background outside the SCN. The option *Analyze Particles* was then used to quantify the number of cFOS positive cells within the SCN.

Patch clamp and multi-electrode recordings

Slice preparation

For patch clamp recordings, mice were sacrificed and brains were quickly removed. Brains were mounted on a Vibrating Microtome 7000 from Campden Instruments in a chamber filled with ice-cold and oxygenated artificial cerebrospinal fluid (ACSF)

containing (in mM): 85 NaCl, 75 sucrose, 24 $NaHCO_3$, 2.5 KCl, 1.25 NaH_2PO_4, 0.5 $CaCl_2$, 4 $MgCl_2$ and 25mM glucose saturated with 95% O_2, 5% CO_2 at pH 7.4. 300μm thick coronal slices containing the SCN were incubated for 30 min in ACSF before recording. For the MEA, mice were sacrificed and brains were quickly removed. Brains were cut with the vibratome in ice cold artificial cerebro-spinal fluid (ACSF in mM: NaCl 95; KCl 1.8; KH_2PO_4 1.2; $CaCl_2$ 0.5; $MgSO_4$ 7; $NaHCO_3$ 26; glucose 15; sucrose 50; oxygenated with 95% O_2; 5% CO_2; pH 7.4, measured osmolarity 310 mosmol kg-1). Slices were incubated for at least 1 hour in ACSF at room temperature and then transferred to the recording chamber, perfused continuously with ACSF at room temperature.

For patch-seq experiments, 300μm SCN coronal slices were incubated at 33°C in ice-cold sucrose ACSF (85 mM NaCl, 75 mM sucrose, 2.5 mM KCl, 25 mM glucose, 1.25 mM NaH2PO4, 4 mM MgCl2, 0.5 mM CaCl2 and 24 mM NaHCO3) for 30 min and then kept at room temperature until recording.

Whole cell recordings

Current clamp and voltage clamp recordings were performed using the HEKA EPC-10 amplifier and Patch Master software (HEKA Elektronik, Germany) at RT. Patch pipettes were pulled from borosilicate glass capillaries (DMZ Zeitz Puller), had a resistance of 5-7 MΩ and were filled with a K-gluconate intracellular solution (in mM: K-gluconate 115; KCl 20; Mg-ATP 2, Na_2-ATP 2; Na_2-phosphocreatine 10; GTP 0.3; HEPES 10; measured osmolarity 295-300 mosmol kg-1; pH 7.3-7.4). A GΩ seal was reached and the cell membrane was ruptured under voltage-clamp. Spontaneous activity of SCN neurons was measured in current-clamp mode. After measuring the resting membrane potential and the spontaneous firing frequency, the membrane potential was manipulated by current injection steps (increased steps of 10pA in 15 sweeps and decreased steps of 10pA in 10 sweeps). All the recordings were then analyzed with the software IgorPro and neurons with a difference of 20% in the test pulse were discarded.

For patch-seq experiments, patch pipettes were pulled from borosilicate glass pipettes with filament (Harvard Apparatus; GC150F-10; o.d., 1.5 mm; i.d., 0.86 mm; 10 cm length) and recordings were made with MultiClamp 700B Amplifier (Molecular Devices). Voltage-clamp and current-clamp recording were performed at 33°C with ACSF (126 mM NaCl, 2.5 mM KCl, 10 mM glucose, 1.25 mM NaH2PO4, 2 mM MgCl2, 2 mM CaCl2 and 26 mM NaHCO3). Only neurons with a series resistance lower than 25MΩ were selected for analysis. The firing frequency and resting membrane potential were analyzed with Clampfit 10.7.

Multi-electrode recordings

After 30 minutes incubation, a 300μm slice containing the SCN was placed on a 60pMEA100/30iR-Ti-gr perforated array (Multi Channel Systems). Slices were positioned so that the entire SCN was in contact with the electrode region of the array, and kept in place with a weight, with suction from underneath to maximize contact between the slice and array. Oxygenated ACSF at 34°C ran continuously through the MEA chamber for the duration of the experiment (1.2ml/min inflow/17ml/min outflow + gravity flow inflow/suction outflow at 65). Field potential detected by the MEA at 20,000Hz using *Multi-Channel Experimenter* (Multi Channel Systems). Because of the large file size, recordings were limited to 10 minutes at the start of each hour for the duration of the experiment. Data were analyzed using Offline Sorter (Plexon) as follows: files were run through a butterworth high pass filter at 300Hz and 'spikes' were detected using a threshold of ± 4 Standard Deviations. For each spike the waveform was analyzed and a unit assigned to each unique waveform detected from an individual electrode using the Valley Seeking spike sorting algorithm. Spikes were distinguished from noise by waveform. Data was analyzed using PRISM (GraphPad), and *Perseus* (http://maxquant.net/perseus/.) was used to hierarchically cluster individual unit activity across recordings.

Patch-Seq

Electrophysiology

See above.

Sample collection and processing

Methods and practices were as in (Földy et al., 2016). Briefly, a small amount of intracellular solution (< ~1μl) in the patch pipette was used to record and collect neurons. Cell cytoplasm was aspirated into the patch pipette and immediately transferred into a microtube containing lysis buffer and RNAase inhibitor. The microtube was immediately frozen on dry ice and stored at −80°C until processing using Clontech's SMART-Seq HT kit. cDNA was analyzed on the Fragment Analyzer (Advanced Analytical) before preparing the libraries with Nextera XT DNA Sample Preparation Kit (Illumina). Cells were pooled and sequenced using an Illumina NextSeq 500 system.

Bioinformatics

Raw reads were de-multiplexed and pre-processed using Trimmomatic and Flexbar then aligned to the Ensembl GRCm38 reference transcriptome (Version-2015-06-25), using the STAR aligner [trimLeft = 10, minTailQuality = 15, minAverageQuality = 20 and minReadLength = 30, 'Single-end/paired-end' and 'sense/antisense/both']. Gene counts were calculated using HTSeq. Ensembl gene IDs were converted to gene symbols using the mouse GRCm38 gtf file (ftp://ftp.ensembl.org/pub//release-86/gtf/mus_musculus/Mus_musculus.GRCm38.86.gtf.gz). In the few cases where different Ensembl gene IDs identified the same gene symbol, average gene counts were used.

For quality control, for each cell the total number of unique genes detected with at least 1 mapped read was calculated, and the number of mapped reads. We then calculated the median and median absolute deviation of these 2 values across all cells. Cells that

were more than 3 median absolute deviations below the median in either category were rejected as poor quality. Cells that passed this quality control were then pooled together and normalized using scran (Lun et al., 2016), with sizes 40, 80, 120, 160, 200. Cells that had negative or zero size were removed.

To identify the neuronal subtype of each single neuron, Patch-seq data was projected to the SCN Drop-seq dataset from (Wen et al., 2020). The label transfer function from the R package Seurat was used. For each SCN neuron, a prediction score for each subtype is given. The cell type of a neuron corresponds to the subtype with the highest prediction score.

Differential gene expression analysis was performed using DESeq2 and EdgeR tools with the Zinb-Wave package as recommended for scRNaseq data, and the test = ”LRT” was used for significance. Gene ontology analysis was performed with GSEA tool (Mootha et al., 2003; Subramanian et al., 2005).

Locomotor activity recordings and analysis

Mice were individually housed in cages containing running wheels with *ad libitum* access to food and water. Mice were kept under 12:12LD cycles for the duration of experiments. Data was collected and analyzed using ClockLab (Actimetrics). For LD graphs, behavior was sorted into 30 min bins and the average behavior over 7 days for each mouse was used to generate activity plots, except in optogenetic experiments where a single day of data was used for baseline activity, paired with activity on the day of stimulation. For experiments where a sleep electrode was also in place, mice were placed in specialized sleep boxes allowing continuous recording of EMG, EEG and RW activity. Mice housed in sleep boxes showed increased variability in RW activity owing to differences in running wheel design, RW data from optogenetic stimulations was therefore normalized to allow comparisons between animals.

For siesta timing analysis, the time point of the siesta for each animal was calculated as the 30 min time bin with the lowest level of running wheel activity between ZT18 and ZT23 (as a proxy for the midpoint of the siesta). Where more than one bin had the same low level of activity, an average time was calculated.

Surgical Procedures

AAV Viral injections

To target the SCN, mice anaesthetized with isofluorane were injected with 750nl at 150nl/min of a 1:1 dilution of virus with PBS at ± 0.18mm x; −0.46mm y; −5.9mm z relative to bregma using a Kopf Stereotaxic instrument. In all experiments, mice were returned to the running wheel cage post injection, and allowed to recover while recording running wheel activity. The following Serotype 5 virus’ were injected: AAV.Flex.TetLC (Foster et al., 2015) (UZH Vector Core, $1x10^{12}$ viral particles/ml); AAV-EF1a-DIO-YFP and AAV-EF1a-DIO-ChETA-EYFP (Gunaydin et al., 2010), AAV-Ef1a-DIO-eNpHR3.0-EYFP (UNC Vector Core, $3.9x10^{12}$ viral particles/ml and $6x10^{12}$ viral particles/ml respectively). Post-experiment, sites of injection were histologically confirmed. Overall accuracy in targeting SCN was > 90%; unsuccessfully targeted animals were excluded from further analyses.

Optrode and sleep electrode implantation

Methods used here are based upon those in (Palchykova et al., 2010). For EEG recordings, mice were implanted epidurally with gold-plated screws (0.9mm diameter) under isoflurane inhalation anesthesia. Ceftriaxone was administered during surgery along with buprenorphine for analgesia, and enrofloxacin was provided in drinking water post surgery. Screws were placed in the right frontal (~1.5mm anterior to bregma, 2mm lateral to the midline) and right parietal hemisphere (~2mm posterior to bregma and 3mm lateral of the midline). Two gold wires (0.2mm diameter) were inserted bilaterally in the neck muscle for EMG recordings. Screws were connected to stainless steel wires and fixed to the skull with dental cement.

For optogenetic experiments, mice were injected with virus encoding the optogenetic tool and chronically implanted with an optrode between the 2 SCN lobes (0x; −0.46y; −6.0z from bregma) simultaneous with sleep electrode implantation.

Optogenetic stimulation and silencing

The optogenetic stimulation protocol was based upon the procedure previously shown to be sufficient to entrain the circadian clock (Jones et al., 2015). Experimental animals were VIP-CRE mice injected with AAV-EF1a-DIO-ChETA-EYFP; controls are sibling VIP-CRE mice injected with AAV-EF1a-DIO-YFP, or siblings of VIP-CRE mice that lack the VIP-CRE transgene, injected with AAV-EF1a-DIO-ChETA-EYFP. For optogenetic silencing, experimental animals were VIP-CRE mice injected with AAV-EF1a-DIO-eNpHR3.0-EYFP.

After recovery from surgery, a patch cord connecting a 473nm laser (activation) or 532nm (silencing) (LaserGlow Technologies) was attached to the ferrule covering the optical fiber. With the patch cord in place, the whole implant was covered and painted to prevent light leakage during stimulation. This was kept in place for the duration of the experiment (multiple stimulations at different times). For stimulation, 1h of 10ms pulses of light stimulation at 5Hz or 10Hz were driven by a Master-9 Pulse stimulator (A.M.P.I.), triggered at the appropriate time. For silencing, 532nm light stimulation was delivered in 2 minute cycles of 1 min on/1 min off for 4h, triggered by a Master-9 Pulse stimulator (A.M.P.I.), triggered at the appropriate time. Individual mice were stimulated every 4-5 days, enough time to recover from the previous stimulation.

In all optogenetic experiments, RW activity and sleep were recorded simultaneously. For analysis, RW activity and sleep on the day of stimulation was compared to baseline where mice, with patch cord attached, were not stimulated. Stimulation experiments ceased when mice damaged the opto-sleep implants, when repeated stimulations caused mice to stop running and/or become arrhythmic under LD conditions, or when mice reached ~24 weeks of age.

Sleep recording and analysis

The EEG and EMG signals were amplified (amplification factor, ~2000), filtered (high pass filter: –3 dB at 0.016 Hz; low pass filter: –3 dB at 40 Hz) sampled with 512Hz, digitally filtered [EEG: low pass finite impulse response (FIR) filter, 25 Hz; EMG: bandpass FIR filter, 20–50 Hz or 10-30Hz], and stored with a resolution of 128 Hz. Data analyses and statistics were performed in Excel, PRISM and the MATLAB software package (MathWorks). Sleep was scored by computer assisted staging (Miladinović et al., 2019) followed by visual inspection according to criteria in (Deboer et al., 1994; Franken et al., 1994).

Neuron, Volume *108*

Supplemental Information

Circadian VIPergic Neurons of the Suprachiasmatic Nuclei Sculpt the Sleep-Wake Cycle

Ben Collins, Sara Pierre-Ferrer, Christine Muheim, David Lukacsovich, Yuchen Cai, Andrea Spinnler, Carolina Gutierrez Herrera, Shao'Ang Wen, Jochen Winterer, Mino D.C. Belle, Hugh D. Piggins, Michael Hastings, Andrew Loudon, Jun Yan, Csaba Földy, Antoine Adamantidis, and Steven A. Brown

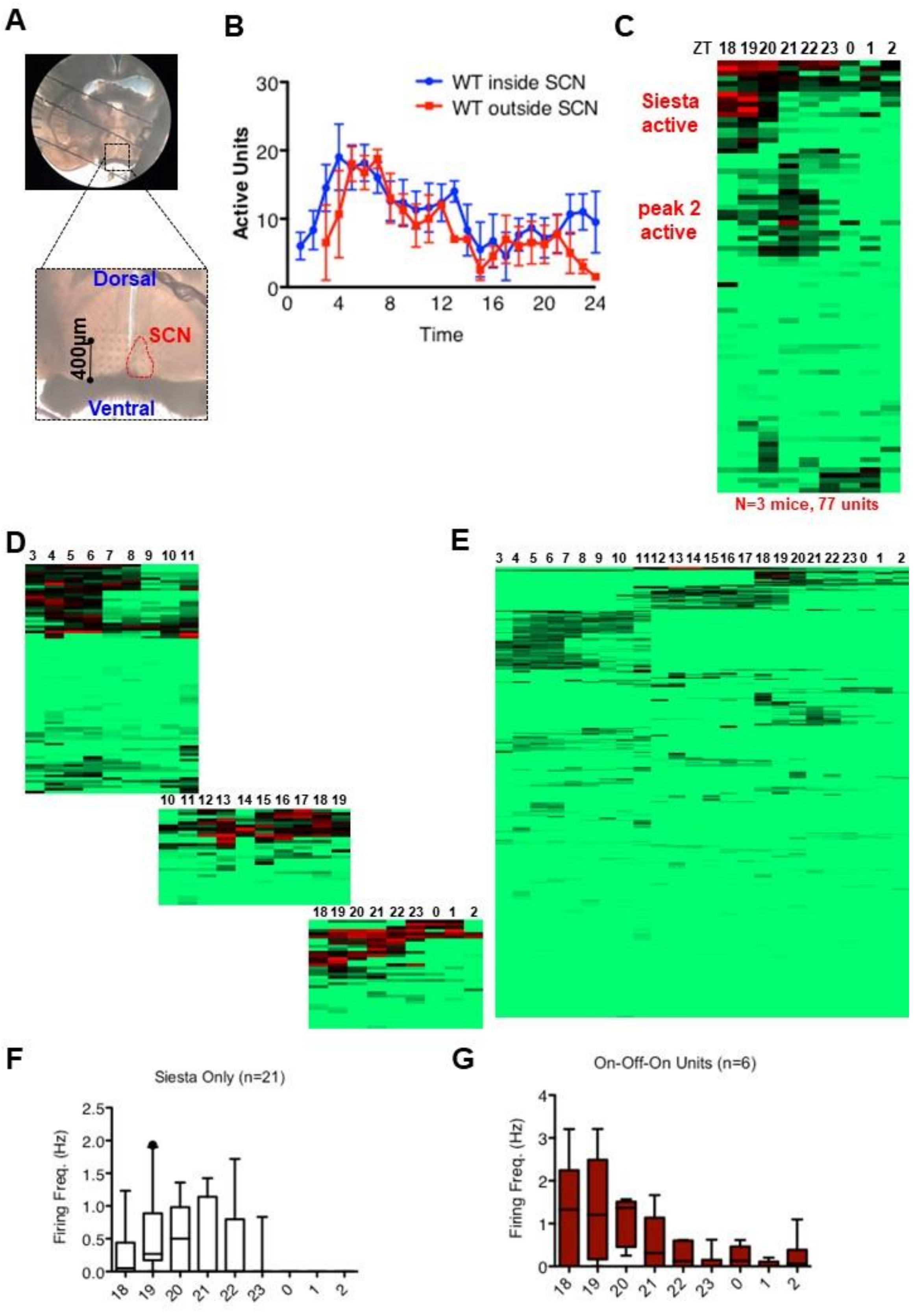

Figure S1: Further characterization of night active SCN neurons

Related to Fig 1

A) Representative image of a typical SCN-containing acute slice *in situ* on the multi-electrode array, with electrodes visible through the slice. **B)** The number of detectable units for a single slice on the MEA varies over time both inside (blue) or outside (red) the SCN. Note that if ~16 electrodes contact the SCN, ~44 are outside the SCN. Thus there are 3x as many active units/electrode within the SCN as outside the SCN. Error bars represent SEM **C)** SCN Electrical activity recorded from the MEA at ZT18-ZT2, plotted as unit activity over time. Note siesta units, some of which become active again at the beginning of the day (quantified in **Fig S1F,G**). Each row represents a unit active at any time point, columns indicate measurement time. Green=0Hz; Red=3Hz. **D)** Example of individual overlapping MEA recordings spanning 24h. Each panel represents a single experiment**. E)** Composite figure showing the timing of all units recorded from all experiments (n=10). **F-G)** Activity of siesta-active SCN units over time; **F)** Most siesta active units are active only during the siesta and are quiescent by ZT23; **G)** a minority of siesta active units are active during the siesta then turn off, then become active again at a much lower firing rate.

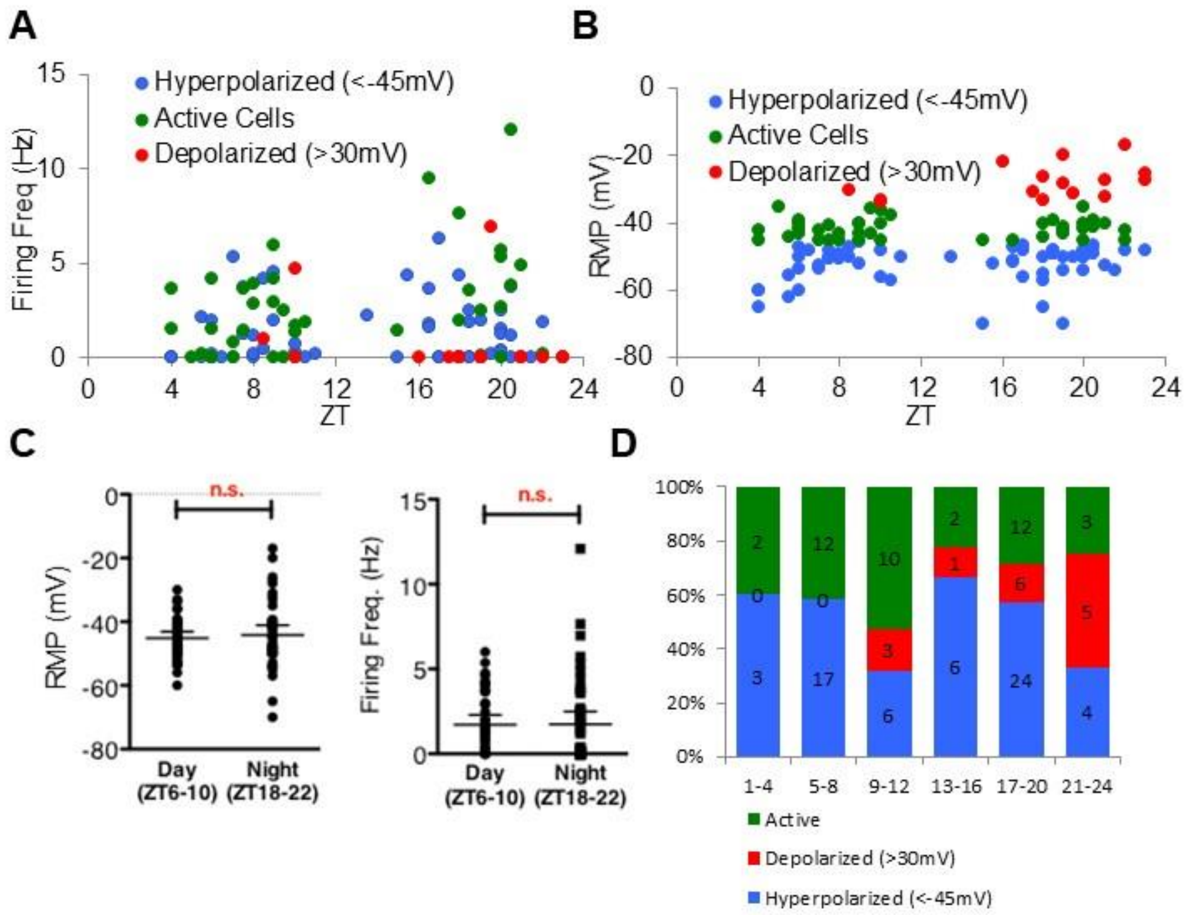

Figure S2: Characterization of night active SCN neurons by patch clamp

Related to Fig 1

A-D) Patch clamp recordings from randomly selected SCN neurons around the clock. Active neurons are shown in green, neurons that were so depolarized that they could not fire (Resting Membrane Potential (RMP)>--30mV) are shown in red, and neurons that were hyperpolarized and could not fire (RMP<-45mV) are shown in blue. **A)** Firing frequency (Hz) of SCN neurons over time. **B)** RMP of SCN neurons over time. **C)** There is no difference in RMP or firing frequency between active SCN neurons recorded during the night (ZT12-24) or day (ZT0-12). **D)** Percentage distribution of the 3 classes of SCN neuron, with recordings divided into six 4h time bins. During the night, the greatest number of active neurons are detected between ZT17 and 22, roughly coincident with the daily siesta.

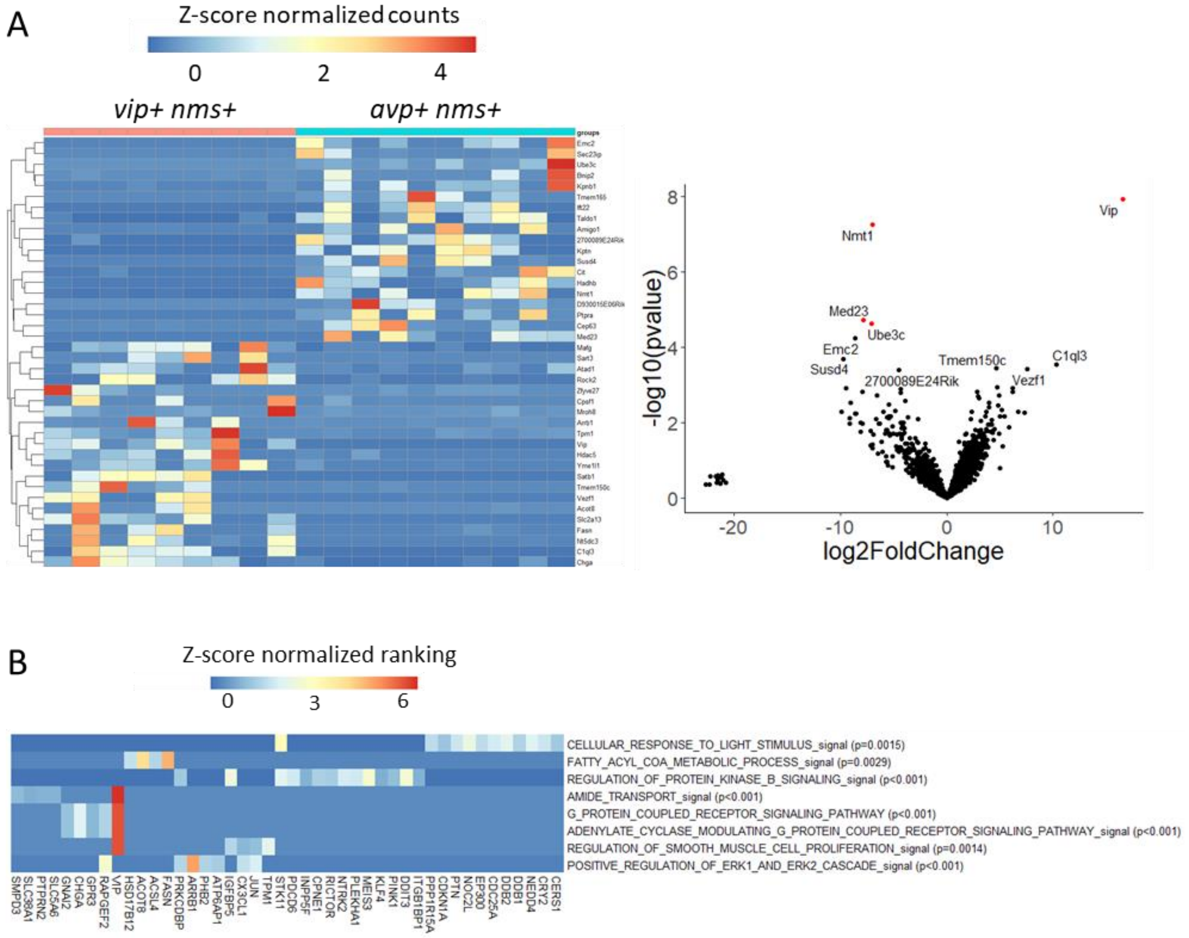

Figure S3: Differential gene expression analysis and pathway analysis between *avp+ nms+* and *vip+ nms+* neurons. Related to Fig 2.

A) Gene expression differences between *vip+ nms+* and *avp+ nms+* cFOS::GFP+ neurons using DESeq2. **Left,** heatmap of the 40 most differentially expressed genes, z-scored by row. **Right,** volcano plot comparison of gene expression between types. Red dots, transcripts with padj<0.01. Genes enriched in *vip+ nms+* cFOS::GFP+ neurons are located to the right. **B)** Gene Set Enrichment analysis. Heatmap showing genes contributing to the most represented gene ontology terms upregulated in *vip+ nms+* neurons compared to a*vp+ nms+* neurons. Color code represents the gene ranking calculated based on DESEq2 results. Nominal pvalue is indicated for each GO term.

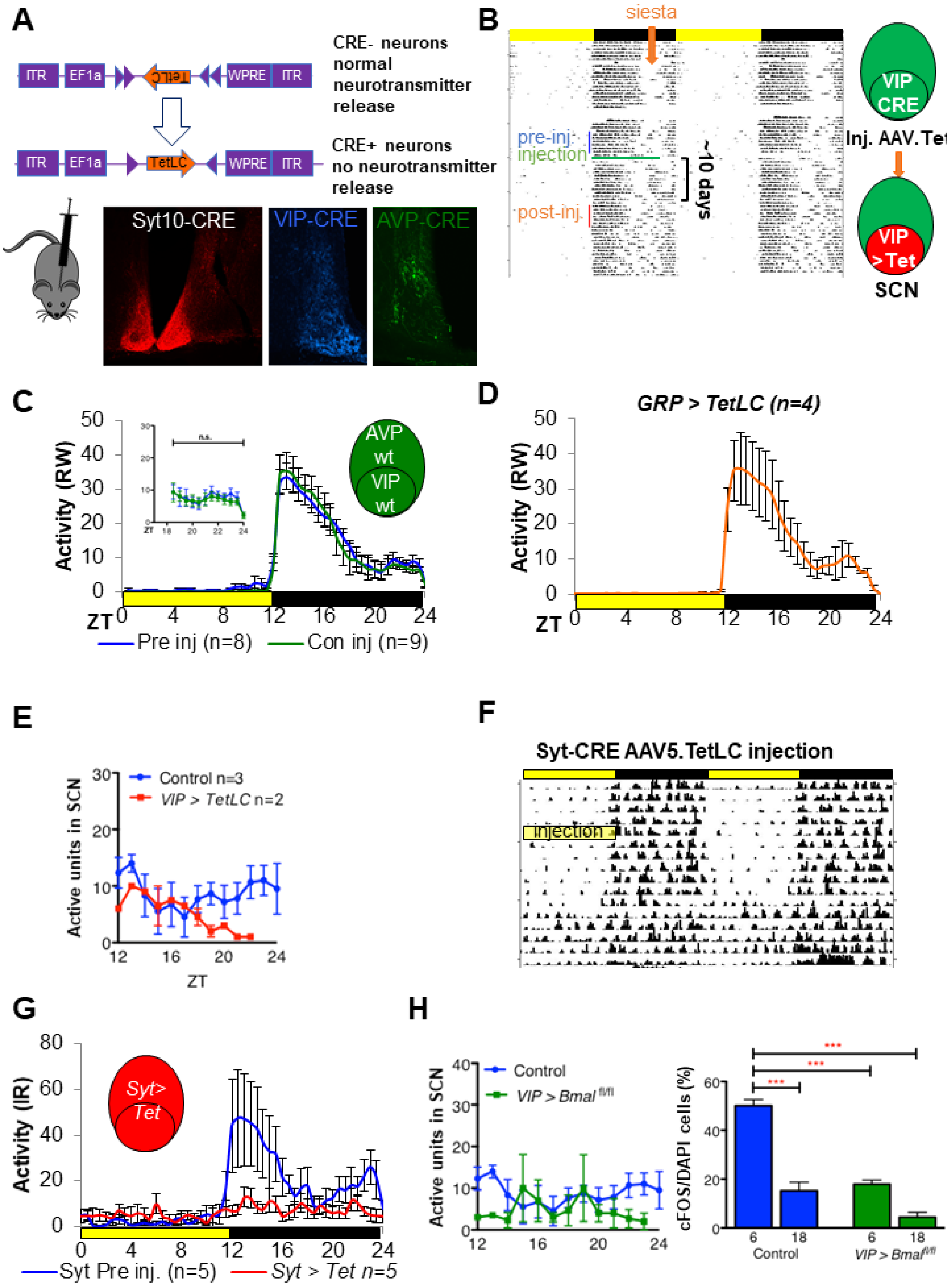

Figure S4: Blocking electrical activity in the SCN affects RW behavior. Related to Fig 3. Error bars represent SEM. ***P<0.005; ****P<0.0001.

A) Top: mice were injected with a CRE-dependent *AAV.Flex.TetLC* virus targeted to the SCN, blocking synaptic transmission from CRE+ SCN neurons. **Bottom:** Examples of *Syt10-CRE*, *Vip-CRE* and *Avp-CRE* driven expression of CRE-dependent YFP in the SCN. **B)** Representative actogram showing the effect of injection of *AAV.Flex.TetLC* into the SCN of *Vip-CRE* mice on RW activity under 12:12 light:dark cycles. Blue bar represents days of data used to plot activity before injection, orange arrow indicates daily siesta, yellow box indicates day of injection, and red bar indicates days used to plot post-injection activity. **C)** Average RW plots showing no effect of injection of control virus into *Vip-CRE* mice. The average of 7 days RW activity in 30 min bins is plotted pre-injection (blue line), compared to 7 days RW activity at least 2 weeks after injection (green line), when the virus should be fully expressed. ($F_{1,180}$=0.6722, n.s.)

D) Injection of *AAV.Flex.TetLC* into the SCN of *Grp-CRE* mice does not disrupt the siesta, as measured by RW activity. **E)** *AAV.Flex.TetLC* reduces VIP neuronal activity within the SCN. Depicted is the number of active units detected from SCN slices on measured in vitro by multielectrode array (MEA) over time. Active units are reduced specifically during the siesta (n=2) in *VIP>TetLC* vs control SCN slices. **E-F)** Synaptic transmission from the SCN is required for normal rhythms under LD cycles. *Syt10-CRE* mice were injected at the SCN with *AAV.Flex.TetLC*, blocking synaptic transmission from all CRE-expressing neurons in the SCN. **E)** Representative actogram of infrared activity under 12:12LD, showing the loss of rhythmicity after injection of *AAV.Flex.TetLC* into the SCN of a *Syt10-CRE* mouse. Yellow box indicates day of injection. **F)** Average activity of infrared behavior under 12:12LD cycles of 5 mice that became arrhythmic after *AAV.Flex.TetLC* injection (blue line pre-injection, red line post injection). Infrared, rather than running wheel activity is shown, as *Syt10-CRE* mice showed reduced to zero running wheel activity post injection. **G)** Left: *VIP>Bmal*$^{fl/fl}$ mice show reduced electrical activity within the SCN, as measured by MEA. Fewer detectable units were observed at night in *VIP>Bmal*$^{fl/fl}$ mice (green) than in controls. Right: cFOS expression is significantly reduced at both ZT6 and ZT18 in *VIP>Bmal*$^{fl/fl}$ SCN slices (green) compared to controls (blue); Statistics: one-way ANOVA with Tukey's post hoc comparisons. $F_{3,11}$=28.79; $P<0.0001$.

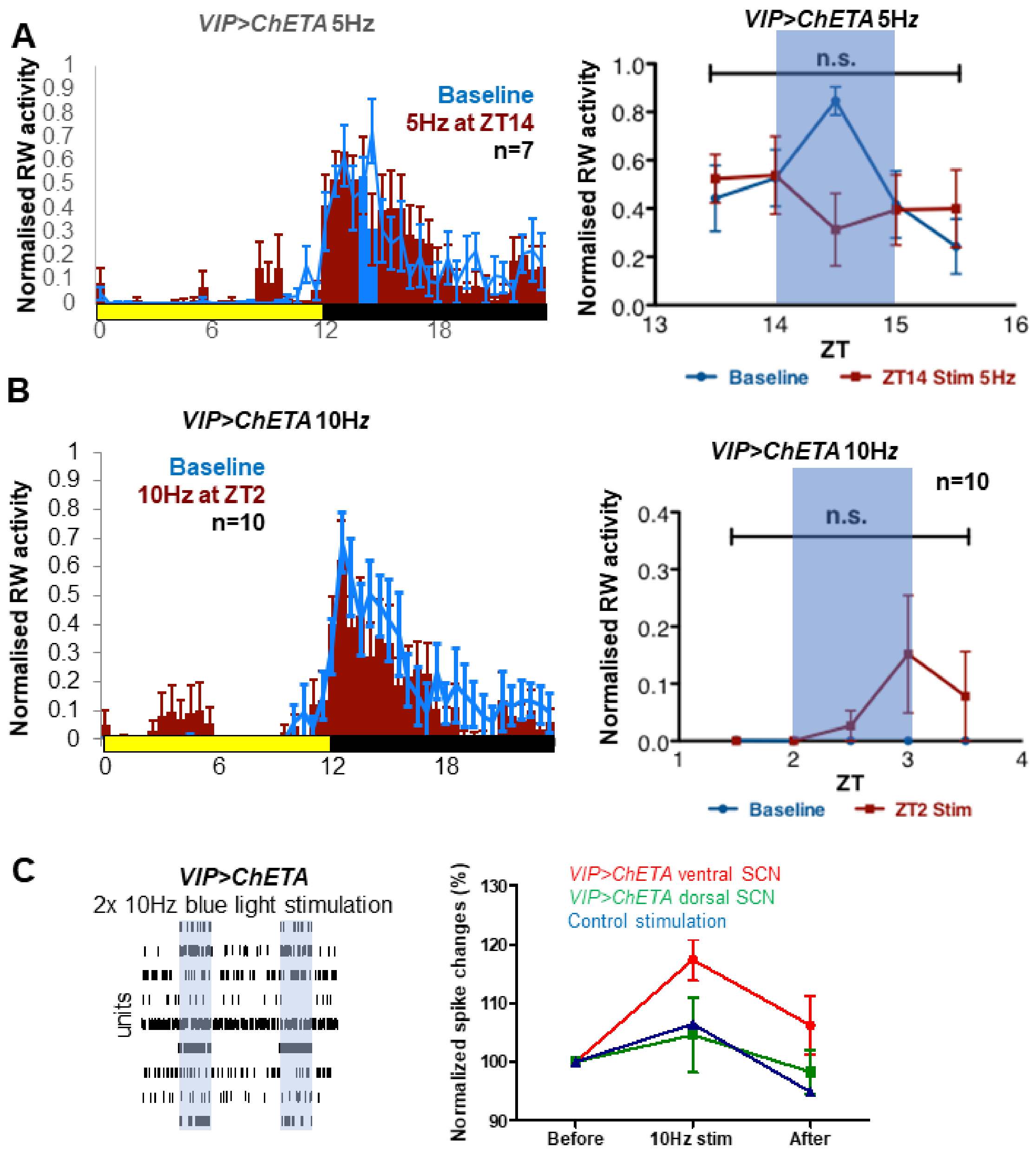

Figure S5: Effect of optogenetic stimulation of VIP+ SCN neurons on RW activity
Related to Fig 3.

Vip-CRE mice were injected at the SCN with the CRE-dependent AAV *VIP>ChETA*, All stimulations (10ms light at 473nm) are for 1hr at the specified frequency at the time specified, and RW activity is plotted in 30 min bins. Blue bars indicate time of stimulation. Error bars represent SEM. All statistical comparisons are by 2 Way ANOVA unless specified. *P<0.05 **A)** Optogenetic stimulation of VIP+ SCN neurons (Fig 3) was repeated at 5Hz. Left: Running wheel activity of *VIP >ChETA* before (baseline, blue line) or after stimulation at ZT14-15 at 5Hz (red and blue bars). Right: Stimulation of *VIP >ChETA* at 5Hz has no significant effect overall on running wheel activity ($P_{1,60}$=0.54, n.s.; however, timepoint 14.5h alone does show significant suppression of activity, p=0-0061 student T-test). **B) left:** RW activity at baseline (blue line) and after stimulation(red bars) at 10Hz at ZT2 (no RW

activity, so blue bar not visible) in *VIP>ChETA* mice. **Right:** statistical comparison of same. Apparent increase in RW activity after stimulation at ZT2 is due to a single mouse (1 of 10 included in plot) increasing running after stimulation; overall there is no significant effect on RW activity due to VIP+ SCN neuron activation at ZT2 ($F_{1,88}$=1.135, n.s.). **C)** MEA recording of *VIP>ChETA* SCN slice before, during (blue shading) and after 10Hz stimulation with 473nm light. **Left,** sample units responding to optogenetically driven activity or indifferent to it. (Units mirroring the 10Hz stimulation itself are not depicted.) **Right,** quantification of all units detected, shown as a percentage of baseline activity for dorsal (green) and ventral (blue) units, and for a control SCN slice lacking ChETA.

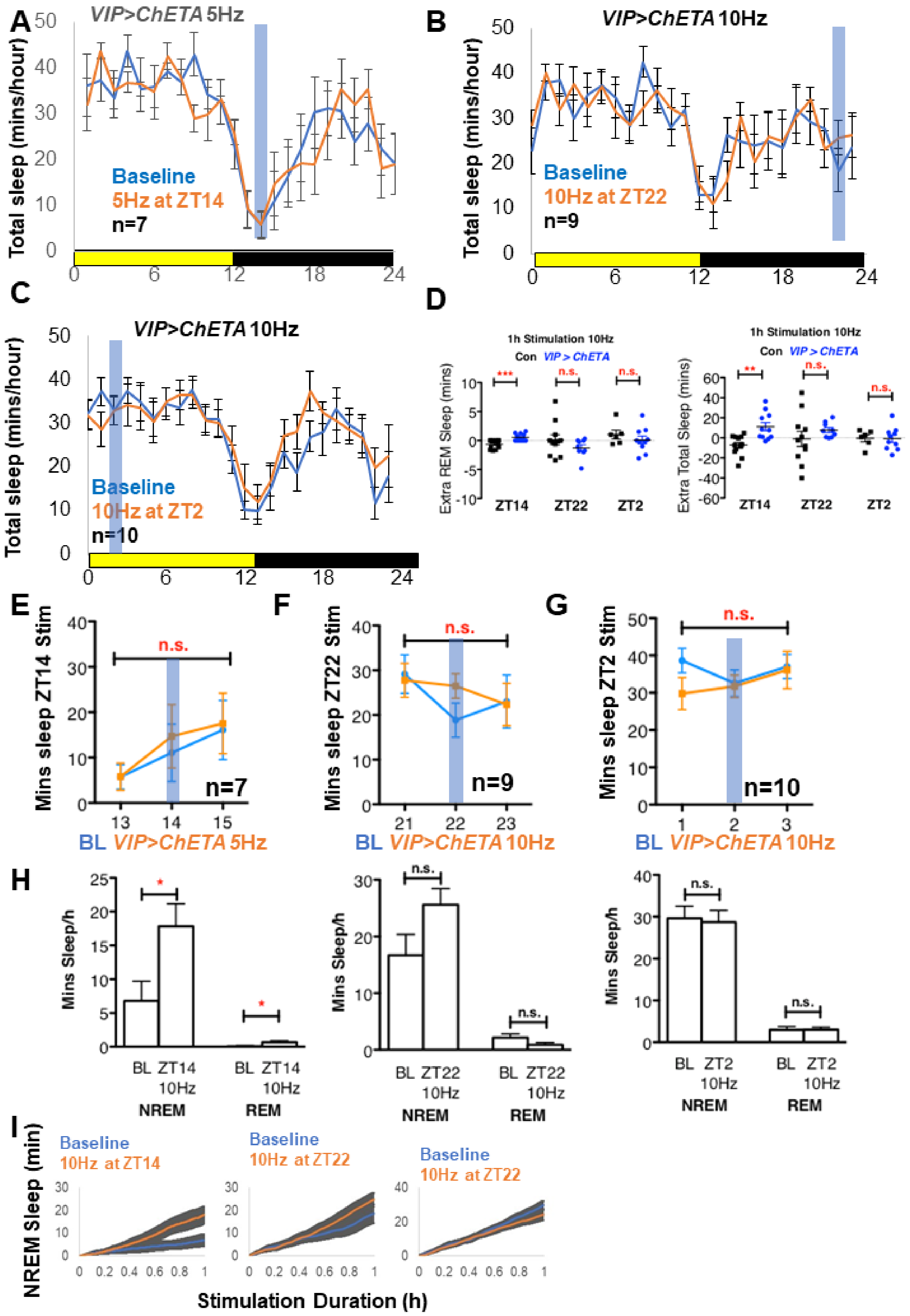

Fig S6: VIP+ SCN neurons regulate sleep during the dark phase

Related to Fig 4.

A-C) Sleep was recorded simultaneously with RW activity during optogenetic stimulations in *VIP>ChETA* mice. Total sleep is plotted in 1h bins for baseline (blue) or on the day of optogenetic stimulation of *VIP>ChETA* mice at 473nm/1h/10Hz (orange). **A)** Stimulation at 5Hz at ZT14 (no effect on sleep) **B)** Stimulation at 10Hz at ZT22 (small, non-significant increase in sleep) **C)** Stimulation at 10Hz at ZT2 (no effect on sleep). Time of stimulation is indicated by blue bar. Error bars represent SEM. Statistical comparisons by 2-tailed Student's t-test unless otherwise specified *P<0.05; **P<0.01; ***P<0.005; Yellow/Black bars represent 12:12LD cycle. **D)** Change in REM and total sleep during stimulation at ZT14, ZT22 or ZT2. (NREM in Fig 4D.) Control, black; *VIP>ChETA*, blue. Note that total and REM sleep are significantly increased in *VIP>ChETA* mice during stimulation at ZT14, but not at ZT22 or ZT2. At ZT22 there is a reduction in the variance in Total Sleep between mice [F test to compare variance: Total sleep $F_{10,8}$=11.17, P<0.01; REM sleep $F_{10,8}$=3.401, n.s.; no differences in variance at ZT22 or ZT2]. **E)** Stimulation of VIP+ SCN neurons at 5Hz had no effect on total sleep at ZT14 (2 way ANOVA, $F_{1,36}$=0.1377) **F)** Stimulation of VIP+ SCN neurons at 10Hz had no effect on total sleep at ZT22 ($F_{1,48}$=0.2775) or **G)** ZT2 ($F_{1,54}$=1.311). **H)** Minutes of NREM and REM sleep were calculated for baseline and during stimulation at ZT14, ZT22 and ZT2 in *VIP>ChETA* mice. Both NREM and REM sleep are significantly increased upon 1h 10Hz stimulation at ZT14. **I)** Cumulative NREM sleep is plotted compared to baseline for *VIP>ChETA* mice during 1h stimulation at ZT14, 22 or 2.

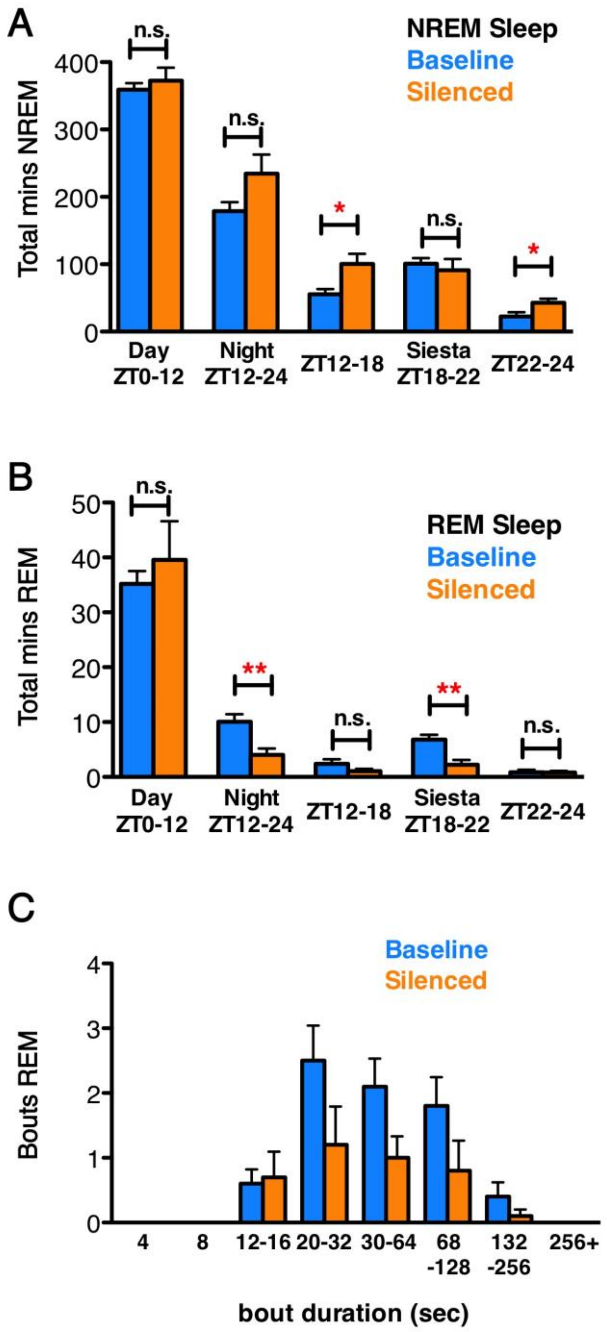
A
NREM Sleep
Baseline
Silenced
Total mins NREM
400
300
200
100
0
n.s.
n.s.
*
n.s.
*
Day
ZT0-12
Night
ZT12-24
ZT12-18
Siesta
ZT18-22
ZT22-24
B
REM Sleep
Baseline
Silenced
Total mins REM
50
40
30
20
10
0
n.s.
**
n.s.
**
n.s.
Day
ZT0-12
Night
ZT12-24
ZT12-18
Siesta
ZT18-22
ZT22-24
C
Baseline
Silenced
Bouts REM
4
3
2
1
0
4
8
12-16
20-32
30-64
68
-128
132
-256
256+
bout duration (sec)

Figure S7: Blocking synaptic transmission from VIP+ SCN neurons disrupts NREM and REM sleep. Related to Fig 4.

A-B) The amount of NREM **(A)** and REM sleep **(B)** during baseline (blue) and post-silencing (orange) sleep recordings, is quantified. There is no overall difference in NREM sleep between baseline and post-silencing recordings during the day or night, but REM sleep is significantly reduced at night in post-silencing recordings. NREM sleep is significantly increased from ZT12-18 and 22-24 (but not 18-22), whilst REM sleep is significantly decreased from ZT18-22, during the siesta (n=5).

C) The number of REM sleep bouts for baseline (blue) and post-silencing (orange) sleep recordings. There is a significant reduction in the number of long REM bouts after silencing of VIP neurons. All comparisons by 2 tailed Student's t-test *P<0.05, **P<0.01

	predicted.id	Prediction score for: *cck+c1ql3+*	*grp+vip+*	*avp+nms+*	*vip+nms+*	*cck+bdnf+*
**C1**	*avp+nms+*	0.021353623	0.00026924	0.571027769	0.400841347	0.006508021
**C2**	*avp+nms+*	0.193437347	0	0.615400778	0.034996949	0.156164926
**C3**	*avp+nms+*	0.05247504	0.001083735	0.903832172	0.03916144	0.003447613
**C4**	*avp+nms+*	0.07961211	0.284657091	0.633412218	0.00231858	0
**C5**	*avp+nms+*	0.101066818	0.011250188	0.615044044	0.237125087	0.035513864
**C6**	*avp+nms+*	0.005699213	0.174127868	0.617451093	0.129800636	0.072921189
**C7**	*avp+nms+*	0.257903376	0	0.59290322	0.014766738	0.134426665
**C8**	*avp+nms+*	0.013169991	0.178772488	0.654811269	0.021025798	0.132220454
**C9**	*vip+nms+*	0.028154312	0.236276879	0.123316132	0.591409949	0.020842729
**C10**	*avp+nms+*	0.221928656	0	0.724242359	0.04157603	0.012252955
**C11**	*vip+nms+*	0.247018916	0.028005422	0.068808226	0.656167436	0
**C12**	*grp+vip+*	0.00043107	0.917710029	0.025511705	0.055070915	0.001276281
**C13**	*vip+nms+*	0.172252056	0.120785151	0.042369427	0.589388112	0.075205254
**C14**	*vip+nms+*	0.01590468	0.00916313	0.241732322	0.725261153	0.007938715
**C15**	*vip+nms+*	0.172777742	0.053196529	0.176805979	0.586531736	0.010688015
**C16**	*vip+nms+*	0.032138468	0.023151609	0.026930338	0.917779585	0
**C17**	*vip+nms+*	0.104622129	0.161502066	0.183401159	0.550474646	0
**C18**	*vip+nms+*	0.26096296	0.019058914	0.024862168	0.695115958	0
**C19**	*avp+nms+*	0.056619818	0.137631003	0.605896805	0.005711688	0.194140686
**C20**	*vip+nms+*	0.033389867	0.167181893	0.244379047	0.555049193	0

Table S1. Prediction scores for collected cFOS-GFP+ neurons. Related to Fig 2.

SCN slices were collected from cFOS::GFP mice between ZT12-24. GFP+ neurons were identified visually, and electrical activity was recorded in different neurons by patch clamp. Cell contents of these neurons were then collected subjected to single-cell RNA sequencing (scSEQ). Transcriptomes

were matched against the scSEQ atlas of SCN neurons (Wen et al., 2020). Prediction scores for each neuron for each of the 5 classes in Wen et al are shown. 0=no transcriptome similarity in discriminating genes; 1=complete transcriptome similarity in discriminating genes. The subtype with the highest prediction score for each cell is the predicted neuron ID.

Cell	Celltype	ZT	C (pF)	iR (MΩ)	sR (MΩ)	RMP (mV)	FF (Hz)
**C1**	*avp+nms+*	15	17.5	749	22.2	-48	5.03
**C2**	*avp+nms+*	15	7.6	594	21.8	-57.9	6.30
**C3**	*avp+nms+*	15	17.0	725	16.5	-53	4.04
**C4**	*avp+nms+*	15.5	27.9	514	21.3	-41	10.61
**C5**	*avp+nms+*	15.5	11.6	569	15.6	-35	0.59
**C6**	*avp+nms+*	15.5	31.3	347	15.9	-58	0.00
**C7**	*avp+nms+*	16	14.1	2708	24.1	-61	2.02
**C8**	*avp+nms+*	16	19.4	3261	23.3	-57	0.08
**C9**	*vip+nms+*	16	12.8	938	16.5	-50	2.87
**C10**	*avp+nms+*	16.5	17.3	2591	19.7	-60	0.47
**C11**	*vip+nms+*	16.5	12.3	843	15.1	-54	5.66
**C12**	*grp+vip+*	17.5	13.0	1242	22.6	-63	1.11
**C13**	*vip+nms+*	17.5	11.3	583	17.1	-53	0.13
**C14**	*vip+nms+*	17.5	13.3	3194	17.6	-58	1.84
**C15**	*vip+nms+*	17.5	12.0	1676	18.9	-46	2.63
**C16**	*vip+nms+*	18	14.7	1376	18.0	-47	1.55
**C17**	*vip+nms+*	18.5	13.0	1581	16.1	-45	2.79
**C18**	*vip+nms+*	19	20.0	690	18.5	-47	3.71
**C19**	*avp+nms+*	19	31.1	556	13.2	-49	3.50
**C20**	*vip+nms+*	19.5	12.3	494	22.4	-57	6.65

Table S2. Electrophysiological properties of SCN neurons from Patch-seq data. Related to Fig 2

Membrane Capacitance (pF), input Resistance (MΩ) and series Resistance (MΩ) were measured in voltage-clamp mode. Resting Membrane Potential (mV) and Firing Frequency (Hz) were measured in current-clamp mode.
